# Cryptic inoviruses revealed as pervasive in bacteria and archaea across Earth’s biomes

**DOI:** 10.1038/s41564-019-0510-x

**Published:** 2019-07-22

**Authors:** Simon Roux, Mart Krupovic, Rebecca A. Daly, Adair L. Borges, Stephen Nayfach, Frederik Schulz, Allison Sharrar, Paula B. Matheus Carnevali, Jan-Fang Cheng, Natalia N. Ivanova, Joseph Bondy-Denomy, Kelly C. Wrighton, Tanja Woyke, Axel Visel, Nikos C. Kyrpides, Emiley A. Eloe-Fadrosh

**Affiliations:** 1 DOE Joint Genome Institute, Walnut Creek, CA USA; 2 Department of Microbiology, Institut Pasteur, Paris, France; 3 Department of Soil and Crop Sciences, Colorado State University, Fort Collins, CO USA; 4 Department of Microbiology and Immunology, University of California, San Francisco, San Francisco, CA USA; 5 Department of Earth & Planetary Sciences, University of California, Berkeley, Berkeley, CA USA; 6 Quantitative Biosciences Institute, University of California, San Francisco, San Francisco, CA USA

**Keywords:** Archaea, Bacteriophages, Environmental microbiology, Phage biology, Metagenomics

## Abstract

Bacteriophages from the *Inoviridae* family (inoviruses) are characterized by their unique morphology, genome content and infection cycle. One of the most striking features of inoviruses is their ability to establish a chronic infection whereby the viral genome resides within the cell in either an exclusively episomal state or integrated into the host chromosome and virions are continuously released without killing the host. To date, a relatively small number of inovirus isolates have been extensively studied, either for biotechnological applications, such as phage display, or because of their effect on the toxicity of known bacterial pathogens including *Vibrio cholerae* and *Neisseria meningitidis*. Here, we show that the current 56 members of the *Inoviridae* family represent a minute fraction of a highly diverse group of inoviruses. Using a machine learning approach leveraging a combination of marker gene and genome features, we identified 10,295 inovirus-like sequences from microbial genomes and metagenomes. Collectively, our results call for reclassification of the current *Inoviridae* family into a viral order including six distinct proposed families associated with nearly all bacterial phyla across virtually every ecosystem. Putative inoviruses were also detected in several archaeal genomes, suggesting that, collectively, members of this supergroup infect hosts across the domains Bacteria and Archaea. Finally, we identified an expansive diversity of inovirus-encoded toxin–antitoxin and gene expression modulation systems, alongside evidence of both synergistic (CRISPR evasion) and antagonistic (superinfection exclusion) interactions with co-infecting viruses, which we experimentally validated in a *Pseudomonas* model. Capturing this previously obscured component of the global virosphere may spark new avenues for microbial manipulation approaches and innovative biotechnological applications.

## Abstract (web summary)

A machine learning approach was used to recover over 10,000 inovirus-like sequences from existing microbial genomes and metagenomes, consequently proposing the reclassification of the *Inoviridae* family to a viral order, and uncover the previously unrecognized diversity of these viruses across hosts and environments.

## Main

**Fig. 1:**
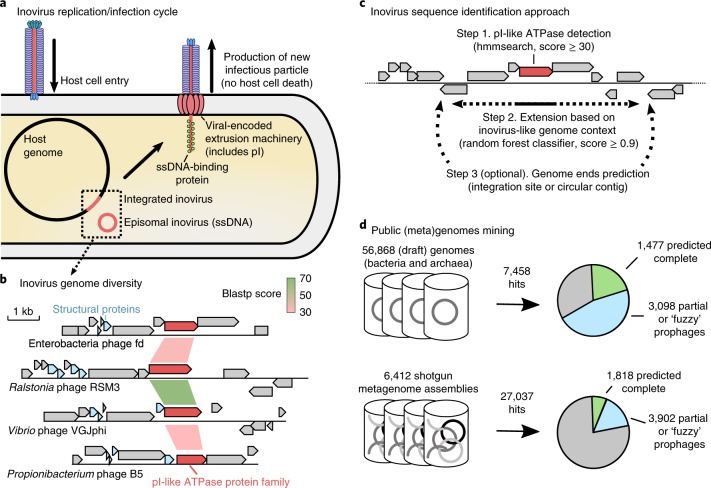
Overview of inovirus infection cycle, diversity and sequence detection process. **a**, Schematic of the inovirus persistent infection cycle and virion production. Inovirus genomes and particles are not to scale relative to the host cell and genome. ssDNA, single-stranded DNA. **b**, Comparison of selected inovirus genomes from isolates. The pI-like genes (the most conserved genes) are coloured in red, and sequence similarity between these genes (based on blastp) is indicated with coloured links between genomes. Putative structural proteins that can be identified based on characteristic features (gene length and presence of a TMD) are coloured in blue. Other genes are coloured in grey. **c**, Representation of the custom inovirus detection approach. The pI-like ATPase gene is coloured in red and other genes are coloured in grey. Dotted arrows indicate the region around pI-like genes that were searched for signs of an inovirus-like genome context and attachment site (see Supplementary Notes). **d**, Results of the search for inovirus sequences in prokaryote genomes and assembled metagenomes, after exclusion of putative false positives through manual inspection of predicted pI proteins (see Supplementary Notes). Predictions for which genome ends could be identified are indicated in green, while predictions without clear ends (that is, partial genomes or ‘fuzzy’ prophages with no predicted att site) are in blue, adding up to 10,295 curated predictions in total. Sequences for which no inovirus genome could be predicted around the initial pI-like gene are in grey. See also Supplementary Figs. 1–3.

Inoviruses, bacteriophages from the *Inoviridae* family, exhibit unique morphological and genetic features. While the vast majority of known bacteriophages carry double-stranded DNA (dsDNA) genomes encapsidated into icosahedral capsids, inoviruses are instead characterized by rod-shaped or filamentous virions, circular single-stranded DNA genomes of ~5–15 kb and a chronic infection cycle^1–3^ (Fig. 1a). Owing to their unique morphology and simple genome amenable to genetic engineering, several inoviruses are widely used for biotechnological applications, including phage display or as drug delivery nanocarriers^4–7^. Ecologically, cultivated inoviruses are known to infect hosts from only 5 bacterial phyla and 10 genera but can have significant effect on the growth and pathogenicity of their host^8–10^. For instance, an inovirus prophage, CTXphi, encodes and expresses the major virulence factor of toxigenic *Vibrio cholerae*^11,12^, whereas in other bacterial hosts, including *Pseudomonas*, *Neisseria* and *Ralstonia*, inovirus infections indirectly influence pathogenicity by altering biofilm formation and host colonization abilities^8,13–16^.

Despite these remarkable properties, their elusive life cycle and peculiar genomic and morphological properties have hampered systematic discovery of additional inoviruses: to date, only 56 inovirus genomes have been described^17^. Most inoviruses do not elicit negative effects on the growth of their hosts when cultivated in the laboratory and can thus easily evade detection. Furthermore, established computational approaches for the detection of virus sequences in whole-genome shotgun sequencing data are not efficient for inoviruses because of their unique and diverse gene content^18–20^ (Fig. 1b). Finally, inoviruses are probably undersampled in viral metagenomes due to their long, flexible virions with low buoyant density^21,22^.

Here, we unveil a substantial diversity of 10,295 inovirus sequences, derived from a broad range of bacterial and archaeal hosts, and identified through an exhaustive search of 56,868 microbial genomes and 6,412 shotgun metagenomes using a custom computational approach to identify putative inovirus genomes. These sequences reveal that inoviruses are far more widespread, diverse and ecologically pervasive than previously appreciated, and provide a robust foundation to further characterize their biology across multiple hosts and environments.

## Results

### Inoviruses are highly diverse and globally prevalent

To evaluate the global diversity of inoviruses, an analysis of all publicly available inovirus genomes was first conducted to identify characteristic traits that would enable automatic discovery of divergent inovirus sequences (Supplementary Table 1). Across the 56 known *Inoviridae* genomes, the gene encoding the morphogenesis (pI) protein, an ATPase of the FtsK–HerA superfamily, represented the only conserved marker gene (Fig. 1a,b and Supplementary Fig. 1). However, three additional features specific of inovirus genomes could be defined: (1) short structural proteins (30–90 amino acids) with a single predicted transmembrane domain (TMD; Supplementary Table 1), (2) genes either functionally uncharacterized or similar to other inoviruses, and (3) shorter genes than those in typical bacterial or archaeal genomes (Supplementary Fig. 2A). These features were used to automatically detect inovirus sequences through a two-step process (Fig. 1b). First, pI-like proteins are detected through a standard hidden Markov model (HMM)-based similarity search. Then, a random forest classifier trained on genomes of isolate inoviruses and manually curated prophages used these genome features to identify inovirus sequences from the background host genome. This approach yielded 92.5% recall and 99.8% precision on our manually curated reference set (Fig. 1c, Supplementary Fig. 2 and Supplementary Notes).

This detection approach was applied to 56,868 bacterial and archaeal genomes and 6,412 metagenomes publicly available from the Integrated Microbial Genomes (IMG) database^23^ (Supplementary Table 2). After manual curation of edge cases and removal of detections not based on a clear inovirus-like ATPase, a total of 10,295 sequences were recovered (Fig. 1d, Supplementary Fig. 3 and Supplementary Notes). From these, 5,964 distinct species were identified using genome-wide average nucleotide identity (ANI), and only 38 of these included isolate inovirus genomes. About one-third of these species (30%) encoded an ‘atypical’ morphogenesis gene, with an amino-terminal instead of carboxy-terminal TMD (Supplementary Fig. 3). Although this atypical domain organization has been observed in four isolate species currently classified as inoviruses, some of these inovirus-like sequences might eventually be considered as entirely separate groups of viruses. Sequence accumulation curves did not reach saturation, highlighting the large diversity of inoviruses yet to be sampled (Supplementary Fig. 4).

**Fig. 2:**
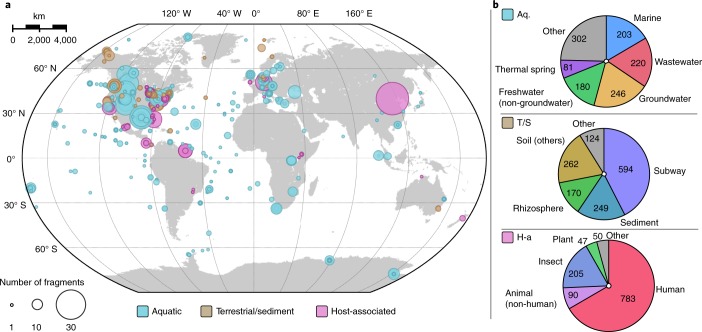
Geographical and biome distribution of inovirus sequences detected in metagenomes. **a**, Repartition of samples for which one or more inovirus sequence(s) was detected. Each sample is represented by a circle proportional to the number of inovirus detections and coloured according to their ecosystem type. **b**, Breakdown of the number of inovirus detections by ecosystem subtype for each major ecosystem. A more detailed ecosystem distribution of each proposed inovirus family is presented in Supplementary Fig. 7. Aq., aquatic; H-a, host-associated; T/S, terrestrial/sediment.

Inovirus sequences were identified in 6% of bacterial and archaeal genomes (3,609 of 56,868) and 35% of metagenomes (2,249 of 6,412). More than half of the species (*n* = 3,675) were exclusively composed of sequences assembled from metagenomes. These revealed that inoviruses are found in every major microbial habitat whether aquatic, soil or human associated, and throughout the entire globe (Fig. 2 and Supplementary Notes). Hence, inoviruses are much more diverse than previously estimated and globally distributed.

### Inoviruses infect a broad diversity of bacterial hosts

**Fig. 3:**
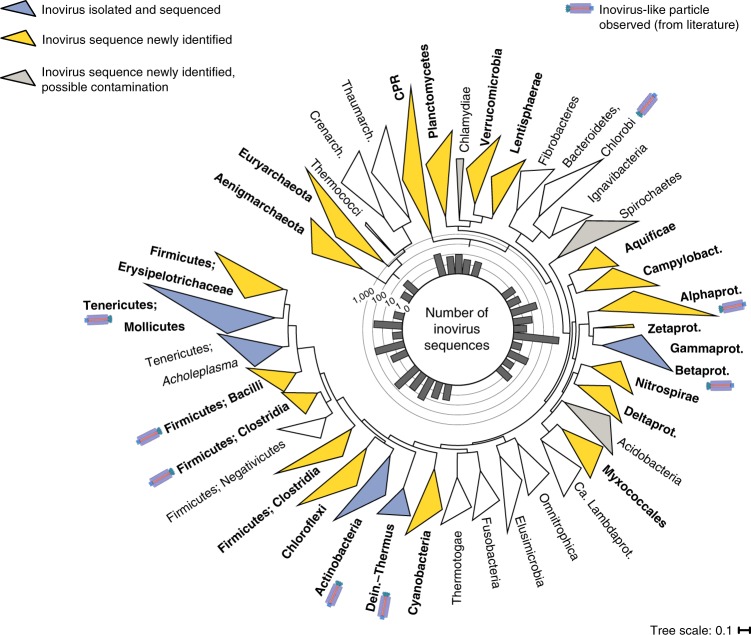
Phylum-wide distribution of inovirus detections across microbial genomes. The bacteria and archaea phylogenetic trees were computed based on 56 universal marker proteins. Monophyletic clades representing a single phylum (or class for proteobacteria) were collapsed when possible, and only clades including ≥30 genomes or associated with an inovirus(es) are displayed. Clades for which one or more inovirus has been isolated and sequenced are coloured in blue, and clades that have not been previously associated with inovirus sequences are coloured in yellow. Clades for which inovirus-like particles had been reported and/or induced are indicated with a filamentous particle symbol. Putative host clades for which inovirus detection might result from sample contamination, that is, no clear host linkage based on an integrated prophage(s) or CRISPR spacer hit(s), are coloured in grey (Supplementary Table 4). Clades robustly associated with inoviruses in this study (that is, one or more detection unlikely to result from sample contamination) are highlighted in bold. The histogram at the centre indicates the total number of inovirus for each clade, on a log_10_ scale. Alphaprot., Alphaproteobacteria; Betaprot., Betaproteobacteria; Ca. Lambdaprot.; ‘*Candidatus* Lambdaproteobacteria’; Campylobact., Campylobacterota; CPR, Candidate Phyla Radiation; Creanarch., Crenarchaeota; Dein.–Thermus, Deinococcus–Thermus; Deltaprot., Deltaproteobacteria; Gammaprot., Gammaproteobacteria; Thaumarch., Thaumarchaeota; Zetaprot., Zetaproteobacteria. See also Supplementary Figs. 4 and 5.

To examine the host range of these inoviruses, we focused on the 2,284 inovirus species directly associated with a host, that is, proviruses derived from a microbial genome (Fig. 3). The majority (90%) of these species were associated with Gammaproteobacteria and Betaproteobacteria, from which most known inoviruses were previously isolated (Supplementary Table 1). However, the range of host genera within these groups was vastly expanded, including clinically and ecologically relevant microorganisms such as *Azotobacter*, *Haemophilus*, *Kingella* or *Nitrosomonas* (Supplementary Table 3). The remaining 412 species strikingly increased the potential host range of inoviruses to 22 additional phyla, including the Candidate Phyla Radiation (Fig. 3). For three of these (Acidobacteria, Chlamydiae and Spirochaetes), only short inovirus contigs were detected, lacking host flanking regions, which would provide confident host linkages. Hence, these contigs could potentially derive from sample contamination (for example, from reagents), and inovirus presence within these phyla remains uncertain (Supplementary Table 4). The notable host expansion is consistent with reported experimental observations of filamentous virus particles induced from a broad range of bacteria, for example, *Mesorhizobium*, *Clostridium*, *Flavobacterium*, *Bacillus* and *Arthrobacter*^24,25^ (Fig. 3).

This large-scale detection of inovirus sequences in microbial genomes also enabled a comprehensive assessment of co-infection, both between different inoviruses and with other types of viruses. In the majority of cases, a single inovirus sequence was detected per genome, with multiple detections mostly found within Gammaproteobacteria, Betaproteobacteria and *Spiroplasma* genomes (Supplementary Fig. 5). Conversely, inovirus prophages were frequently detected along and sometimes colocalized with *Caudovirales* prophages, suggesting that these two types of phages frequently co-infect the same host cell (Supplementary Fig. 5 and Supplementary Notes). Overall, the broad range of bacteria and archaea infected by inoviruses combined with their propensity to co-infect a microbial cell with other viruses and their global distribution indicate that inoviruses probably play an important ecological role in all types of microbial ecosystems.

### Inoviruses sporadically transferred from bacterial to archaeal hosts

**Fig. 4:**
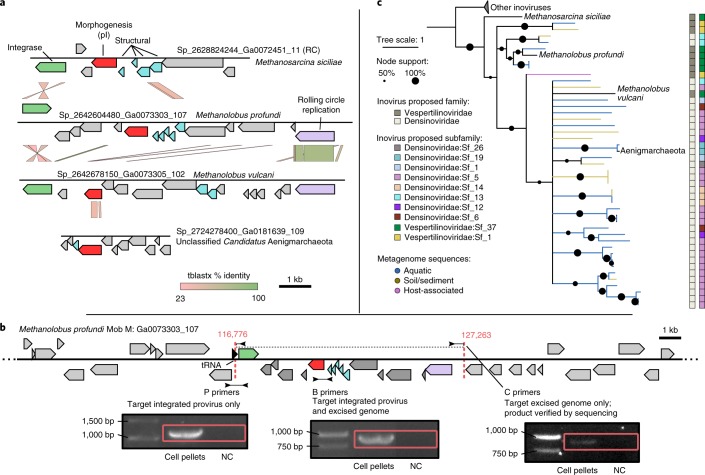
Characterization of archaea-associated inoviruses. **a**, Genome comparison of the four inovirus sequences detected in members of the Methanosarcinaceae family or Aenigmarchaeota candidate phylum. Genes are coloured according to their functional affiliation (light grey indicates ORFan). RC, sequence is reverse complemented. **b**, PCR validation of the predicted inovirus from the archaea host *M.* *profundi* MobM. Three primer pairs were designed and used to amplify across the predicted 5′ insertion site (P primers), within the predicted provirus (B primers) or across the junction of the predicted excised circular genome (C primers). The predicted provirus attachment site is indicated by dotted red lines along with corresponding genome coordinates. Products from C primers were sequenced and aligned to the *M.* *profundi* MobM genome to confirm that they spanned both ends of the provirus in the expected orientation and at the predicted coordinates (see Supplementary Notes and Supplementary Fig. 6). Red boxes indicate the expected product lengths. P and B primer amplifications were repeated twice, and the C primer amplifications were repeated three times, with an identical result obtained for each replicate (Supplementary Fig. 11). NC, no template control. **c**, Phylogenetic tree of archaea-associated inoviruses and related sequences. The tree was built from pI protein multiple alignment with IQ-TREE. Nodes with support of <50% were collapsed. Branches leading to inovirus species associated to a host are coloured in black, and the corresponding host is indicated on the tree. Branches leading to inovirus species assembled from metagenomes are coloured by type of environment. Classification of each inovirus species in proposed families and subfamilies is indicated next to the tree (see Fig. 5).

Although no archaea-infecting inoviruses have been reported so far^26^, some inovirus sequences were associated with members of two archaeal phyla (Euryarchaeota and Aenigmarchaeota), which suggests that inoviruses infect hosts across the entire prokaryotic diversity (Fig. 3). These putative archaeal proviruses encoded the full complement of genes expected in an active inovirus (Fig. 4a and Supplementary Notes). Using PCR, we further confirmed the presence of a circular, excised form of the complete inovirus genome for the provirus identified in the *Methanolobus profundi* MobM genome (Fig. 4b, Supplementary Fig. 6 and Supplementary Notes). This indicates that our predictions in archaeal genomes are probably genuine inoviruses.

Few groups of viruses include both bacteriophages and archaeoviruses. Such evolutionary relationships between viruses infecting hosts from different domains of life might signify either descent from an ancestral virus that infected the common ancestor of bacteria and archaea, or horizontal virus transfer from one host domain to the other^26–28^. Here, the four archaea-associated inoviruses were clearly distinct from most other inoviruses and clustered only with metagenomic sequences in pI phylogeny (Fig. 4c). In addition, they were classified into two different proposed families (see below) corresponding to the two host groups, reflecting clear differences in their gene content (Fig. 4a,c and Supplementary Notes). The high genetic diversity of these archaea-associated inoviruses, combined with the lack of similarity to bacteria-infecting species, suggest that they are not derived from a recent host switch event.

A possible scenario would involve an ancestral group of inoviruses infecting the common ancestor of archaea, as postulated for the double-jelly-roll virus lineage^28^. However, to be confirmed, this hypothesis would require the detection of additional inoviruses in other archaeal clades or an explanation as to why inoviruses were retained only in a handful of archaeal hosts. Instead, on the basis of the current data, a more likely scenario involves ancient and rare events of interdomain inovirus transfer from bacteria to archaea, including possibly to a *Methanosarcina* host for which substantive horizontal transfers of bacterial genes have already been reported^29^.

### Gene content classification reveals six distinct inovirus families

**Fig. 5:**
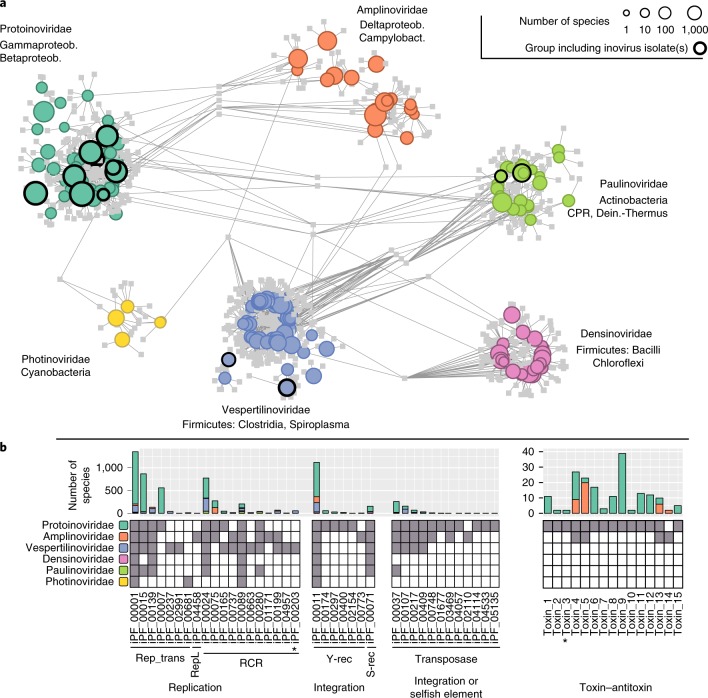
Inovirus genome sequence space and gene content. **a**, The bipartite network links genes represented as PCs in squares to proposed subfamilies represented as circles with a size proportional to the number of species in each candidate subfamily (log_10_ scale), grouped and coloured by proposed family. Proposed subfamilies that include viral isolates are highlighted with a black outline. Candidate subfamilies are connected to PCs when ≥50% of the subfamily members contained this PC or ≥25% for the larger proposed subfamilies (see Methods). **b**, Distribution of iPFs detected in two or more genomes, associated with genome replication, genome integration and toxin–antitoxin systems (see Supplementary Table 5). The presence of at least one sequence from an iPF (column) in a proposed family (row) is indicated with a grey square. Rolling circle replication (RCR) iPFs include only the RCR endonuclease motif, with the exception of iPF_00203 (highlighted with an asterisk), which also includes the C-terminal S3H motif typical of eukaryotic single-stranded DNA viruses. Transposases used by selfish integrated elements are indistinguishable from transposases domesticated by viral genomes using sequence analysis only; hence, these genes are gathered in a single ‘integration or selfish element’ category. All toxin–antitoxin pairs were predicted to be of type II, except for Toxin_3 (highlighted with an asterisk), which was predicted to be type IV. S-rec, serine recombinase; Y-rec, tyrosine recombinase. See also Supplementary Figs. 7–9.

The vast increase of inovirus sequences provided a great opportunity for re-evaluation of the inovirus classification and the development of an expanded taxonomic framework for the large number of inovirus species identified. Similar to other bacterial viruses, especially temperate phages^30^, inovirus genomes display modular organization and are prone to recombination and horizontal gene transfers^31^ (Supplementary Fig. 7). Hence, we opted to apply a bipartite network approach, in which genomes are connected to gene families, enabling a representation and clustering of the diversity based on shared gene content. A similar approach has been previously employed for the analysis of DNA and RNA viruses, and was shown to be efficient in cases in which the genomes to be clustered share only a handful of genes^26,32–34^. Here, this approach yielded 6 distinct groups of genomes divided into 212 subgroups (Fig. 5a and Supplementary Table 3).

A comparison of marker gene conservation between these groups and established viral taxa suggested that the former *Inoviridae* family should be reclassified as an order, provisionally divided into 6 candidate families and 212 candidate subfamilies, with few shared genes across candidate families (Fig. 5a, Supplementary Fig. 7 and Supplementary Notes). Beyond gene content, these proposed families also displayed clearly distinct host ranges as well as specific genome features, particularly in terms of genome size and coding density (Supplementary Fig. 7). Thus, we propose to establish these as candidate families named ‘Protoinoviridae’, ‘Vespertilinoviridae’, ‘Amplinoviridae’, ‘Paulinoviridae’, ‘Densinoviridae’ and ‘Photinoviridae’, on the basis of their isolate members and characteristics (see Supplementary Notes). If confirmed, and compared with currently recognized inoviruses, the genomes reported here would increase diversity by 3 families and 198 subfamilies.

The host envelope organization seems to play an important role in the evolution of inoviruses, which is reflected in their classification: members of the ‘Protoinoviridae’ and ‘Amplinoviridae’ are associated with diderm hosts—that is, Gram-negative bacteria with an outer membrane—whereas the other candidate families are associated with monoderm hosts or hosts without a cell wall (Supplementary Fig. 7). Conversely, no structuring by biome was observed and all proposed families were broadly detected across multiple types of ecosystems. Hence, we propose here a classification of inovirus diversity into six families based on gene content with coherent host ranges and specific genomic features, which strongly suggests that they represent ecologically and evolutionarily meaningful units.

### Inovirus genomes encode an extensive functional repertoire

The extended catalogue of inovirus genomes offers an unprecedented window into the diversity of their genes and predicted functions. Overall, 68,912 proteins were predicted and clustered into 3,439 protein families and 13,714 singletons. This is on par with the functional diversity observed in known *Caudovirales* genomes, the largest order of dsDNA viruses, for which the same number of proteins clustered into 12,285 protein families but only 8,552 singletons (see Methods). A putative function was predicted for 1,133 of the 3,439 inovirus protein families (iPFs). Most of these (>95%) could be linked to virion structure, virion extrusion, DNA replication and integration, toxin–antitoxin systems or transcription regulation (Supplementary Table 5). A total of 51 and 47 distinct iPFs could be annotated as major and minor coat proteins, respectively, with an additional 934 iPFs identified as potentially structural based on their size and presence of a TMD (see Methods). Notably, each candidate inovirus family seemed to be associated with a specific set of structural proteins, including distinct major coat iPFs (Supplementary Fig. 8). Conversely, genome replication and integration-associated iPFs were broadly shared across candidate families (Fig. 5b). This confirms that replication-associated and integration-associated genes are among the most frequently exchanged among viral genomes and with other mobile genetic elements, especially in small single-stranded DNA viruses^35^.

In addition, 15 distinct sets of iPFs representing potential toxin–antitoxin pairs were identified across 181 inovirus genomes, including 10 unaffiliated iPFs that were predicted as putative antitoxins through co-occurrence with a toxin iPF (Fig. 5b and Supplementary Table 5; see Methods). These genes typically stabilize plasmids or prophages in host cell populations, although alternative roles in stress response and transcription regulation have been reported^36^. In addition, toxin–antitoxin systems often affect host cell phenotypes, such as motility or biofilm formation^1^. Here, similar toxin proteins could be associated with distinct and seemingly unrelated antitoxins and vice versa, suggesting that gene shuffling and lateral transfer occur even within these tightly linked gene pairs (Supplementary Fig. 9). All but one toxin–antitoxin pairs were detected in proteobacteria-associated inoviruses, most likely because of a database bias. Thus, numerous uncharacterized iPFs across other candidate families of inoviruses may also encode previously undescribed toxin–antitoxin systems and, more generally, host manipulation mechanisms.

### Inoviruses can both leverage and restrict co-infecting viruses

**Fig. 6:**
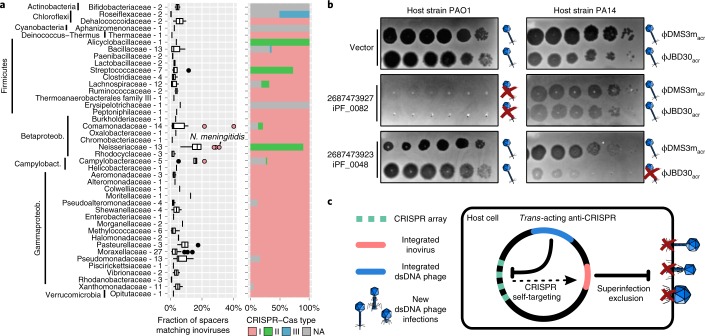
Interaction of inoviruses with CRISPR–Cas systems and co-infecting viruses. **a**, Proportion of the spacers matching an inovirus genome and the corresponding distribution of CRISPR–Cas systems. The proportions are calculated only on hosts with at least one spacer matching an inovirus sequence, with hosts grouped at the family rank (hosts unclassified at this rank were not included). In the boxplot, the lower and upper hinges correspond to the first and third quartiles, respectively, and the whiskers extend no further than ±1.5 times the interquartile range. Outliers identified as values larger than the third quartile plus three times the interquartile range from the complete distribution are highlighted in red. The number of observations is indicated next to each family. **b**, Instances of superinfection exclusion observed when expressing individual inovirus genes in two *P.* *aeruginosa* strains: PAO1 and PA14. From top to bottom: cells were transformed with an empty vector, one expressing gene 2687473927 or one expressing gene 2687473923. For each construct, host cells were challenged with serial dilutions (from left to right) of phages: ϕJBD30 and ϕDMS3m. The formation of plaques (dark circles) indicates successful infection, whereas the absence of plaques indicates superinfection exclusion. Interpretation of infection outcome is indicated to the right of each lane, with successful infection represented by a phage symbol and superinfection exclusion represented by a phage symbol barred by a red cross. Results from additional superinfection exclusion experiments are presented in Supplementary Figs. 10 and 12. All superinfection experiments were conducted twice and produced similar results. **c**, Schematic representation of the possible mutualistic or antagonistic interactions between inovirus prophages (red) and co-infecting *Caudovirales* (blue). Mutualistic interactions include suppression of the CRISPR–Cas immunity, especially for integrated inoviruses targeted by the host cell CRISPR–Cas system (‘self-targeting’). Antagonistic interactions primarily involve superinfection exclusion, in which a chronic inovirus infection prevents a secondary infection by an unrelated virus.

Finally, we investigated potential interactions between persistently infecting inoviruses, other co-infecting viruses, and the host clustered regularly interspaced short palindromic repeats (CRISPR)–CRISPR-associated (Cas) immunity systems. CRISPR–Cas systems typically target bacteriophages, plasmids and other mobile genetic elements^37^. We detected 1,150 inovirus-matching CRISPR spacers across 42 bacterial and 1 archaeal families. These spacers were associated with three types and eight subtypes of CRISPR–Cas systems, indicating that inoviruses are broadly targeted by antiviral defences (Fig. 6a, Supplementary Table 6 and Supplementary Notes). Several host groups, most notably *Neisseria meningitidis*, were clear outliers, that is, they displayed a particularly high ratio of inovirus-derived spacers suggesting a uniquely high level of spacer acquisition and inovirus infection (Fig. 6a). This is particularly notable because inoviruses were recently suggested to increase *N.* *meningitidis* pathogenicity^13^ and hints at conflicting host–inovirus interactions in this specific group.

Next, we examined instances of ‘self-targeting’, that is, CRISPR spacers matching an inovirus integrated in the same host genome. Among the 1,429 genomes that included both a CRISPR–Cas system and an inovirus prophage, only 45 displayed a spacer match(es) to a resident prophage (Supplementary Table 6), suggesting that self-targeting of these integrated elements is lethal and strongly counter-selected^38^. This was confirmed experimentally using the *Pseudomonas aeruginosa* strain PA14 harbouring an integrated inovirus prophage (Pf1), for which the introduction of a plasmid carrying Pf1-targeting CRISPR spacers was lethal (Supplementary Fig. 10a). In the 45 cases of observed self-targeting, the corresponding CRISPR–Cas system is thus probably non-functional or inhibited via an anti-CRISPR (*acr*) locus, as recently described in dsDNA phages^38^. We first evaluated ten hypothetical proteins, and hence candidate Acr proteins, from self-targeted inoviruses infecting *P.* *aeruginosa*; however, none showed Acr activity (Supplementary Notes and Supplementary Fig. 10b). Alternatively, inoviruses could leverage the Acr activity of a co-integrated virus. This hypothesis was further reinforced by the fact that 43 of the 45 self-targeted inoviruses were detected alongside co-infecting dsDNA phages, with 5 of these encoding known *acr* genes (Supplementary Table 6). We confirmed experimentally cross-protection by *trans*-acting Acr in the *P.* *aeruginosa* PA14 model, and observed that co-infection with an *acr*-encoding dsDNA bacteriophage rescued the lethality caused by self-targeted inoviruses (Supplementary Notes and Supplementary Fig. 10a).

While this represents an instance of beneficial co-infection for inoviruses, we also uncovered evidence of antagonistic interactions between inoviruses and dsDNA bacteriophages. Specifically, 2 of the 10 inovirus-encoded hypothetical proteins tested strongly limited infection of *Pseudomonas* cells by different bacteriophages (Fig. 6b, Supplementary Figs. 10c and 12 and Supplementary Notes). This superinfection exclusion effect was found to be host and virus strain dependent, which could drive intricate tripartite coevolution dynamics. Thus, these preliminary observations indicate that inoviruses may not only evade CRISPR–Cas immunity by leveraging the Acr activity of co-integrated phages, but also significantly influence the infection dynamics of unrelated co-infecting viruses through superinfection exclusion (Fig. 6c). Multiple effects of virus–virus interactions on host ecology and evolution have been recently highlighted or proposed, and are the main focus of a nascent ‘sociovirology’ field^39^. Given their broad host range (Fig. 3), frequent detection alongside non-inovirus prophages (Supplementary Fig. 5), extended host cell residence time and the experimental results presented here, inoviruses could be driving many of these interactions and are undeniably important to consider in this framework.

## Discussion

Taken together, the results presented here call for a complete re-evaluation of the diversity and role of inoviruses in nature. Collectively, inoviruses are distributed across all biomes and display an extremely broad host range spanning both prokaryotic domains of life. Comparative genomics revealed evidence of longstanding virus–host codiversification, leading to strong partitioning of inovirus diversity by host taxonomy, high inovirus prevalence in several microbial groups, including major pathogens, and potential interdomain transfer. Even though small (5–20 kb), their genomes encode a large functional diversity shaped by frequent gene exchange with unrelated groups of viruses, plasmids and transposable elements. Some of the many uncharacterized inovirus genes probably encode molecular mechanisms at the interface of virus–host and virus–virus interactions, such as modulators of the CRISPR–Cas systems, superinfection exclusion genes or toxin–antitoxin modules. This expanded and restructured catalogue of 5,964 distinct inovirus genomes thus provides a renewed framework for further investigation of the different effects that inoviruses have on microbial ecosystems, and exploration of their unique potential for biotechnological applications and manipulation of microorganisms.

## Methods

### Construction of an *Inoviridae* genome reference set

Genome sequences affiliated to *Inoviridae* and ≥2.5 kb were downloaded from NCBI Genbank and RefSeq on 14 July 2017 (refs. ^40,41^). These were clustered at 98% ANI to remove duplicates and screened for cloning vectors and partial genomes (Supplementary Table 1). Two of these genomes (*Stenotrophomonas* phage phiSMA9, NC_007189, and *Ralstonia* phage RSS30, NC_021862) presented an unusually long section (≥1 kb) without any predicted gene, associated with a lack of short genes that are typical of *Inoviridae*. For these, genes were predicted de novo using Glimmer^42^ trained on their host genomes (NC_010943 for phiSMA9 and NC_003295 for RSS30) with standard genetic code. Similarly, genes for *Acholeplasma* phage MV-L1 (NC_001341) were predicted de novo using Glimmer with genetic code 4 (*Mycoplasma*/*Spiroplasma*) and trained on the host genome (NC_010163), followed by a manual curation step to integrate both RefSeq-annotated genes and these newly predicted CDS.

Protein clusters (PCs) were computed from these genomes from an all-versus-all blastp of predicted CDS (thresholds: *e* ≤ 0.001, bit score ≥ 30) and clustered with InfoMap^33^. Sequences from these PCs were then aligned with MUSCLE^43^, transformed into an HMM profile and compared with each other using HHSearch^44^ (cut-offs: probability ≥ 90% and coverage ≥ 50%, or probability ≥ 99%, coverage ≥ 20% and hit length ≥ 100). The larger clusters generated through this second step are designated here as iPFs. Only ten PCs were clustered into larger iPFs, but these were consistent with the functional annotation of these proteins. For instance, one iPF combined two PCs both composed of replication initiation proteins.

Marker genes were identified from a bipartite network linking *Inoviridae* genomes to iPFs (Supplementary Fig. 1). Only the genes encoding the morphogenesis (pI) protein represented good candidates for a universally conserved gene across all members of the *Inoviridae*, and HMM profiles were built for the three pI iPFs. To optimize these profiles, sequences were first clustered at 90% amino acid identity with cd-hit^45^, then aligned with MUSCLE^43^ and the profile generated with hmmbuild^46^.

These reference genomes were also used to evaluate the detection of the *Inoviridae* structural proteins based on protein features beyond sequence similarity (see Supplementary Notes). Here, signal peptides were predicted using SignalP in both Gram-positive and Gram-negative modes^47^, and TMDs were identified with TMHMM^48^.

### Search for inovirus in microbial genomes and metagenomes

Proteins predicted from 56,868 microbial genomes publicly available in the IMG as of October 2017 (Supplementary Table 2) were compared with the reference morphogenesis (pI) proteins with hmmsearch^46^ (hmmer.org, score ≥ 30 and *e* ≤ 0.001) for the pI-like iPFs and blastp^49^ (bit score ≥ 50) for the singleton pI protein (*Acholeplasma* phage MV-L1). These included 54,405 bacterial genomes, 1,304 archaeal genomes and 1,149 plasmid sequences. A total of 6,819 hits were detected, from which 795 corresponded to complete inovirus genomes. These included 213 circular contigs, that is, likely complete genomes, and 582 integrated prophages with canonical attachment (att) sites, that is, direct repeats of ≥10 bp in a tRNA or outside of an integrase gene. All sequences were manually inspected to verify that these were plausible inovirus genomes (see Supplementary Notes). The predicted pI proteins from the curated genomes were then added to the references to generate new improved HMM models. Using these improved models, an additional set of 639 putative pI proteins was identified. New models were built from these proteins and used in a third round of searches, which did not yield any additional genuine inovirus sequence after manual inspection.

An automatic classifier was trained on this extended inovirus genome catalogue, that is, the reference genomes and the 795 manually curated genomes, to detect putative inovirus fragments around pI-like genes, based on 10 distinctive features of inovirus genomes (Supplementary Fig. 2 and Supplementary Notes). These 795 manually curated genomes were identified from 17 host phyla (or class for Proteobacteria) and were later classified into 5 proposed families and 245 proposed subfamilies (see below ‘Gene-content-based clustering of inovirus genomes’). Three types of classifiers were tested: random forest (function randomForest from R package randomForest^50^ using 2,000 trees, other parameters left as default), random forest with conditional inference (function cforest from R package party^51^ using 2,000 trees, other parameters left as default) and a generalized linear model with lasso regularization (function glmnet from R package glmnet^52^). The efficiency of classifiers was evaluated via a tenfold cross-validation in which the input data set was partitioned into ten equal-sized subsamples, with one retained for validation and the other nine used for training through the ten possible permutations. Results were visualized as a ROC curve generated with ggplot2 (refs. ^53,54^). The importance of features in the random forest classifier was evaluated using the function ‘importance’, from the R package randomForest.

On the basis of the inflection point observed on the ROC curves, the random forest classifier was selected as the optimal method as it provided the highest true-positive rate (>92%) for false-positive rates of <1 % (Supplementary Fig. 2). This model was then used to classify all putative inovirus fragments that had not been identified as complete genomes previously, using a sliding window approach (up to 30 genes around the putative pI protein), and looking for the fragment with the maximum score in the random forest model (if >0.9). For the predicted integrated prophages, putative non-canonical att sites were next searched as direct repeats (10 bp or longer) around the fragment. Overall, 3,908 additional putative inovirus sequences were detected, including 738 prophages flanked by direct repeats.

A similar approach was used to search for inovirus sequences in 6,412 metagenome assemblies (Supplementary Table 2). Predicted proteins were compared with the 4 HMM profiles as well as to the *Acholeplasma* phage MV-L1 singleton sequence, which led to 27,037 putative pI proteins using the same thresholds as for isolate genomes. The final data set of inovirus sequences predicted from these metagenome assemblies consisted of 6,094 sequences, including 922 circular contigs, 44 prophages with canonical att sites (direct repeats of 10 bp or longer in a tRNA or next to an integrase) and 994 prophages with non-canonical att sites (direct repeats of 10 bp or longer).

### Clustering of inovirus genomes in putative species

Next, we sought to cluster these putative inovirus genomes along with the previously collected reference genomes to remove duplicated sequences and to select only one representative per species. This clustering was conducted according to the latest guidelines submitted to the International Committee on Taxonomy of Viruses (ICTV) for *Inoviridae*, that is, “95% DNA sequence identity as the criterion for demarcation of species”^55^ (https://talk.ictvonline.org/files/ictv_official_taxonomy_updates_since_the_8th_report/m/prokaryote-official/6774/download), and included our 10,295 sequences alongside the 56 reference genomes. Notably, however, predictions spanning multiple tandemly integrated inovirus prophages had to be processed separately, otherwise they could lead to clusters gathering multiple species. To detect these cases of tandem insertions, we searched for and clustered separately all predictions with multiple pI proteins, as this gene is expected to be present in single copy in inoviruses (*n* = 800 sequences).

All non-tandem sequences were first clustered incrementally with priority given to complete genomes over partial genomes as well as fragments identified in microbial genomes over fragments from metagenomes. First, circular contigs and prophages with canonical att sites identified in a microbial genome were clustered, and all other fragments were affiliated to these seed sequences. Next, unaffiliated fragments detected in microbial genomes and with non-canonical att sites (that is, simple direct repeat) were clustered together, and other fragments were affiliated to this second set of seed sequences. Finally, the remaining unaffiliated sequences detected in microbial genomes were clustered together. This allowed us to use the more ‘certain’ predictions (that is, circular sequences and prophages with identified att sites) preferentially as seeds of putative species.

A similar approach was used to cluster sequences identified from metagenomes, as well as to separately cluster putative tandem fragments, that is, those including multiple pI proteins. All the clustering and affiliation was done with a threshold of 95% ANI on 100% of alignment fraction (according to the ICTV guidelines), with sequence similarity computed using mummer^56^. Accumulation curves were calculated for 100 random ordering of input sequences using a custom perl script and plotted with ggplot2 (refs. ^53,54^).

### Clustering of predicted proteins from non-redundant inovirus sequences

Predicted proteins from the representative genome of each putative species were next clustered using the same approach as for the reference genomes. A clustering into PCs was first achieved through an all-versus-all blastp using hits with *e* ≤ 0.001 and bit score ≥ 50 or bit score ≥ 30 if both proteins are ≤70 amino acids. HMM profiles were constructed for the 5,142 PCs and these were compared all-versus-all using HHSearch, keeping hits with ≥90% probability and ≥50% coverage or ≥99% probability, ≥20% coverage and hit length of ≥100. This resulted in 4,008 protein families (iPFs).

The PCs were subsequently used for taxonomic classification of the inovirus sequences (see below), while iPFs were primarily used for functional affiliation. iPF functions were predicted based on the affiliation of iPF members against PFAM v30 (score ≥ 30), as well as manual inspection of individual iPFs using HHPred^57^.

PCs containing pI-like proteins were also further evaluated to identify potential false positives stemming from a related ATPase encoded by another type of virus or mobile genetic element (see Supplementary Notes). The criteria used to determine genuine inovirus pI-like PCs were: the PC members closest known functional domain was Zot (based on the hmmsearch against PFAM), the proteins contained one or two TMD (either N-terminal or C-terminal), at least half of the sequences encoding this PC also include other genes expected in an inovirus sequence such as replication initiation proteins, and no significant similarity could be identified to any other type of ATPase using HHpred^57^.

### Gene-content-based clustering of inovirus genomes

A bipartite network was built in which genomes and PCs (as nodes) are connected by an edge when a predicted protein from the genome is a member of the PC. This network was then used to classify inovirus sequences as done previously for dsDNA viruses^32^. PCs were used instead of iPFs as they offer a higher resolution. Sequences with two pI proteins (that is, tandem prophages) were excluded from this network-based classification as these could lead to improper connections between unrelated genomes. Singleton proteins were also excluded, and only PCs with at least 2 members were used to build the network. This network had a very low density (0.05%) reflecting the fact that most PCs were restricted to a minor fraction of the genomes. Nevertheless, this type of network can still be organized into meaningful groups through information theoretic approaches: here, sequence clusters were obtained through InfoMap, with default parameters and a two-level clustering (that is, genomes can be associated with a group and a subgroup).

A summarized representation of the network was generated by displaying each subgroup (level 2) as a node with a size proportional to the number of species in the subgroup, and drawing an edge to a PC if >50% of the subgroup sequences encode this PC, except for the larger group (‘Protoinoviridae’:Subfamily_1) where connections are drawn for PCs found in >25% of the sequences. The network was then visualized using Cytoscape^58^, with nodes from the same group (level 1) first gathered manually, and nodes allotment within group automatically generated using Prefuse-directed layout (default spring length of 200).

To evaluate the taxonomic rank to which these groups and subgroups would correspond, we calculated pairwise amino acid identity percentage of pI proteins for genomes (1) between groups and (2) within groups but between subgroups, using Sequence Demarcation Tool^59^. These were then compared with the pairwise amino acid identity calculated with the same approach for established viral groups, namely, *Caudovirales* order using the terminase large subunit (TerL) as a marker protein, *Microviridae* using the major capsid protein (VP1) as a marker protein and *Circoviridae* using the replication initiation protein (Rep) as a marker protein (see Supplementary Notes).

### Distribution of inovirus sequences by host and biome

The distribution of hosts for inovirus sequences was based on detections in IMG draft and complete genomes, that is, excluding all metagenome-derived detections but including detections in metagenome-assembled genomes (published draft genomes assembled from metagenomes). Host taxonomic classification was extracted from the IMG database. For visualization purposes, a set of 56 universal single-copy marker proteins^60,61^ was used to build phylogenetic trees for bacteria and archaea based on all available microbial genomes in IMG^23^ (genomes downloaded on 27 October 2017) and about 8,000 metagenome-assembled genomes from the Genome Taxonomy Database^62^ (downloaded on 18 October 2017). Marker proteins were identified with hmmsearch (version 3.1b2, hmmer.org) using a specific HMM for each of the markers. Genomes lacking a substantial proportion of marker proteins (>28) or which had additional copies of >3 single-copy markers were removed from the data set.

To reduce redundancy and to enable a representative taxon sampling, DNA-directed RNA polymerase β-subunit 160 kDa (COG0086) was identified using hmmsearch (hmmer 3.1b2) and the HMM of COG0086 (ref. ^63^). Protein hits were then extracted and clustered with cd-hit^45^ at 65% sequence similarity, resulting in 99 archaeal and 837 bacterial clusters. Genomes with the greatest number of different marker proteins were selected as cluster representatives. For every marker protein, alignments were built with MAFFT^64^ (v7.294b) and subsequently trimmed with BMGE (v1.12) using BLOSUM30 (ref. ^65^). Single-protein alignments were then concatenated, resulting in an alignment of 11,220 sites for the archaea and 16,562 sites for the bacteria. Maximum-likelihood phylogenies were inferred with FastTree2 (v2.1.9 SSE3, OpenMP)^66^ using the options: -spr 4 -mlacc 2 -slownni -lg.

A distribution of inovirus sequences across biomes was obtained by compiling ecosystems and sampling location of all metagenomes where at least one inovirus sequence was detected. This information was extracted from the GOLD database^67^, and the map was generated using the BaseMap functions from the matplotlib python library^68^.

### Estimation of inovirus prevalence and co-infection patterns

Prevalence and co-infection patterns were evaluated from the set of sequences identified in complete and draft microbial genomes from the IMG database, that is, excluding detections from metagenome assemblies. To control for the presence of near-identical genomes in the database, prevalence and co-infection frequencies were calculated after clustering host genomes based on pairwise ANI (cut-offs: 95% nucleotide identity on 95% alignment fraction). Prevalence was calculated at the host genus rank as the number of genomes with one or more inovirus sequence detected. Co-occurrence of inoviruses was evaluated based on the detections of distinct species in single-host genomes. Finally, we evaluated the rate of bacteria and archaea co-infected by an inovirus and a member of the *Caudovirales* order, the group of dsDNA viruses including most of the characterized bacteriophages (both lytic and temperate) as well as several archaeoviruses. To identify *Caudovirales* infections, we used the gene encoding the terminase large subunit as a marker gene, and searched the same genomes from the IMG database for hits to the PFAM domains terminase_1, terminase_3, terminase_6 and terminase_GpA (hmmsearch, score ≥ 30).

### Phylogenetic trees of inovirus sequences

Phylogenies of inovirus sequences were based on multiple alignment of pI protein sequences. To obtain informative multiple alignments, an all-versus-all blastp^49^ of all pI proteins was computed and used to identify the nearest neighbours of sequences of interests. For sequences detected in archaeal genomes, an additional 10 most closely related sequences with *e* ≤ 0.001, bit score ≥ 50 and a blast hit covering ≥50% of the query sequence were recruited for each archaea-associated sequence to help populate the tree. A similar approach was used for the tree based on the integrase genes from archaea-associated inoviruses: the protein sequences for the three integrase genes were compared with the NCBI nr database with blastp^49^ (bit score ≥ 50, *e* ≤ 0.001) to gather their closest neighbours across archaeal and bacterial genomes.

Resulting data sets were first filtered for partial sequences as follows: the average sequence length was calculated excluding the top and bottom 10%, and all sequences shorter than half of this average were excluded. These protein sequences were next aligned with MUSCLE (v3.8.1551)^43^, automatically trimmed with trimAL (v1.4.rev15)^69^ (option gappyout), and trees were constructed using IQ-TREE (v1.5.5) with an automatic detection of optimal model^70^ and displayed using iToL^71^. The optimal substitution model, selected based on the Bayesian information criterion, was VT + F + R5 for the the pI phylogeny of archaeal inoviruses, and LG + R4 for the integrase phylogeny of archaeal inoviruses. Annotated trees are available at http://itol.embl.de/shared/Siroux (project ‘Inovirus’).

### Functional affiliation of iPFs

An automatic functional affiliation of all iPFs was generated by compiling the annotation of all members based on a comparison to PFAM (data extracted from the IMG). To refine these annotations for functions of interest, namely, replication initiation proteins, integration proteins, DNA methylases and toxin–antitoxin systems, individual iPF alignments were submitted to the HHPred website^57^, and the alignments were visually inspected for conserved residues and/or motifs (Supplementary Table 5, motifs extracted from refs. ^72,73^ and the PFAM database v30 (ref. ^74^)).

To identify toxin–antitoxin protein partners, all inovirus sequences were screened for co-occurring genes including an iPF annotated as toxin and/or antitoxin, and the list of putative pairs was next manually curated (Supplementary Table 5). This enabled the identification of putative antitoxin proteins detected as conserved uncharacterized iPF frequently observed next to a predicted toxin iPF.

Finally, putative structural proteins and DNA-interacting proteins were specifically searched for. Putative structural proteins were predicted as described above for the isolate reference genomes, that is, as sequences of 30–90 amino acids, after in silico removal of signal peptide, if detected, and displaying 1 or 2 TMD. For the most abundant iPFs predicted as major coat proteins, the secondary structure was predicted with Phyre2 (ref. ^75^). For DNA-interacting proteins, PFAM annotations were screened for HTH, RHH, Zn-binding and Zn-ribbon domains. In addition, HHsearch was used to compare the iPFs to 3 conserved HTH domains from the SMART database^76^: Bac_DnaA_C, HTH_DTXR and HTH_XRE (probability ≥ 90).

### CRISPR spacer matches and CRISPR–Cas systems identification

All inovirus sequences were compared with the IMG CRISPR spacer database with blastn, using options adapted for short sequences (-task blastn-short -evalue 1 -word_size 7 -gapopen 10 -gapextend 2 -penalty −1 -dust no). Only cases with zero or one mismatch were further considered. Next, the genome context of these spacers was explored to identify the ones with a clear associated CRISPR–Cas system and to affiliate these systems to the different types described. Only spacers for which a *cas* gene could be identified in a region of ±10 kb were retained. The CRISPR–Cas system affiliation was based on the set of *cas* genes identified around the spacer and performed following the guidelines from ref. ^77^.

For host genomes with a self-targeting spacer, additional (that is, non-inovirus) prophages were detected using VirSorter^20^. The number of distinct prophages was also estimated using the detection of large terminase subunits (hmmsearch against PFAM database, score ≥ 30). Putative Acr and anti-CRISPR-associated (Aca) proteins were first detected through similarity to previously described Acr systems^38^ (blastp, *e* ≤ 0.001 and score ≥ 50). Putative Acr and Aca proteins were identified by searching for HTH-domain-containing proteins identified based on HTH domains in the SMART database (see above) in inovirus sequences displaying a match to a CRISPR spacer extracted from the same host genome.

### Microscopy and PCR investigation of a predicted provirus in *M.* *profundi* MobM

*M.* *profundi* strain MobM cells were grown in anaerobic DSMZ medium 479 at 37 °C with 5 mM methanol added as a methanogenic substrate instead of trimethylamine^78^. After 35 h of growth, anaerobic mitomycin C was added to the culture at a final concentration of 1.0 μg ml^−1^ to induce the provirus. Samples were collected before and 4 h after induction and were filtered with 0.22-μm pore size polyethersulfone filters (Millipore, Fisher Scientific) to obtain a ‘cellular’ (≥0.22 μm) and a ‘viral’ (<0.22 µm) fraction.

The four types of samples (with or without induction, cellular and viral fractions) were prepared and imaged at the Molecular and Cellular Imaging Center, Ohio State University, Wooster, OH, USA. An equal volume of 2× fixative (6% glutaraldehyde and 2% paraformaldehyde in 0.1 M potassium phosphate buffer pH 7.2) was added directly to the culture post-induction. Of the medium, 30 μl was applied to a formovar and carbon-coated copper grid for 5 min, blotted and then stained with 2% uranyl acetate for 1 min. Samples were examined with a Hitachi H7500 electron microscope and imaged with the SIA-L12C (16 megapixels) digital camera.

PCRs were initially run for induced and non-induced samples on both size fractions with three pairs of primers: one internal to the predicted provirus (B primers), one spanning the insertion site (P primers) and one spanning the junction of the predicted excised circular genome (C primers). The reactions were conducted for 35 cycles with denaturation, annealing and extension cycles of 0.5, 0.5 and 1.0 min at 95.0, 52.0 and 72.0 °C, respectively. For C primers, numerous nonspecific amplification products were obtained with these conditions, and another set of PCRs was conducted with higher annealing temperatures of 56.5 °C and 57.5 °C, both in triplicates. The PCR product was then cleaned to remove polymerase, free dNTPs and primers (Zymo Research) and subsequently used as templates for Sanger sequencing. The resulting chromatograms were analysed using the R^54^ packages sangerseqR^79^, sangeranalyseR^80^ and readr^81^. The extracted primary sequences were aligned to the MobM genome using blastn^49^ and MUSCLE^43^, and the alignment was visualized with Jalview^82^.

### Experimental characterization of hypothetical proteins from self-targeted *Pseudomonas* inoviruses

Hypothetical proteins predicted on inovirus prophages, which were (1) found in *Pseudomonas* genomes, (2) predicted to be targeted by at least one CRISPR spacer from the same genome, and (3) for which no *acr* locus could be identified anywhere else in the same genome, were selected for further functional characterization. The ten candidate genes were first codon optimized for expression in *Pseudomonas* using an empirically derived codon usage table. Codon optimization and vendor defined synthesis constraints removal were performed using BOOST^83^. Synthetic DNA were obtained from Thermo Fisher Scientific and cloned in between the SacI and PstI sites of an *Escherichia*–*Pseudomonas* broad host range expression vector, pHERD30T^84^. All gene constructs were sequence-verified before testing.

*P.* *aeruginosa* strains (PAO1::pLac I-C CRISPR–Cas, PA14 and 4386) were cultured on LB agar or liquid media at 37 °C. The pHERD30T plasmids were electroporated into *P.* *aeruginosa* strains, and LB was supplemented with 50 µg ml^−1^ gentamicin to maintain the pHERD30T plasmid. Phages DMS3m, JBD30, D3, 14–1, Luz7 and KMV were amplified on PAO1, and phage JBD44a was amplified on PA14. All phages were stored in SM buffer at 4 °C in the presence of chloroform.

For phage titring, a bacterial lawn was first generated by spreading 6 ml of top agar seeded with 200 µl host bacteria on a LB agar plate supplemented with 10 mM MgSO_4_, 50 µg ml^−1^ gentamicin and 0.1% arabinose. The I-C *cas* genes in strain PAO1 were induced with 1 mM isopropyl-β-d-1-thiogalactopyranoside. Three microlitres of phage serially diluted in SM buffer was then spotted onto the lawn and incubated at 37 °C for 16 h. Growth rates were similar between cells transformed with an empty vector and cells transformed with a vector including a candidate gene, except for the two cases where no growth was observed after transformation (see Supplementary Notes).

### Experimental confirmation of self-targeting lethality and *trans*-acting Acr activity from a co-infecting phage in a *P.* *aeruginosa* model

The effect of CRISPR targeting of an integrated inovirus prophage was assessed in the *P.* *aeruginosa* strain PA14, which naturally encodes an intact Pf1 inovirus prophage, and for which both natural CRISPR arrays were deleted (strain PA14 ∆CRISPR1/∆CRISPR2 (Pf1)). Host cells were transformed with plasmids encoding CRISPR spacers either targeting the Pf1 coat gene or without a target in the host genome. To generate these plasmids, complementary single-stranded oligos (IDT) were annealed and ligated into a linearized derivative of shuttle vector pHERD30T bearing I-F direct repeats in the multiple cloning site downstream of the pBAD promoter. PA14 lysogens were electroporated with 100 ng plasmid DNA, allowed to recover for 1 h in LB at 37 °C and plated on LB agar plates supplemented with 50 μg ml^−1^ gentamicin and 0.1% arabinose. Colonies were enumerated after growth for 14 h at 37 °C. Transformation efficiency (TE) was calculated as colonies per microgram DNA, and the percentage TE was calculated by normalizing the TE of the CRISPR RNA-expressing plasmids to the TE of an empty vector.

To evaluate the effect of an *acr* locus from a co-infecting prophage on self-targeted inoviruses, strain PA14 ∆CRISPR1/∆CRISPR2 (Pf1) was lysogenized with phage DMS3m_acrIF1_ by streaking out cells from a solid plate infection and screening for colonies resistant to superinfection by DMS3m_acrIF1_. Lysogeny was confirmed by prophage induction. The same plasmid transformation approach was then used to assess the effect of inovirus self-targeting on host cell viability.

### Quantification and statistical analysis

Sequence similarity searches were conducted with thresholds of *E-value* ≤ 0.001 and bit score ≥ 30 or 50, the former being used mainly for short proteins. The different classifiers (random forest, conditional random forest and generalized linear model) used to identify inovirus sequences were evaluated using a tenfold cross-validation approach. For all boxplots, the lower and upper hinges correspond to the first and third quartiles, respectively, and the whiskers extend no further than ±1.5 times the interquartile range.

### Reporting Summary

Further information on research design is available in the Nature Research Reporting Summary linked to this article.

## Supplementary information

Supplementary Information Supplementary Notes, Supplementary Figs. 1–12, Supplementary Table legends and Supplementary References.

Reporting Summary

Supplementary Table 1 List and characteristics of reference inovirus genomes used in this study.

Supplementary Table 2 List of genomes and metagenomes mined.

Supplementary Table 3 Classification of inovirus sequences into species, proposed families and proposed subfamilies.

Supplementary Table 4 Additional indication of inovirus infection for 20 phylum-level putative host groups.

Supplementary Table 5 Functional annotation of protein families (iPFs).

Supplementary Table 6 List of matches between inovirus sequences and IMG CRISPR spacer database.

## Data Availability

The following files are available at https://genome.jgi.doe.gov/portal/Inovirus/Inovirus.home.html: Gb_files_inoviruses.zip: GenBank files of all representative genomes for each inovirus species; Ref_PCs_inoviruses.zip: PCs from the references (raw fasta, alignment fasta and hmm profile); iPFs_inoviruses.zip: protein families from the extended inovirus data set (raw fasta, alignment fasta and hmm profile); MobM_C_primer_amplicon.fasta: multiple sequence alignment of the C primer products with the *Methanolobus* MobM genome (NZ_FOUJ01000007), confirming that C primer products span the junction of the excised genome. Accession numbers of all inovirus sequences used as reference are listed in Supplementary Table 1. Accession numbers of all genomes and metagenomes mined, including detailed information for each (meta)genome in which some inovirus sequences were detected are available in Supplementary Table 2. Finally, the list of all inovirus genome accession numbers, along with taxonomic and environmental distribution information, is provided in Supplementary Table 3.

## Code availability

The set of scripts and models used to detect inovirus sequences is available at https://bitbucket.org/srouxjgi/inovirus/src/master/Inovirus_detector/.

## References

[1] Rakonjac J, Bennett NJ, Spagnuolo J, Gagic D, Russel M (2011). Filamentous bacteriophage: biology, phage display and nanotechnology applications. Curr. Issues Mol. Biol..

[2] Fauquet CM (2006). The diversity of single stranded DNA. Virus Biodivers..

[3] Marvin DA, Symmons MF, Straus SK (2014). Structure and assembly of filamentous bacteriophages. Prog. Biophys. Mol. Biol..

[4] Bradbury ARM, Marks JD (2004). Antibodies from phage antibody libraries. J. Immunol. Methods.

[5] Nam KT (2008). Stamped microbattery electrodes based on self-assembled M13 viruses. Proc. Natl Acad. Sci. USA.

[6] Ju Z, Sun W (2017). Drug delivery vectors based on filamentous bacteriophages and phage-mimetic nanoparticles. Drug Deliv..

[7] Henry KA, Arbabi-Ghahroudi M, Scott JK (2015). Beyond phage display: non-traditional applications of the filamentous bacteriophage as a vaccine carrier, therapeutic biologic, and bioconjugation scaffold. Front. Microbiol..

[8] Ilyina TS (2015). Filamentous bacteriophages and their role in the virulence and evolution of pathogenic bacteria. Mol. Genet. Microbiol. Virol..

[9] Shapiro JW, Turner PE (2018). Evolution of mutualism from parasitism in experimental virus populations. Evolution.

[10] Sweere JM (2019). Bacteriophage trigger anti-viral immunity and prevent clearance of bacterial infection. Science.

[11] Waldor MK, Mekalanos JJ (1996). Lysogenic conversion by a filamentous phage encoding cholera toxin. Science.

[12] Faruque SM, Mekalanos JJ (2003). Pathogenicity islands and phages in *Vibrio cholerae* evolution. Trends Microbiol..

[13] Bille E (2017). A virulence-associated filamentous bacteriophage of *Neisseria meningitidis* increases host-cell colonisation. PLoS Pathog..

[14] Rice SA (2009). The biofilm life cycle and virulence of *Pseudomonas aeruginosa* are dependent on a filamentous prophage. ISME J..

[15] Rakonjac, J. Filamentous bacteriophages: biology and applications. *eLS*10.1002/9780470015902.a0000777 (2012).

[16] Varani AM, Monteiro-Vitorello CB, Nakaya HI, Van Sluys M-A (2013). The role of prophage in plant-pathogenic bacteria. Annu. Rev. Phytopathol..

[17] Mai-Prochnow A (2015). ‘Big things in small packages: the genetics of filamentous phage and effects on fitness of their host’. FEMS Microbiol. Rev..

[18] Páez-Espino D (2016). Uncovering Earth’s virome. Nature.

[19] Páez-Espino D, Pavlopoulos GA, Ivanova NN, Kyrpides NC (2017). Nontargeted virus sequence discovery pipeline and virus clustering for metagenomic data. Nat. Protoc..

[20] Roux S, Enault F, Hurwitz BL, Sullivan MB (2015). VirSorter: mining viral signal from microbial genomic data. PeerJ.

[21] Brum JR, Sullivan MB (2015). Rising to the challenge: accelerated pace of discovery transforms marine virology. Nat. Rev. Microbiol..

[22] Vega Thurber RV (2009). Laboratory procedures to generate viral metagenomes. Nat. Protoc..

[23] Chen IMA (2017). IMG/M: integrated genome and metagenome comparative data analysis system. Nucleic Acids Res..

[24] Kimura, M., Wang, G., Nakayama, N. & Asakawa, S. in *Biocommunication in Soil Microorganisms* (ed. Witzany, G.) 189–213 (Springer, 2011).

[25] Kim AY, Blaschek HP (1991). Isolation and characterization of a filamentous virus-like particle from *Clostridium acetobutylicum* NCIB-6444. J. Bacteriol..

[26] Iranzo J, Koonin EV, Prangishvili D, Krupovic M (2016). Bipartite network analysis of the archaeal virosphere: evolutionary connections between viruses and capsid-less mobile elements. J. Virol..

[27] Prangishvili D, Bamford DH, Forterre P, Iranzo J (2017). The enigmatic archaeal virosphere. Nat. Rev. Microbiol..

[28] Krupovic M, Cvirkaite-Krupovic V, Iranzo J, Prangishvili D, Koonin EV (2018). Viruses of archaea: structural, functional, environmental and evolutionary genomics. Virus Res..

[29] Garushyants SK, Kazanov MD, Gelfand MS (2015). Horizontal gene transfer and genome evolution in *Methanosarcina*. BMC Evol. Biol..

[30] Mavrich TN, Hatfull GF (2017). Bacteriophage evolution differs by host, lifestyle and genome. Nat. Microbiol..

[31] Krupovic M, Prangishvili D, Hendrix RW, Bamford DH (2011). Genomics of bacterial and archaeal viruses: dynamics within the prokaryotic virosphere. Microbiol. Mol. Biol. Rev..

[32] Iranzo J, Krupovic M, Koonin EV (2016). The double-stranded DNA virosphere as a modular hierarchical network of gene sharing. mBio.

[33] Rosvall M, Bergstrom CT (2011). Multilevel compression of random walks on networks reveals hierarchical organization in large integrated systems. PLoS ONE.

[34] Wolf YI (2018). Origins and evolution of the global RNA virome. mBio.

[35] Koonin EV, Dolja VV, Krupovic M (2015). Origins and evolution of viruses of eukaryotes: the ultimate modularity. Virology.

[36] Song S, Wood TK (2018). Post-segregational killing and phage inhibition are not mediated by cell death through toxin/antitoxin systems. Front. Microbiol..

[37] Marraffini LA (2015). CRISPR–Cas immunity in prokaryotes. Nature.

[38] Borges AL, Davidson AR, Bondy-Denomy J (2017). The discovery, mechanisms, and evolutionary impact of anti-CRISPRs. Annu. Rev. Virol..

[39] Díaz-Muñoz SL, Sanjuán R, West S (2017). Sociovirology: conflict, cooperation, and communication among viruses. Cell Host Microbe.

[40] O’Leary NA (2016). Reference sequence (RefSeq) database at NCBI: current status, taxonomic expansion, and functional annotation. Nucleic Acids Res..

[41] Brister JR, Ako-Adjei D, Bao Y, Blinkova O (2015). NCBI viral genomes resource. Nucleic Acids Res..

[42] Delcher AL, Bratke KA, Powers EC, Salzberg SL (2007). Identifying bacterial genes and endosymbiont DNA with Glimmer. Bioinformatics.

[43] Edgar RC (2004). MUSCLE: a multiple sequence alignment method with reduced time and space complexity. BMC Bioinformatics.

[44] Remmert M, Biegert A, Hauser A, Söding J (2011). HHblits: lightning-fast iterative protein sequence searching by HMM-HMM alignment. Nat. Methods.

[45] Fu L, Niu B, Zhu Z, Wu S, Li W (2012). CD-HIT: accelerated for clustering the next-generation sequencing data. Bioinformatics.

[46] Eddy SR (2011). Accelerated profile HMM searches. PLoS Comput. Biol..

[47] Petersen TN, Brunak S, Von Heijne G, Nielsen H (2011). SignalP 4.0: discriminating signal peptides from transmembrane regions. Nat. Methods.

[48] Krogh A, Larsson B, von Heijne G, Sonnhammer ELL (2001). Predicting transmembrane protein topology with a hidden Markov model: application to complete genomes. J. Mol. Biol..

[49] Camacho C (2009). BLAST+: architecture and applications. BMC Bioinformatics.

[50] Liaw A, Wiener M (2002). Classification and regression by randomForest. R News.

[51] Strobl C, Boulesteix AL, Kneib T, Augustin T, Zeileis A (2008). Conditional variable importance for random forests. BMC Bioinformatics.

[52] Simon N, Friedman J, Hastie T, Tibshirani R (2011). Regularization paths for Cox’s proportional hazards model via coordinate descent. J. Stat. Softw..

[53] Wickham, H. *ggplot2: Elegant Graphics for Data Analysis* (Springer, 2016).

[54] R Core Team *R: A Language and Environment for Statistical Computing* (R Foundation for Statistical Computing, 2018).

[55] Adriaenssens EM, Krupovic M, Knezevic P (2017). Taxonomy of prokaryotic viruses: 2016 update from the ICTV bacterial and archaeal viruses subcommittee. Arch. Virol..

[56] Kurtz S (2004). Versatile and open software for comparing large genomes. Genome Biol..

[57] Alva V, Nam S-Z, Söding J, Lupas AN (2016). The MPI bioinformatics toolkit as an integrative platform for advanced protein sequence and structure analysis. Nucleic Acids Res..

[58] Demchak B (2014). Cytoscape: the network visualization tool for GenomeSpace workflows. F1000Res..

[59] Muhire BM, Varsani A, Martin DP (2014). SDT: a virus classification tool based on pairwise sequence alignment and identity calculation. PLoS ONE.

[60] Eloe-Fadrosh EA (2016). Global metagenomic survey reveals a new bacterial candidate phylum in geothermal springs. Nat. Commun..

[61] Yu FB (2017). Microfluidic-based mini-metagenomics enables discovery of novel microbial lineages from complex environmental samples. eLife.

[62] Parks DH (2017). Recovery of nearly 8,000 metagenome-assembled genomes substantially expands the tree of life. Nat. Microbiol..

[63] Tatusov RL (2000). The COG database: a tool for genome-scale analysis of protein functions and evolution. Nucleic Acids Res..

[64] Katoh K, Standley DM (2013). MAFFT multiple sequence alignment software version 7: improvements in performance and usability. Mol. Biol. Evol..

[65] Criscuolo A, Gribaldo S (2010). BMGE (block mapping and gathering with entropy): a new software for selection of phylogenetic informative regions from multiple sequence alignments. BMC Evol. Biol..

[66] Price MN, Dehal PS, Arkin AP (2010). FastTree 2—approximately maximum-likelihood trees for large alignments. PLoS ONE.

[67] Mukherjee S (2017). Genomes Online Database (GOLD) v.6: data updates and feature enhancements. Nucleic Acids Res..

[68] Hunter JD (2007). Matplotlib: a 2D graphics environment. Comput. Sci. Eng..

[69] Capella-Gutiérrez S, Silla-Martínez JM, Gabaldón T (2009). trimAl: a tool for automated alignment trimming in large-scale phylogenetic analyses. Bioinformatics.

[70] Nguyen LT, Schmidt HA, Von Haeseler A, Minh BQ (2015). IQ-TREE: a fast and effective stochastic algorithm for estimating maximum-likelihood phylogenies. Mol. Biol. Evol..

[71] Letunic I, Bork P (2016). Interactive Tree of Life (iTOL) v3: an online tool for the display and annotation of phylogenetic and other trees. Nucleic Acids Res..

[72] Krupovic M (2013). Networks of evolutionary interactions underlying the polyphyletic origin of ssDNA viruses. Curr. Opin. Virol..

[73] Carr SB, Phillips SEV, Thomas CD (2016). Structures of replication initiation proteins from staphylococcal antibiotic resistance plasmids reveal protein asymmetry and flexibility are necessary for replication. Nucleic Acids Res..

[74] Finn RD (2016). The Pfam protein families database: towards a more sustainable future. Nucleic Acids Res..

[75] Kelley LA, Mezulis S, Yates C, Wass M, Sternberg M (2015). The Phyre2 web portal for protein modelling, prediction, and analysis. Nat. Protoc..

[76] Letunic I (2004). SMART 4.0: towards genomic data integration. Nucleic Acids Res..

[77] Makarova KS (2015). An updated evolutionary classification of CRISPR–Cas systems. Nat. Rev. Microbiol..

[78] Mochimaru H (2009). *Methanolobus profundi* sp. nov., a methylotrophic methanogen isolated from deep subsurface sediments in a natural gas field. Int. J. Syst. Evol. Microbiol..

[79] Hill JT (2014). Poly peak parser: method and software for identification of unknown indels using sanger sequencing of polymerase chain reaction products. Dev. Dyn..

[80] Lanfear, R. sangeranalyseR: a suite of functions for the analysis of Sanger sequence data in R v.1.20.0 (2015).

[81] Wickham, H., Hester, J. & Francois, R. readr: read rectangular text data v.1.3.1 (2017).

[82] Waterhouse AM, Procter JB, Martin DMA, Clamp M, Barton GJ (2009). Jalview version 2—a multiple sequence alignment editor and analysis workbench. Bioinformatics.

[83] Oberortner E, Cheng JF, Hillson NJ, Deutsch S (2017). Streamlining the design-to-build transition with build-optimization software tools. ACS Synth. Biol..

[84] Qiu D, Damron FH, Mima T, Schweizer HP, Yu HD (2008). PBAD-based shuttle vectors for functional analysis of toxic and highly regulated genes in *Pseudomonas* and *Burkholderia* spp. and other bacteria. Appl. Environ. Microbiol..

