# Author Correction: Cryptic inoviruses revealed as pervasive in bacteria and archaea across Earth’s biomes

**DOI:** 10.1038/s41564-020-0681-5

**Published:** 2020-02-11

**Authors:** Simon Roux, Mart Krupovic, Rebecca A. Daly, Adair L. Borges, Stephen Nayfach, Frederik Schulz, Allison Sharrar, Paula B. Matheus Carnevali, Jan-Fang Cheng, Natalia N. Ivanova, Joseph Bondy-Denomy, Kelly C. Wrighton, Tanja Woyke, Axel Visel, Nikos C. Kyrpides, Emiley A. Eloe-Fadrosh

**Affiliations:** 1 DOE Joint Genome Institute, Walnut Creek, CA USA; 2 Department of Microbiology, Institut Pasteur, Paris, France; 3 Department of Soil and Crop Sciences, Colorado State University, Fort Collins, CO USA; 4 Department of Microbiology and Immunology, University of California, San Francisco, San Francisco, CA USA; 5 Department of Earth & Planetary Sciences, University of California, Berkeley, Berkeley, CA USA; 6 Quantitative Biosciences Institute, University of California, San Francisco, San Francisco, CA USA

**Keywords:** Archaea, Bacteriophages, Environmental microbiology, Phage biology, Metagenomics

Correction to: *Nature Microbiology* 10.1038/s41564-019-0510-x, published online 22 July 2019.

In the version of this Article originally published, in the Data availability section, the link ‘https://genome.jgi.doe.gov/portal/PhyloTag/PhyloTag.home.html’ was incorrect; it should have been ‘https://genome.jgi.doe.gov/portal/Inovirus/Inovirus.home.html’. This has now been corrected.

