# Supplementary material for: Cryptic inoviruses revealed as pervasive in bacteria and archaea across Earth’s biomes
Source: Nat Microbiol. 2019 Jul 22;4(11):1895–906. doi: 10.1038/s41564-019-0510-x (PMC6813254; doi:10.1038/s41564-019-0510-x)
Supplement: Supplementary file 1 — Supplementary Notes, Supplementary Figs. 1–12, Supplementary Table legends and Supplementary References. [file 41564_2019_510_MOESM1_ESM.pdf]

nature microbiology

SUPPLEMENTARY INFORMATION

https://doi.org/10.1038/s41564-019-0510-x

In the format provided by the authors and unedited.

Cryptic inoviruses revealed as pervasive in bacteria and archaea across Earth's biomes

Simon Roux[1*], Mart Krupovic[2], Rebecca A. Daly[3], Adair L. Borges[4], Stephen Nayfach[1], Frederik Schulz[1], Allison Sharrar[5], Paula B. Matheus Carnevali[5], Jan-Fang Cheng[1], Natalia N. Ivanova[1], Joseph Bondy-Denomy[4,6], Kelly C. Wrighton[3], Tanja Woyke[1], Axel Visel[1], Nikos C. Kyrpides[1] and Emiley A. Eloe-Fadrosh[1*]

[1]DOE Joint Genome Institute, Walnut Creek, CA, USA. [2]Department of Microbiology, Institut Pasteur, Paris, France. [3]Department of Soil and Crop Sciences, Colorado State University, Fort Collins, CO, USA. [4]Department of Microbiology and Immunology, University of California, San Francisco, San Francisco, CA, USA. [5]Department of Earth & Planetary Sciences, University of California, Berkeley, Berkeley, CA, USA. [6]Quantitative Biosciences Institute, University of California, San Francisco, San Francisco, CA, USA. *e-mail: sroux@lbl.gov; eaeloefadrosh@lbl.gov

nature microbiology

SUPPLEMENTARY INFORMATION

https://doi.org/10.1038/s41564-019-0510-x

In the format provided by the authors and unedited.

Cryptic inoviruses revealed as pervasive in bacteria and archaea across Earth's biomes

Simon Roux[1*], Mart Krupovic[2], Rebecca A. Daly[3], Adair L. Borges[4], Stephen Nayfach[1], Frederik Schulz[1], Allison Sharrar[5], Paula B. Matheus Carnevali[5], Jan-Fang Cheng[1], Natalia N. Ivanova[1], Joseph Bondy-Denomy[4,6], Kelly C. Wrighton[3], Tanja Woyke[1], Axel Visel[1], Nikos C. Kyrpides[1] and Emiley A. Eloe-Fadrosh[1*]

[1]DOE Joint Genome Institute, Walnut Creek, CA, USA. [2]Department of Microbiology, Institut Pasteur, Paris, France. [3]Department of Soil and Crop Sciences, Colorado State University, Fort Collins, CO, USA. [4]Department of Microbiology and Immunology, University of California, San Francisco, San Francisco, CA, USA. [5]Department of Earth & Planetary Sciences, University of California, Berkeley, Berkeley, CA, USA. [6]Quantitative Biosciences Institute, University of California, San Francisco, San Francisco, CA, USA. *e-mail: sroux@lbl.gov; eaeloefadrosh@lbl.gov

Supplementary Notes

Identification of putative marker genes and characteristic features for inovirus detection

Genome sequences and predicted proteins from 56 reference *Inoviridae* genomes (Supplementary Table 1) were gathered, and their predicted proteins were grouped into protein families using (i) all-vs-all blast and InfoMap[1] to define protein clusters, and (ii) HHSearch[2] to combine these clusters into larger protein families (see Methods). A bipartite network was then built using genomes and protein families as nodes, and connecting genomes to protein families when at least one protein affiliated to this family was encoded in the genome. The topology of this network was found to correctly recapitulate the known *Inoviridae* taxonomy, as well as their known host range (Supplementary Fig. 1). This network was thus used to identify putative core genes that could be used as marker to search for inovirus sequences. These core genes would appear in the network as protein family nodes connecting to a maximum of genomes (Supplementary Fig. 1).

No protein family was universally detected in all the reference genomes, and only morphogenesis proteins (pI) were good candidates for a marker gene: these proteins were split into 3 families only, except for the pI protein from Acholeplasma virus MV-L1 which was a singleton. Two of these protein families included sequences currently annotated as pI and displayed significant hits to the Zot PFAM domain (the only PFAM domain including pI-like proteins), while the third was identified as a pI-like protein based on its unique presence in all *Vespertilliovirus* genomes, its size being consistent with known pI proteins, and a low similarity to the Zot PFAM domain (hhsearch, score $\geq$ 15 and E-value $\geq$ 0.004), while no other proteins in these genomes displayed any similarity to Zot. Eventually, the complete set of marker included these 3 protein families, the PFAM Zot domain, as well as the putative pI from Acholeplasma phage MV-L1. For each family, HMM profiles were generated as follows: sequences were first clustered at 90% AAI with cd-hit[3], then aligned with muscle[4], and the hmm profile built with hmmbuild[5].

Canonical *Inoviridae* major coat proteins could be detected based on their length (30 to 90 aa, Supplementary Table 1), and the presence of a single transmembrane domain (TMD). A signal peptide was detected in most of these proteins (21 of 30) and the corresponding sequences had to be matured in silico (i.e. the signal peptide removed) to recover the expected size and single TMD. About half of minor coat proteins (29 of 49) could also be identified using the same features, but the remaining minor coat were either shorter (e.g. NP_039618) or longer (e.g. YP_002925193). Notably, a good major coat candidate, i.e. a protein of 30 to 90 aa and with 1 TMD, was detected in every *Inoviridae* genome, even the ones for which no protein was annotated as a major coat (Supplementary Table 1).

Design of an automatic classifier to detect inovirus sequences

To automatically detect inovirus genomes, we first searched for inovirus sequences to add to the 56 genomes database, in order to gather a positive dataset large enough for training an automatic classifier. To that end, we used the set of pI HMM profiles previously described (see

above) to search 56,868 bacterial and archaeal genomes publicly available in the IMG database, which yielded 6,819 hits (hmmsearch[5], score ≥ 30 and E-value ≤ 0.001). The genomic context of these pI-like genes was then examined in a window of 20 genes in 5’ and 3’ by (i) gathering the PFAM annotation of the genes in these regions from IMG, (ii) affiliating these genes to the previously generated reference *Inoviridae* protein families (hmmsearch[5], score ≥ 30 and E-value ≤ 0.001), and (iii) predicting putative *Inoviridae* coat proteins based on protein size, presence of a signal peptide, and single TMD (see above). From these annotations, putative complete inovirus genomes were identified by extending the prediction around the initial pI protein in 5’ and 3’ until reaching a protein affiliated to a PFAM domain never encountered in a reference *Inoviridae* genome (i.e. “unexpected PFAM affiliation”), and then assessing if the corresponding prediction either (i) spanned an entire circular contig with an expected inovirus genome size (i.e. 5-20kb), or (ii) included putative canonical attachment (att) site, i.e. direct repeats of 10bp or longer that could be identified in a tRNA gene or directly outside of an integrase gene[6]. A total of 795 putative inovirus genomes were detected: 213 as circular contigs, and 582 as integrated prophages. Their predicted pI proteins were added to the references to generate improved HMM models, and another round of search of the same datasets was conducted, adding an additional 10 putative genomes (3 circular, 7 prophages with canonical att site). The gene content of these genomes was next manually inspected to verify that these were consistent with known *Inoviridae*, and edge cases were excluded.

Next, the *Inoviridae* reference isolates and these 805 manually curated sequences were gathered as a positive set to train an automatic classifier, in order to be able to automatically evaluate a putative inovirus genome detected based on the presence of a pI-like gene. A negative set was generated by taking random fragments in genomes where an inovirus sequence was detected (n=1,000), as well as genome fragments around pI proteins manually identified as false positives, i.e. not inovirus sequences (n=1,000), with a fragment length following the length distribution of the complete inovirus genomes in both cases. This was done to ensure that the model is trained on negative cases representing both typical genome fragments from inovirus hosts, as well as typical genome context for pI-like proteins which are not associated with inovirus prophages.

A set of genome features was identified that could be used to identify genuine inoviruses (see examples in Supplementary Fig. 2). These include (i) fragment length, (ii) number of genes in the fragment, (iii) number of proteins with a hit to a pI protein family, (iv) number of genes with a significant hit to inovirus capsid PFAM domains, (v) number of genes predicted as putative inovirus coat proteins, (vi) number of genes with a significant hit to reference *Inoviridae* protein families, (vii) number of genes without an unexpected PFAM affiliation, (viii) percentage of genes in the fragment without an unexpected PFAM affiliation, (ix) median gene length in the fragment, and (x) first decile of gene length in the fragment. “Expected” affiliations were based on the affiliation of known inovirus proteins to PFAM domains and to their associated keywords (“DUF”, “HTH”, “DNA”, “repeat”, “toxin”, and “regul”), while “Unexpected” PFAM domains are the rest of the PFAM database.

Based on the training sets, a random forest classifier was found to be the most efficient at discriminating inoviruses from background host genome (compared to random forest with conditional inference and generalized linear model with lasso regularization), and achieved (at the selected threshold of score ≥ 0.9) 92.5% recall (percentage of "true" inoviruses correctly predicted as inoviruses), 99.9% specificity (percentage of "true" non-inoviruses sequences correctly predicted as non-inoviruses), and 99.8% precision (percentage of "true" inoviruses within sequences predicted as inoviruses). This model and threshold combination was chosen because it provided the maximum recall at the low false discovery rate of 0.2% (Supplementary Fig. 2). This approach can thus be used in place of the manual curation step to evaluate genome regions surrounding putative pI proteins, and systematically detect inovirus sequences with high accuracy.

Identification of non-inovirus ATPases among putative pI proteins

When detecting inoviruses using pI-like proteins, the presence of an ATPase domain in these proteins can lead to false positive detections. Our automatic classifier is able to identify most of these non-inovirus ATPases based on genome context, as illustrated by the large number of hits for which no inovirus genome was predicted (gray sections of the pie charts in Fig. 1D). However some false positives may remain, for instance due to another virus or mobile genetic element with atypical genes and/or short genes encoding a related ATPase. To identify and remove these sequences, we explored the protein clusters (PCs) computed from the complete set of genes from all inovirus species (both known and newly detected), and examined the ones including at least one protein initially identified as pI at the first detection step, i.e. used as a starting point for the detection of inovirus sequence. Overall, 6,570 non-redundant proteins were detected across 45 different pI-like protein clusters (PCs), with 16 singletons. These 45 different PCs gathered into 10 different iPFs ("inovirus Protein Families", Supplementary Fig. 3, see Methods). Two of these iPFs (iPF_00003 and iPF_00013) included a large number of mostly (> 95%) pI-like proteins (4,548 and 1,740 proteins, respectively). The few sequences in these iPFs that had not been previously identified as pI-like protein were usually genuine partial pI-like proteins too short to yield a significant hit, and thus unaffiliated. Both of these iPFs also included pI proteins from *Inoviridae* isolates, and were thus annotated as genuine pI proteins. The multiple alignments of the 34 PCs clustered into these 2 iPFs were visually inspected to verify that (i) the ATPase domain was most closely related to the inovirus Zot domain as opposed to one of the other known FtsK/HerA ATPase domains in the PFAM database (PF01580, PF01935, PF02534, PF03135, PF05872, PF06834, PF09378, PF09397, PF10412, PF11130, PF12538, PF12696, PF12846, and PF13491), and (ii) the sequence included a TMD to anchor the pI protein in the host membrane.

The 21 PCs clustered in iPF_00003 corresponded to sequences with a "typical" pI protein architecture, i.e. the ATPase domain is followed by a C-terminal extension with a TMD membrane (Supplementary Fig. 3). These PCs were mostly associated with gram negative hosts (with the exception of some prophages detected in Clostridia), and included all gram negative *Inoviridae* isolates (e.g. M13, CTX, etc). However, 3 PCs were identified that could not be

confirmed as likely inovirus pI protein: PC_00610, PC_01272, and PC_01338 were all most similar to the ATPase domain from archaeal turrivirus STIV, and were thus considered as false positives and removed from the inovirus dataset.

Conversely, the 13 PCs clustered in iPF_00013 displayed an "atypical" pI protein architecture: their Zot domain was not followed by a C-terminal extension, and the TMD was usually detected in the N-terminal part of the protein (or in the case of PC_00303 at the C-terminal tip of the sequence). These PCs are mostly composed of sequences found in gram positive hosts, and include *Inoviridae* isolated on *Propionibacterium*, *Thermus*, and *Spiroplasma* (Supplementary Fig. 3, Supplementary Table 3). The distribution of pI-like proteins in two distinct iPFs thus seems to be roughly correlated with the fundamental differences between cell membranes of gram negative hosts, associated with typical pI, and cell membranes of gram positive or wall-less hosts, associated with atypical pI. Among the PCs gathered into iPF_00013, one (PC_01836) was identified as likely false positive as it was most closely related to archaeal turrivirus STIV, and the associated sequences were excluded from the inovirus dataset.

Finally, for pI-like proteins outside of iPF_00003 and iPF_00013, genomes were individually inspected to evaluate whether these could also represent inovirus genomes. All but 1 of these sequences were identified as likely false positives based on the similarity of their pI-like sequence to another FtsK/HerA ATPase domain with higher scores than to the Zot domain. The only case of a putative genuine inovirus pI protein found outside of iPF_00003 and iPF_00013 was sequence 1066081_contig_758_11 found in iPF_00002. This iPF was annotated as an assembly protein, and the clustering of this specific sequence seemed to originate from a fusion of the pI (Morphogenesis) and pIV (Assembly) proteins (Supplementary Fig. 3). Interestingly, this potential fusion of pI and pIV genes has also been identified in PC_01246, annotated as a pI-like protein (part of iPF_00003). Notably, no other pI or pIV were identified in the genomes encoding these putative fusion proteins, and these sequences clearly included both conserved domains (Supplementary Fig. 3). In characterized inoviruses, the assembly domain (pIV) is encoded by a distinct gene and ensures the passage of the virion across the outer membrane in diderm hosts. Hence, these fused genes could produce a protein which would allow the passage of the virion across both host membranes, and accordingly these were all detected in diderm hosts. Although very atypical, these sequences including fused pI-pIV proteins were still included in the final dataset as they are likely functioning extrusion mechanisms, at least based on sequence analysis. Conversely, 5 other putative pI-pIV fusion proteins were detected in iPF_00002, but displayed a seemingly truncated Zot-like domain and lacked the typical TMD found in C-terminal of this Zot-like domain. These sequences were not considered as genuine pI-like proteins.

Eventually, this improved annotation of pI proteins was used to refine the final dataset of inovirus sequences. This included 4 sequences initially identified as "tandem prophages" which were reclassified as "regular genomes" since one of the two pI detections was a false positive, and 28 sequences removed from the dataset because their putative pI protein was found in one of the false positive PCs.

Evaluation of the automatic detection approach: challenges and limitations

To understand the challenges and possible limitations of our detection approach, we first checked which features were primarily used in the Random Forest classifier to identify an inovirus genome context (Supplementary Fig. 2). The three main features identified as most important based on their associated average decrease in Gini index were the number of putative inovirus coat proteins (i.e. predicted based on sequence length and detected TMD), the total number of predicted genes in the fragment, and the number of hits to *Inoviridae* PFs. On the other hand, the detection of the PFAM domain for the inovirus coat protein was the least important feature, most likely because the inovirus coat protein sequences are very divergent and typically don't display any recognizable level of sequence similarity even when using HMM-based methods.

Next, we examined the scores obtained from all genome fragments or contigs encoding a pI-like gene and which were evaluated using the Random Forest classifier (i.e. were not part of the initial set of manually curated inovirus sequences). While putative inovirus fragments (i.e. displaying a score ≥ 0.9) represented 50% of the candidate fragments derived from microbial genomes, they represented only 17% of the candidate fragments derived from metagenome assemblies (Supplementary Fig. 2). Notably, most of the discarded fragments were short (<5kb) contigs, which may not contain enough information to be reliably evaluated by the Random Forest classifier. Hence, a portion of the sequences which did not pass our selection criteria may be partial assemblies of inovirus genomes. Among the fragments selected based on a score ≥ 0.9 in the Random Forest classifier, only 2% (134 sequences) were subsequently identified as false positives upon manual inspection (see above). Hence, the Random Forest classifier used here seems to retain high specificity even when applied to sequences not included in the training set. Finally, we compared the distribution of scores between known and proposed inovirus families as well as false positives detections to evaluate any pattern linked to the processing of novel data (Supplementary Fig. 2). As could be expected, members of the Protoinoviridae, which include the canonical inoviruses such as Enterobacteria phages M13 and fd, are associated with higher scores than other proposed families (Supplementary Fig. 2). However, the median score for all groups was ≥ 0.975, much higher than the cutoff used of 0.9, including for proposed families such as the Photinoviridae which were strongly under-represented in the original training set. This suggests that, overall, the features used in the Random Forest classifier are broadly shared across the inovirus diversity. On the other hand, the median score for sequences identified as false-positives within the candidate inovirus sequences (with score > 0.9) was also > 0.975, and the score distribution of these false-positives was comparable to the one genuine inovirus sequences. This confirms that the features used here, while efficient to identify inoviruses, will tend to also identify other viruses and mobile genetic elements composed of short uncharacterized genes (see above).

Overall, while Random Forest classifiers have previously been successfully used to detect bacteriophage sequences from non-similarity-based genome features, e.g. in tools like PhiSpy[7] or MARVEL[8], these tools are trained on a broad set of publicly available phage genomes, and as such will tend to not be efficient for groups under-represented in genome databases such as

inoviruses. Here, the major difference in our approach is the selection of features such as putative inovirus coat proteins or hits to *Inoviridae* PFs, and the establishment of a large training set of 795 inovirus genomes, which enable the automatic detection of putative inoviruses. Nevertheless, this approach remains challenged by short input sequences and the presence of pI-like ATPases in other viruses or mobile genetic elements. Pragmatically, the former means that sub-optimal genome assemblies yielding short contigs (i.e. < 5kb) will lead to a large amount of false negatives, as these short contigs will not include enough information to identify them as putative inoviruses. Conversely, the presence of pI-like ATPases in other viruses or mobile genetic elements means that a manual inspection step of putative inovirus candidates (pI-containing fragments with a score ≥ 0.9) is required in order to identify these false-positives.

Types of inovirus sequences detected

Among the inovirus sequences detected, 1,709 were identified as putative complete inovirus genomes as these were either circular contigs (n=1,088), prophages with identified canonical attachment (att) site in a tRNA (n=311) or prophages with an identified canonical att site adjacent to an integrase-like gene (n=310, see Methods). An additional 1,586 fragments were putative complete prophages for which non-canonical att sites could be identified, i.e. the fragment is framed by direct repeats but these repeats are not within a tRNA gene or outside an integrase-like gene. Finally, the remaining fragments were either linear contigs likely from partial genomes (n=2,526) or prophages for which no att site could be identified (n=4,474). Notably, 553 fragments included multiple distinct pI-like proteins with no identifiable genome ends or attachment sites, and as such likely represent tandem prophage insertions, including possibly degraded prophages[9].

Distribution of inovirus sequences across metagenomes and biomes

The 5,917 inovirus sequences detected in metagenome assemblies, which included 3,677 species exclusively detected from metagenomes (Supplementary Fig. 4, Supplementary Table 3), can inform about the distribution of these viruses across ecosystems and geographic locations. Overall, individual species tend to be associated with a single sample type: 95% of species detected in multiple metagenomes are restricted to a single sample type (Supplementary Table 3). Inovirus sequences were detected in environments ranging from mesophilic (e.g. freswhater lakes) to 'extreme' (e.g. thermal springs or deep-ocean subsurface), from pristine (e.g. Antarctica) to strongly impacted by human activity (e.g. wastewater), from free-living microbial communities (e.g. ocean surface) to host-associated (e.g. human gut, rhizosphere), as well as on every continent and from the equator to the poles. Associated with their broad host range, the extensive ecological distribution of inovirus sequences suggests they have the potential to impact most of Earth's ecosystem, including modulating interactions between organisms within holobionts, as suggested by some available isolates[10].

Prevalence of inoviruses in microbial genomes

Based on the 2,289 inovirus species associated to a host, we calculated an estimated prevalence for inoviruses, i.e. the proportion of microbial genomes including an inovirus genome. The highest median prevalence was observed in Gamma- and Betaproteobacteria, where qualified genera (i.e. genera with ≥ 5 genomes) displayed on average > 10% of genomes with ≥ 1 detection(s) (Supplementary Fig. 5). Among these, prevalence in the genus *Xylella* was particularly high (87%), although these prophages were all associated with the microbial pathogen *Xylella fastidiosa*, and thus likely reflect the strong association of inoviruses with this specific species. Beyond these two groups, inoviruses were detected in ~1% of genomes on average, although this prevalence was > 15% for 5 genera (*Acidithiobacillus*, *Desulfosporosinus*, *Eubacterium*, *Lachnoclotridium,* and *Spiroplasma*), suggesting these might be evolving under an unusually high inovirus infection rate (Supplementary Fig. 5).

Curiously, 5 host genera composed of > 400 genomes did not yield any detection: *Mycobacterium* and *Streptomyces* (Actinobacteria, n=660 and 411, respectively), *Helicobacter* (Campylobacterota, n=443), *Lactobacillus* and *Staphylococcus* (Firmicutes, n=692 and 844 respectively). Since filamentous phages have been detected in other members of the same families, it is likely that members of these specific genera are very rarely (if ever) infected by inoviruses.

Co-infection patterns across host groups

Usually, a single inovirus sequence was detected per genome (76% of cases), and multiple detections were mostly found within the two host classes with high inovirus prevalence (Gamma- and Beta-proteobacteria, Supplementary Fig. 5). However, *Spiroplasma* represented an exception to this rule: beyond a unusually high level of inovirus prophages compared to other Tenericutes, these genomes also displayed an average of > 15 distinct detections, including 2 genomes with 24 and 25 distinct prophages detected (Supplementary Table 3). Although these data are only based on 5 *Spiroplasma* genomes in which prophages were detected, it suggests that at least some members of this genus may be uniquely able to integrate and maintain dozens of distinct inovirus genomes at a time, a feature previously hypothesized as driving the extensive intra-genome recombinations observed in this clade[11].

Inovirus prophages were frequently detected along with *Caudovirales* prophages: 1,573 bacterial genomes included signs of both types of viruses, consistent with a trend previously noted in smaller scale prophage analyses[12,13] (Supplementary Fig. 5). Curiously, these combined prophages insertions sometimes occurred at the same location in the host genome, particularly in Betaproteobacteria and Campylobacterota such as *Neisseria* and *Campylobacter* (Supplementary Fig. 5). Such co-localization could provide opportunity for horizontal gene transfer through imprecise excision, and more generally highlights a potential for direct virus-virus interactions, which have so far remained mostly unexplored[13].

Evaluation of the taxonomic rank represented by network-derived genome (sub)groups

ICTV guidelines are only available for genera (75% AAI) and species (95% ANI) in the *Inoviridae* family, such that we had to use other viral groups as reference to estimate which

taxonomic rank the groups and sub-groups defined based on gene content comparison represented. To this end, we compared the Amino Acid Identity percentage (AAI) of marker genes (i.e. pI-like proteins) from this extended set of inovirus genomes with other established viral groups at different ranks. For the order rank, we opted to use *Caudovirales* as references even though these are dsDNA viruses and tend to have larger genomes, since no classification at the order rank is available for small ssDNA viruses. For family and genus, we used established ssDNA taxonomy from the *Microviridae* and *Circoviridae* families, more comparable to inoviruses in terms of genome size and complexity.

This comparison to known viral taxonomic groups suggested that the 6 main groups observed on the inovirus sequence network are comparable to currently established viral families, while levels of similarity observed when comparing sequences between the 6 main groups were consistent with an order rank (Supplementary Fig. 7). Hence we propose that the *Inoviridae* family should be considered as a viral order instead, which we would propose to name *Inovirales* in accordance with standards in viral taxonomy nomenclature. This order would be tentatively divided into 6 candidate families, corresponding to the 6 mains groups established from the genome-PC network. Still based on AAI, the 212 sub-groups would be consistent with subfamilies, as these are more divergent than the established threshold used to define genera in the current *Inoviridae* family (75% AAI[14]) and more divergent than currently established ssDNA virus genera (Supplementary Fig. 7). As would be expected, all members of each current genus were found in a single proposed subfamily (Supplementary Table 3).

<u>Genome network topology and connector PCs</u>

Overall, only 20 PCs (out of 892 displayed on the network) connected genomes across proposed families (Fig. 5). All but 3 of these (i.e. 85%) were functionally affiliated, including 4 pI-like, 3 structural, and 7 replication-associated proteins, suggesting these "connector" PCs (*sensu* [15]) likely represent some of the most conserved genes across inovirus genomes. None of these PCs however connected substantially (>50% proposed subfamilies) to more than 1 proposed family, suggesting that these most likely reflect events of horizontal gene transfer or convergent evolution involving these conserved genes. This further illustrates the complex evolutionary history of these genomes for which no single gene seems to be both conserved and exclusively (or near-exclusively) vertically inherited (Supplementary Fig. 7).

<u>Characteristics and proposed names for proposed families</u>

The two largest proposed families (in dark blue and teal in Fig. 5) comprise 3,576 and 1,020 genomes, respectively, and include all isolates officially classified into the seven *Inoviridae* genera currently recognized by the ICTV (Supplementary Table 3). The first of these two proposed families (in teal in Fig. 5) includes members of genera *Fibrovirus, Habenivirus, Inovirus, Lineavirus* and *Saetivirus*, and so gather the prototypical and most characterized isolated *Inoviridae.* The second one (dark blue) comprises members of the genus *Vespertilinovirus* and the single member of the *Plectrovirus* genus. Hereinafter, we refer to these two putative families as "Protoinoviridae" (for the inovirus prototypical members) and

"Vespertilinoviridae" (inspired by the main genus of this proposed family), respectively. The proposed "Protoinoviridae" family comprises genomes nearly exclusively associated with Gamma- and Beta-proteobacteria, while the "Vespertilinoviridae" include mostly genomes associated with Clostridia and Tenericutes (Fig. 5, Supplementary Fig. 7). A third putative family includes the remaining isolates unassigned to a genus yet. These genomes tend to be smaller than those of other inoviruses (median size of 6.1kb), thus, we propose to name this candidate family "Paulinoviridae" (from 'paulus', latin for little/small). "Paulinoviridae" genomes are primarily detected in hosts affiliated to Actinobacteria, CPR, and Deinococcus-Thermus clades (Supplementary Fig. 7).

The remaining three proposed families do not include any viral isolate, but two of these exhibit specific genome features (Supplementary Fig. 7). The first putative family is composed of large genomes (median 9.4kb); hence we proposed to name this group "Amplinoviridae" (from "amplus", latin for large). The second one is composed of genomes with high coding density (i.e. more genes for comparable genome size, median number of genes=16) and we propose to name this assemblage "Densinoviridae" (Supplementary Fig. 7). Members of the "Amplinoviridae" are largely associated with hosts from Deltaproteobacteria and Campylobacterota, whereas "Densinoviridae" are predominantly found in Bacilli and Chloroflexi (Supplementary Fig. 7). Notably, two sequences in the "Densinoviridae" have been previously described as "cryptic plasmids" in Bacilli[16]. Similarities between small plasmids and filamentous phages have long been noted, and the boundary between the two types of mobile genetic elements seems tenuous at best[17]. However, filamentous particles have been induced from similar bacteria[18], and we have identified putative capsid proteins, a hallmark of viruses, encoded by members of the "Densinoviridae" (see main results and Supplementary Fig. 8). Hence, given that inoviruses have been frequently confused with plasmids (e.g. NC_002473 and NC_010429), these sequences are likely to correspond to genuine inovirus genomes. The last proposed family includes the only inoviruses associated with photosynthetic Cyanobacteria, hence we propose to name this candidate family "Photinoviridae".

Although the proposed families were defined exclusively from gene content analysis, they exhibited specific genome features and host ranges which suggested they indeed represent coherent groups. First, proposed families differ in terms of genome size and number of genes predicted. Notably, the median genome size within candidate families varied from 6kb ("Paulinoviridae") to 9.5kb ("Amplinoviridae"), and although most groups encoded a median of 11 to 13 genes per genome, one ("Densinoviridae") displayed a median number of genes of 16 (Supplementary Fig. 7). In addition, each of the proposed family is associated with a specific host range, with very little overlap (Supplementary Fig. 7): of the 70 host families with at least 2 inovirus sequences detected, 61 were associated with a single proposed inovirus family, 6 are associated with 2 proposed inovirus families, and only 3 are associated with 3 proposed inovirus families (*Peptococcaceae*, *Paenibacillaceae*, and *Bacillaceae*, all in the Firmicutes phylum, Supplementary Table 3).

Contrasting with their host range, the proposed inovirus families were not structured by biome or ecosystem type (Supplementary Fig. 7). All six candidate families seem to be detected in

virtually every type of environment, and the cases of non-detection are associated with under-sampled groups (i.e. proposed families with < 400 species). These data can however point toward which specific biome to sample in priority when targeting individual candidate families: “Vespertilinoviridae” and “Amplinoviridae” seem to be enriched in human-associated samples, “Densinoviridae” in “extreme” aquatic environments such as deep sub-surface, thermal spring, and hypersaline lakes, while both “Paulinoviridae” and “Photinoviridae” are preferentially detected in soil samples.

Identification and annotation of putative archaea-associated inoviruses

The four putative proviruses were identified in the genome sequences of three isolates, two affiliated to *Methanolobus* and one to *Methanosarcina*, all in the *Methanosarcinacea* family of the phylum Euryarchaeota, as well as one metagenome-assembled genome (MAG) affiliated to the Aenigmarchaeota candidate phylum. The contigs composing this MAG were inspected to confirm that they represented a single and cohesive population genome, and no sign of contamination, i.e. presence of a contig affiliated to a different microbial genome, could be identified. The gene content of these different inoviruses was consistent with their respective host: the 3 *Methanosarniacea*-associated viruses displayed little to no sequence similarity to the sequence detected in the Aenigmarchaeota MAG (Fig. 4A).

The two sequences detected in *Methanolobus* included the full repertoire of genes expected in a genuine inovirus, including a morphogenesis (pI) protein with an N-terminal TMD typical of inoviruses infecting monoderm hosts, an integrase gene, genes predicted to encode putative structural proteins based on sequence length and presence of a single TMD, as well as a gene encoding a rolling-circle replication initiation protein[19]. This gene complement strongly suggests that these two sequences represent fully functional inoviruses, since they include the full suite of genes required for genome integration, replication, encapsidation, and extrusion (Fig. 4A). The detection of genes predicted as structural, i.e. short genes with a single TMD, is especially noticeable given that only 0.69% of all genes in Euryarchaeota display features characteristic of structural proteins of inoviruses (30-90 aa, 1 TMD). The predicted proviruses are thus more likely to be inoviruses than any other type of mobile genetic element.

The putative proviruses identified in *Methanosarcina* displayed genes for integration, morphogenesis, and putative structural proteins, but no recognizable gene involved in genome replication. Similarly, the sequence identified in the Aenigmarchaeota MAG only included a morphogenesis gene and a putative structural protein. Hence, these two latter sequences could be partial genomes, possibly remnants from a decaying provirus, or could be complete genomes of active viruses for which replication-associated gene(s) cannot yet be identified, as is common amoung archaeal viruses[20]. Regardless of the completeness of these genomes, both include an inovirus-like morphogenesis protein suggesting these are most likely inoviruses. In addition, we found a perfect match between the Aenigmarchaeota provirus and a CRISPR spacer from a different contig in the same MAG, which is also consistent with it being a provirus (Supplementary Table 6).

PCR validation of excision for an inovirus integrated in *Methanolobus profundi* MobM

Attempts at observing inovirus capsids through TEM were unsuccessful because *Methanolobus* MobM flagella are similar in structure, length, and width to filamentous virions[21]. Thus, we used instead PCR to detect the presence of a circularized form of the provirus, which would correspond to the complete genome being excised and replicated or encapsidated (Fig. 4B). We first verified that the genome sequencing and assembly was correct by amplifying a product internal to the predicted provirus and a product spanning the predicted insertion site (Fig. 4B). In both cases, we obtained a successful amplification with products of the expected size, confirming that the predicted provirus is present and likely integrated in most cells in the culture. Notably, we obtained positive amplification for the product spanning the insertion site from the fraction < 0.22 µm, which suggests that some MobM cells can pass through the 0.22 µm filter typically used to separate viruses from their bacterial or archaeal hosts.

Next, we designed a PCR primer pair specific to the predicted excised form of the virus genome by combining a forward primer from the 3' end of the provirus to a reverse primer in the 5' end of the provirus (Fig. 4B). We obtained a product of the expected size, and sequencing of the product confirmed that it spanned both ends of the predicted provirus in the predicted orientation and at the expected coordinates. This latter PCR reaction initially generated more nonspecific products than the internal or integration site primers, and the reaction annealing temperature had to be increased (> 56°C) to obtain a single band at the expected size. This higher level of nonspecific amplification combined with the fact that the product obtained yielded a relatively faint band (Fig. 4B) suggests that the template for this reaction, i.e. the excised form of the virus genome, is found in a much smaller fraction of cells than the integrated form. It is thus very likely that under laboratory conditions, even after treatment with mitomycin C, the provirus is repressed in most cells resulting in an overall low concentration of circular virus genomes.

Additional host associations from CRISPR spacer matches to metagenome-assembled inoviruses

Matches between CRISPR spacers and inovirus sequences included both predicted prophages/ proviruses for which host information could be confirmed (n=711) and metagenome assemblies for which additional host information could be obtained (n=439, Supplementary Table 6). Near-exact matches (i.e. 0 or 1 mismatch) between CRISPR spacers and metagenome-derived viral contigs have been shown to reliably associate uncultivated viral genomes to putative host(s)[22]. Here, the reliability of near-exact CRISPR matches (i.e. allowing at most 1 mismatch over the entire spacer length) was confirmed by the CRISPR-based host links assessed for prophage predictions: in 99.5% of the cases, host affiliations were consistent (708 of 711). The three outliers might be resulting from false positive spacer matches, horizontal virus transfer, or a very broad host range for certain inoviruses. It is of note that introduction of the genome of an inovirus infecting *Clostridium*, a gram-positive bacterium, into the gram-negative *Escherichia coli* resulted in production of filamentous virus-like particles[23], suggesting that host switches might not be strictly prohibited among inoviruses. Nevertheless, the overall agreement between spacer matches and host affiliation of prophages suggest that spacer matches to metagenome-

derived inovirus sequences can be used confidently, expanding the number of host-associated sequences to 439 additional putative inovirus species.

Most of the host pairings derived from these metagenome CRISPR spacer matches were found in hosts groups for which prophages had already been detected, and only 4 additional orders were identified. First, 2 inovirus sequences were associated to *Roseiflexus* genomes in the *Chloroflexales* order. Other sequences from the same phylum (*Chloroflexi*) had been linked to inovirus sequences, and all these *Chloroflexi*-associated sequences were consistently affiliated to the proposed "Densinoviridae" candidate family. In addition, these metagenome-derived inovirus sequences were detected in a hot spring metagenome, consistent with the preferential habitat of *Roseiflexus.*

Another 2 species were associated with an *Aphanizomenon* genome (genus of photosynthetic Cyanobacteria). These 2 putative viral sequences were consistently affiliated to the "Photinoviridae" proposed family, which gathers all inoviruses associated with photosynthetic Cyanobacteria, and consistently originated from two freshwater lake metagenomes (sampled from Lake Mendota).

One inovirus species was associated with a genome assembled from a *Nasutitermes corniger* (a species of termite) metagenome and currently affiliated as an "Unclassified Fibrobacteria". No prophage had been detected associated with this specific host phylum so far. Consistently, the inovirus species was also assembled from a termite gut metagenome.

Another inovirus species was associated with a genome affiliated to the Nitrospinae phylum-level group. This inovirus species is classified in the "Amplinoviridae" Subfamily 4, which includes Deltaproteobacteria-associated inoviruses. This is consistent with Nitrospinae and Deltaproteobacteria being related groups of bacteria. The inovirus genome was assembled from a groundwater metagenome as was the bacterial genome.

Finally, one species was associated for the first time to *Caldicellulosiruptor obsidiansis*, a host in the *Thermoanaerobacterales* order, part of the *Clostridia* class for which other putative inovirus sequences had been detected. This sequence was consistently affiliated to the proposed subfamily Sf_1 of the "Vespertilinoviridae" candidate family, the main group of Clostridia-infecting inovirus sequences identified in this study, and detected in a hot spring metagenome, which is consistent with the known preferential habitat of *Caldicellulosiruptor obsidiansis*.

<u>Evaluation of hypothetical proteins from self-targeted inoviruses in a *Pseudomonas aeruginosa* model</u>

Hypothetical proteins from two self-targeted *Pseudomonas* inovirus prophages for which no Acr locus could be identified elsewhere in the genomes were synthesized and cloned in a pHERD30T vector for expression in *Pseudomonas aeruginosa* (Supplementary Fig. 10). Two of these candidate genes (2687473922 and 2687473921) were toxic when expressed in the host, and their putative Acr or superinfection exclusion activity could not be assessed. However, these genes may be components of uncharacterized toxin-antitoxin systems.

Two candidate genes demonstrated superinfection exclusion activity, which was manifested by the absence of plaques at dilutions for which plaques were formed for the same phage in the

same host transformed with the empty vector (Supplementary Fig. 10). Neither of the 2 genes provided universal superinfection exclusion: gene 2687473927 prevented or limited infection of host strain PAO1 by 3 of the 6 phages tested, but no effect could be observed in the PA14 strain. By contrast, 2687473923 did not provide any superinfection exclusion in host PAO1, but prevented infection of 1 of the 3 phages efficiently infecting PA14 (Supplementary Fig. 10). This suggests that inovirus-derived superinfection exclusion activity varies depending on the host strain and the co-infecting virus. Specifically, gene 2687473927 seems to have a relatively broad spectrum and could provide a general fitness advantage to host PAO1 by limiting infection in this specific host strain for both temperate Mu-like siphoviruses (DMS3m and JBD30) and lytic T7-like podoviruses (KMV). Conversely, the effect of 2687473923 seems to be much more restricted, and points toward more specific virus-virus interactions or incompatibility between the inovirus and phage JBD30.

Although both proteins are uncharacterized, they are relatively widely distributed in inoviruses, forming two corresponding protein families: iPF_00048 for gene 2687473923 and iPF_00082 for gene 2687473927. Members of the iPF_00048 protein family, responsible for the "narrow" superinfection exclusion, were found in 424 distinct inovirus species. These inoviruses were affiliated across 9 proposed subfamilies within the "Protoinoviridae", and associated with both Beta- and Gammaproteobacteria hosts. Since some members of this protein family contain an HTH domain, we posit that these genes may be coding for transcriptional regulators that could provoke incompatibility with some individual phages, but their primary function might not be superinfection exclusion.

Members of the iPF_00082 protein family ("broad" superinfection exclusion) were detected in 163 distinct inovirus species, all affiliated to the "Protoinoviridae" and nearly all (98%) to the "Protoinoviridae:Sf_2" proposed subfamily. All identified hosts for these species were affiliated to the *Pseudomonas* genus. This narrow distribution in terms of inovirus family/subfamily and host range suggests that members of this protein family have evolved in *Pseudomonas*-specific inoviruses to mediate broad-spectrum superinfection exclusion. Strikingly, nearly half of the inovirus prophages identified in *Pseudomonas* genomes (44%, 158 of 359) encoded this gene. This could be due to positive selection of this gene in inovirus prophages because of its superinfection exclusion properties, although we cannot exclude a potential bias in the *Pseudomonas* genome dataset whereby many strains of *Pseudomonas aeruginosa* with distinct but closely related inovirus prophages would have been sequenced. Finally, all members of the iPF_00082 protein family are 29-30 aa-long and carry predicted α-helical membrane-spanning domain, suggesting that superinfection exclusion may occur at the host cell surface, possibly during the attachment and/or entry of a superinfecting phage. Notably, several *Pseudomonas* dsDNA prophages have already been shown to provide superinfection exclusion through alteration of the host T4 pilus[24], which could be the case as well for these inovirus-encoded proteins.

Supplementary Figures

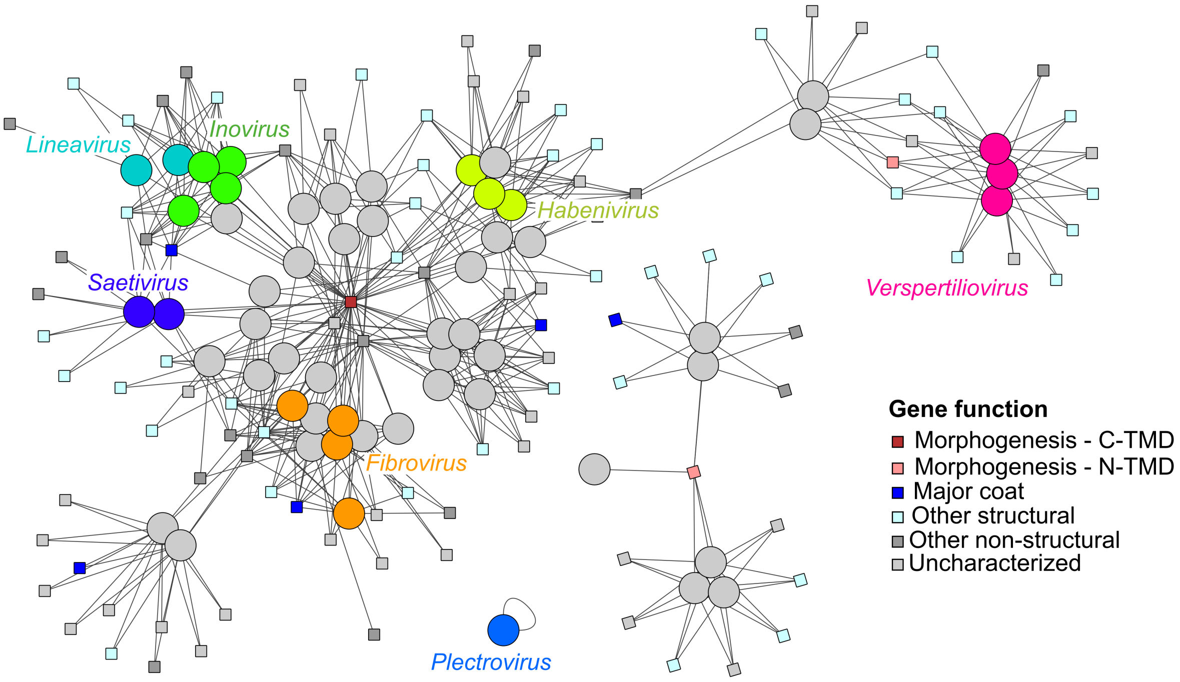

Supplementary Figure 1. Genome-gene bipartite network of publicly available *Inoviridae* isolate genomes. Genomes are represented as circles colored according to their genus classification, and protein families (PFs) are displayed as squares colored by their predicted function. ICTV-proposed genera are indicated by coloring of the genome nodes. Morphogenesis (pI-like) proteins are highlighted in shades of red, and although these proteins were represented by 3 distinct protein families, local similarities could still be detected between the corresponding HMM profiles (HHSearch probability ≥ 90%). The two types of pI-like proteins, with the transmembrane domain (TMD) either in C- or N-terminal are indicated in dark and light red respectively (see Supplementary Fig. 3).

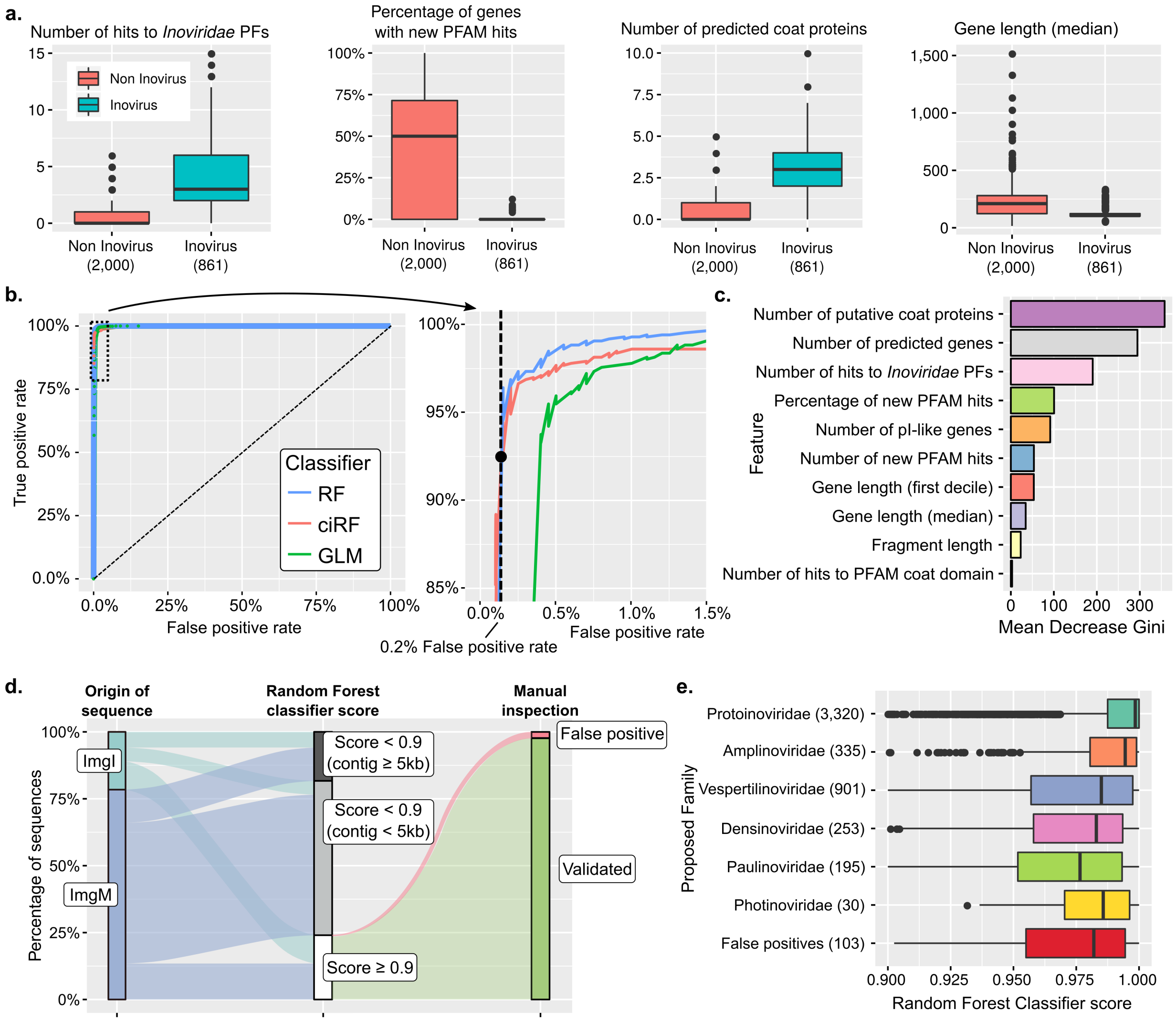

Supplementary Figure 2. Features used and characteristics of the Random Forest classifier used to detect inoviruses. A. Example of genome features evaluated on isolate inoviruses and manually-curated inovirus prophages (in blue) and other fragments from microbial genomes used in the negative training set (in red). Boxplot lower and upper hinges correspond to the first and third quartiles, whiskers extend no further than ±1.5*Inter-quartile range. The number of sequences in each set is indicated below each boxplot. B. ROC curve of the automatic classifier distinguishing inovirus genomes from other viral or microbial genome fragments. A subplot displays a zoom on the area < 2% false positive rate and > 85% true positive rate. The three types of classifier tested are plotted in different colors, and the true positive and false positive rates associated with the chosen threshold of 0.9 for the random forest classifier are indicated with a black circle and dotted line on the subplot. RF: Random Forest, ciRF: conditional inference Random Forest, GLM: Generalized Linear Model. C. Importance of the different features in the Random Forest classifier, measured through the average decrease of Gini index. D. Origin and

validation of inovirus sequences identified through the Random Forest classifier (i.e. not in the initial set of 805 manually curated genomes used for training). The left bar indicates whether the sequence comes from a microbial genome (ImgI) or a metagenome assembly (ImgM). The middle bar shows the result of the Random Forest classifier using a cutoff of 0.9 on the confidence score, and separating for the sequences with a score < 0.9 between short (<5kb) and long (≥ 5kb) contigs. Finally, the right bar indicates, for sequences with a score ≥ 0.9, whether the sequence was identified as a false positive during the manual inspection step (see Supplementary Text). E. Score obtained for sequences identified from the Random Forest classifier (i.e. not in the initial set of 805 manually curated genomes) grouped by proposed family or identified as non-inovirus sequences in the manual inspection step. Boxplot lower and upper hinges correspond to the first and third quartiles, whiskers extend no further than ±1.5*Inter-quartile range. The number of sequences in each set is indicated below each boxplot.

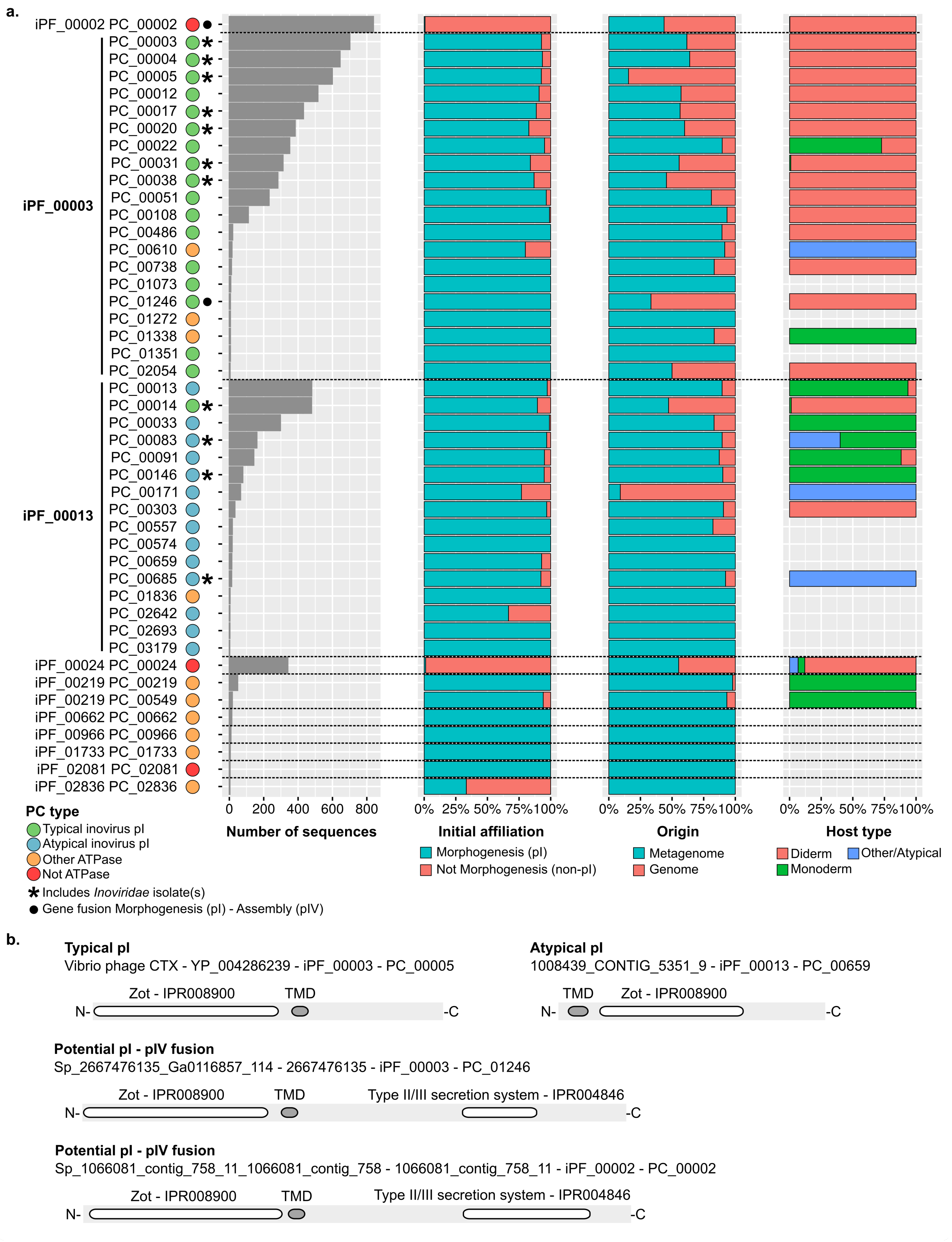

Supplementary Figure 3. Identification of genuine inovirus pI proteins. A. Characteristics of

protein clusters (PCs) including pI-like ("Morphogenesis") proteins, i.e. proteins with a best hit to a pI-like model, and used as seed to identify inovirus sequences. Each PC is associated with a protein family (iPF), the number of proteins in the cluster, their initial affiliation, their origin (genome or metagenome), and host information for the ones identified in microbial genomes. B. Schematic representations of the different types of pI proteins identified: typical with an N-terminal Zot-like domain followed by a transmembrane domain (TMD), atypical with an N-terminal TMD followed by a Zot-like domain, and potential pI – Assembly fusions including an N-terminal Zot-like domain followed by a TMD and a secretion system-like domain.

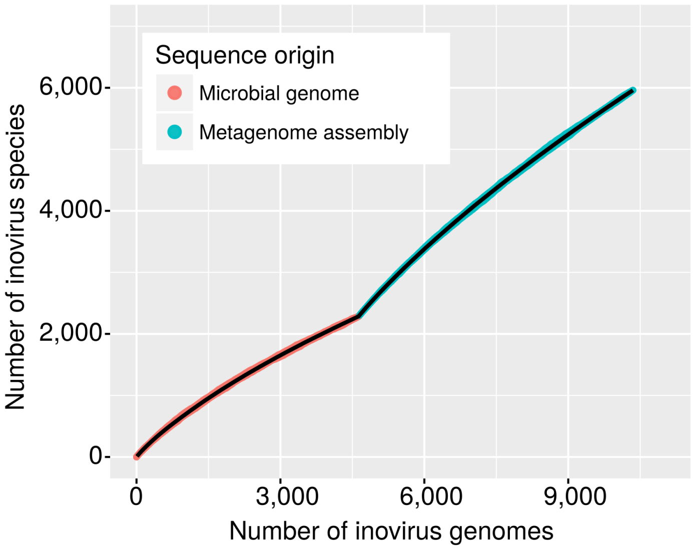

Supplementary Figure 4. Accumulation curves of inovirus species. The number of different species is indicated as a function of the total number of complete and partial genomes, first for
595 detections in draft and complete genomes from bacteria and archaea, and then in metagenome assemblies. A set of 10 subsample replicates were calculated and are plotted in colors, while the resulting average number of species is plotted in black.

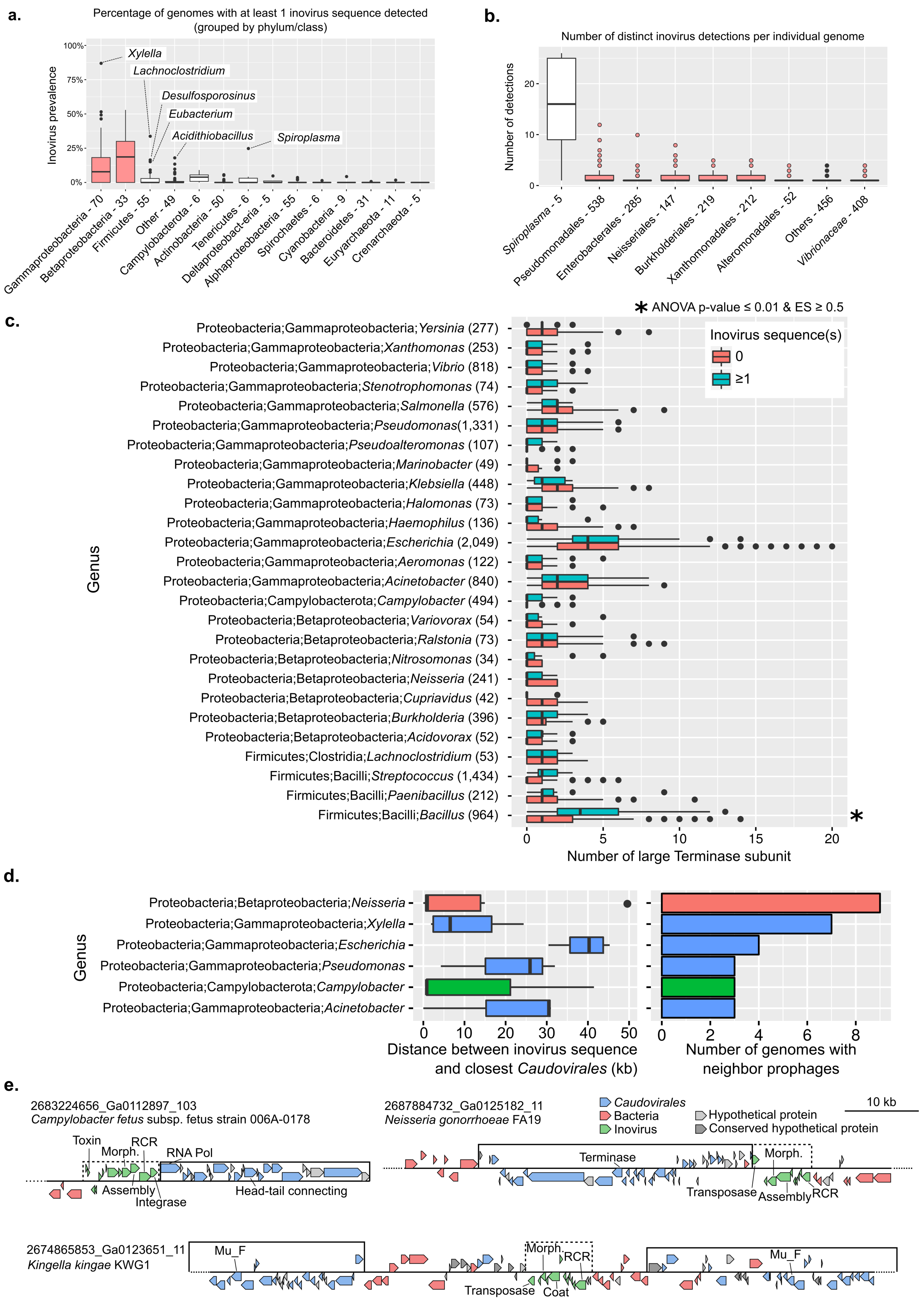
a.
Percentage of genomes with at least 1 inovirus sequence detected (grouped by phylum/class)
Inovirus prevalence
100%
75%
50%
25%
0%
Xylella
Lachnoclostridium
Desulfosporosinus
Eubacterium
Acidithiobacillus
Spiroplasma
Gammaproteobacteria - 70
Betaproteobacteria - 33
Firmicutes - 55
Other - 49
Campylobacterota - 6
Actinobacteria - 50
Tenericutes - 6
Deltaproteobacteria - 5
Alphaproteobacteria - 55
Spirochaetes - 6
Cyanobacteria - 9
Bacteroidetes - 31
Euryarchaeota - 11
Crenarchaeota - 5
b.
Number of distinct inovirus detections per individual genome
Number of detections
20
10
0
Spiroplasma - 5
Pseudomonadales - 538
Enterobacterales - 285
Neisseriales - 147
Burkholderiales - 219
Xanthomonadales - 212
Alteromonadales - 52
Others - 456
Vibrionaceae - 408
c.
* ANOVA p-value ≤ 0.01 & ES ≥ 0.5
Inovirus sequence(s)
0
≥1
Genus
Proteobacteria;Gammaproteobacteria;Yersinia (277)
Proteobacteria;Gammaproteobacteria;Xanthomonas (253)
Proteobacteria;Gammaproteobacteria;Vibrio (818)
Proteobacteria;Gammaproteobacteria;Stenotrophomonas (74)
Proteobacteria;Gammaproteobacteria;Salmonella (576)
Proteobacteria;Gammaproteobacteria;Pseudomonas(1,331)
Proteobacteria;Gammaproteobacteria;Pseudoalteromonas (107)
Proteobacteria;Gammaproteobacteria;Marinobacter (49)
Proteobacteria;Gammaproteobacteria;Klebsiella (448)
Proteobacteria;Gammaproteobacteria;Halomonas (73)
Proteobacteria;Gammaproteobacteria;Haemophilus (136)
Proteobacteria;Gammaproteobacteria;Escherichia (2,049)
Proteobacteria;Gammaproteobacteria;Aeromonas (122)
Proteobacteria;Gammaproteobacteria;Acinetobacter (840)
Proteobacteria;Campylobacterota;Campylobacter (494)
Proteobacteria;Betaproteobacteria;Variovorax (54)
Proteobacteria;Betaproteobacteria;Ralstonia (73)
Proteobacteria;Betaproteobacteria;Nitrosomonas (34)
Proteobacteria;Betaproteobacteria;Neisseria (241)
Proteobacteria;Betaproteobacteria;Cupriavidus (42)
Proteobacteria;Betaproteobacteria;Burkholderia (396)
Proteobacteria;Betaproteobacteria;Acidovorax (52)
Firmicutes;Clostridia;Lachnoclostridium (53)
Firmicutes;Bacilli;Streptococcus (1,434)
Firmicutes;Bacilli;Paenibacillus (212)
Firmicutes;Bacilli;Bacillus (964)
*
0
5
10
15
20
Number of large Terminase subunit
d.
Genus
Proteobacteria;Betaproteobacteria;Neisseria
Proteobacteria;Gammaproteobacteria;Xylella
Proteobacteria;Gammaproteobacteria;Escherichia
Proteobacteria;Gammaproteobacteria;Pseudomonas
Proteobacteria;Campylobacterota;Campylobacter
Proteobacteria;Gammaproteobacteria;Acinetobacter
0
10
20
30
40
50
Distance between inovirus sequence and closest Caudovirales (kb)
0
2
4
6
8
Number of genomes with neighbor prophages
e.
2683224656_Ga0112897_103
Campylobacter fetus subsp. fetus strain 006A-0178
Toxin
RCR
Morph.
RNA Pol
Assembly
Integrase
Head-tail connecting
2687884732_Ga0125182_11
Neisseria gonorrhoeae FA19
Caudovirales
Bacteria
Inovirus
Hypothetical protein
Conserved hypothetical protein
10 kb
Terminase
Morph.
Transposase
Assembly
RCR
2674865853_Ga0123651_11
Kingella kingae KWG1
Mu_F
Morph.
RCR
Mu_F
Transposase
Coat

Supplementary Figure 5. Inovirus prevalence and co-infection patterns. A. Prevalence of inoviruses estimated through the proportion of genomes within a genus with 1 or more inovirus detection(s). Beta- and Gammaproteobacteria are highlighted in red. Groups with unusually high inovirus prevalence, > 75% within beta- or gamma-proteobacteria or > 15% otherwise, are labeled on the plot. B. Distribution of the number of distinct detection(s) by genome, grouped by host phylum or class. Host groups are colored as in panel A. C. Distribution of the number of large terminase subunits (TerL) as a proxy for the number of *Caudovirales* prophages identified by genome for each genus where ≥ 10 genomes had an inovirus detection and ≥ 10 genomes had no inovirus detection. The genus for which the distribution of prophage number was statistically different between the two categories (*Bacillus*) is highlighted with a star (ANOVA p-value = 1.65e-07 & Cohen's effect size = 0.76, degree of freedom=1). D. Distribution of the distance between an inovirus prophage and the closest *Caudovirales* prophage for cases where the two sequences are less than 50kb apart. Distribution was plotted for genera where ≥ 3 cases of neighboring prophages were identified. Boxplot lower and upper hinges correspond to the first and third quartiles, whiskers extend no further than ±1.5*Inter-quartile range. Boxes are colored by host class. For panels A, B, C, and D, prevalence and co-infection frequencies were calculated after clustering near-clonal host genomes based on pairwise ANI (cutoffs: 95% nucleotide identity on 95% alignment fraction). For all boxplots, lower and upper hinges correspond to the first and third quartiles, whiskers extend no further than ±1.5*Inter-quartile range. E. Examples of (near-)contiguous inovirus and *Caudovirales* prophages. Three genome regions encoding both the inovirus and the *Caudovirales* prophages are displayed, with genes colored according to their affiliation. Prophages are highlighted with a solid black line (*Caudovirales*) or dashed black line (inovirus). For all boxplots, the number of observations for each group is indicated next to the group name, except for D where the number of observations is displayed as a bar chart (right panel).

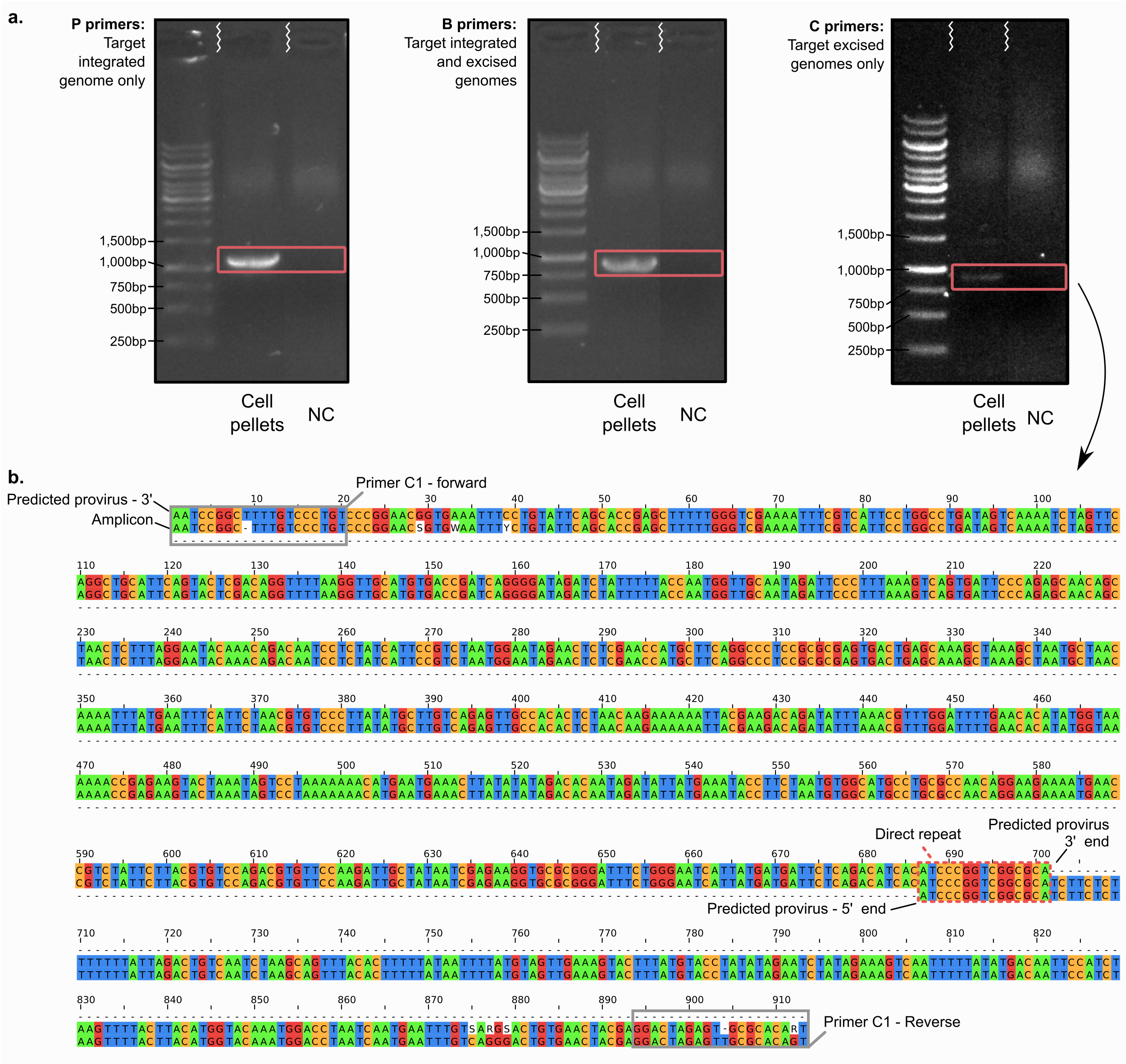

Supplementary Figure 6. Experimental validation of predicted provirus in *Methanolobus profundi* MobM. A. Amplification result for the three primer pairs tested. P primers amplify across the predicted 5' insertion site (left), B primers amplify within the predicted provirus (center), and C primers amplify across the junction of the predicted excised circular genome (right). P and B primers amplifications were repeated twice, and the C primers amplifications were repeated three times, with an identical result obtained for each replicated (Supplementary Fig. 11). NC: no template control. B. Amplification products obtained with the C primer (i.e. spanning the junction of the predicted excised genome) aligned against the genome sequence of *Methanolobus profundi* MobM. Top track represents the 3' region of the provirus, bottom track the 5' region of the provirus, and the middle track is the sequenced amplicon. The direct repeat predicted as the end of the provirus is framed in red. Since the amplicon aligned across this direct

repeat and from the 3' to the 5' end of the provirus, it is most likely derived from a circular excised version of the virus genome.

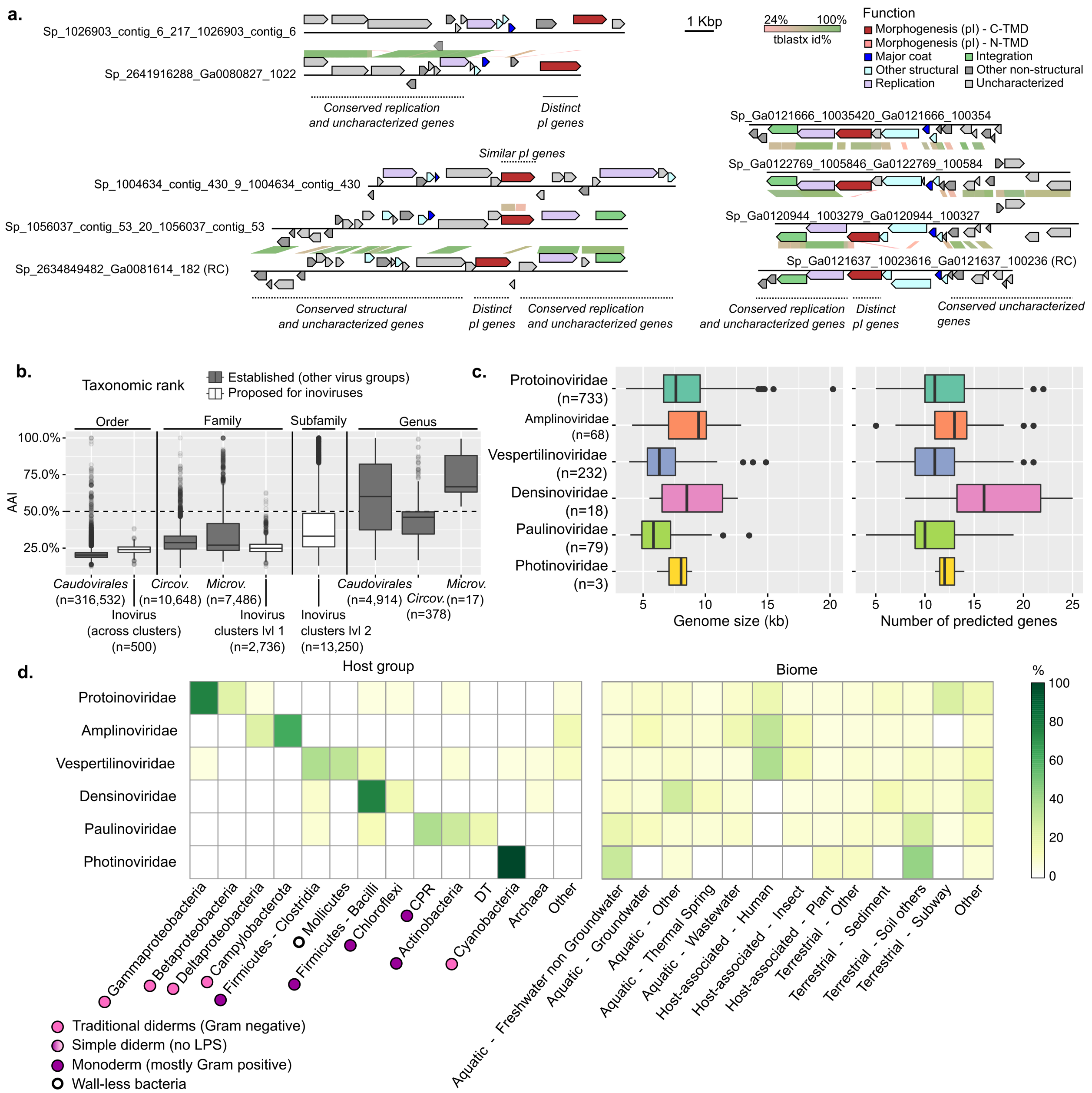

Supplementary Figure 7. Characteristics of the genome-based inovirus classification. A. Examples of inovirus genomes with partial gene content sharing. Three comparisons of predicted inovirus genomes highlighting the fact that some of these viruses can display nearly-identical genes but show no similarity between morphogenesis (pI-like) proteins. Genes are colored according to their functional affiliation, based on the iPF clustering (Supplementary Table 5). B. Distribution of pairwise marker gene Amino Acid Identity (AAI) for different viral groups and taxonomic ranks. Marker genes used included pI (Morphogenesis) for inoviruses, TerL (large terminase subunuit) for *Caudovirales*, Rep (replication initiation protein) for *Circoviridae*, and VP1 (major capsid protein) for *Microviridae*. Boxplots are colored according to the taxonomic ranks of the sequences compared. A dashed horizontal line indicates the threshold recently

proposed to delineate *Inoviridae* genera (50% AAI). *Circov.: Circoviridae*, *Microv.*: *Microviridae*. Boxplot lower and upper hinges correspond to the first and third quartiles, whiskers extend no further than ±1.5* Inter-quartile range. C. Characteristic genome features of proposed families. Boxplots show the distribution of genome size (left) and number of predicted genes (right) for each proposed family, colored as in Fig. 5. Genome size and number of predicted genes were only calculated on inovirus genomes reliably predicted as complete, i.e. isolates, circular contigs, or proviruses with a confident insertion site either in a tRNA or next to an integrase gene. Boxplot lower and upper hinges correspond to the first and third quartiles, whiskers extend no further than ±1.5*Inter-quartile range. D. Host and biome range of proposed inovirus families. For each candidate family, the percentage of species associated with a specific host group (left) or ecosystem type (right) is indicated. Only host groups and biomes associated with > 10% of the species of at least 1 candidate family are indicated separately, the remaining are gathered in the "Other" category. Type of membrane for host cells are derived from ref.[25]. DT: Deinococcus-Thermus. For boxplots (panels b and c), the number of observations is indicated between brackes.

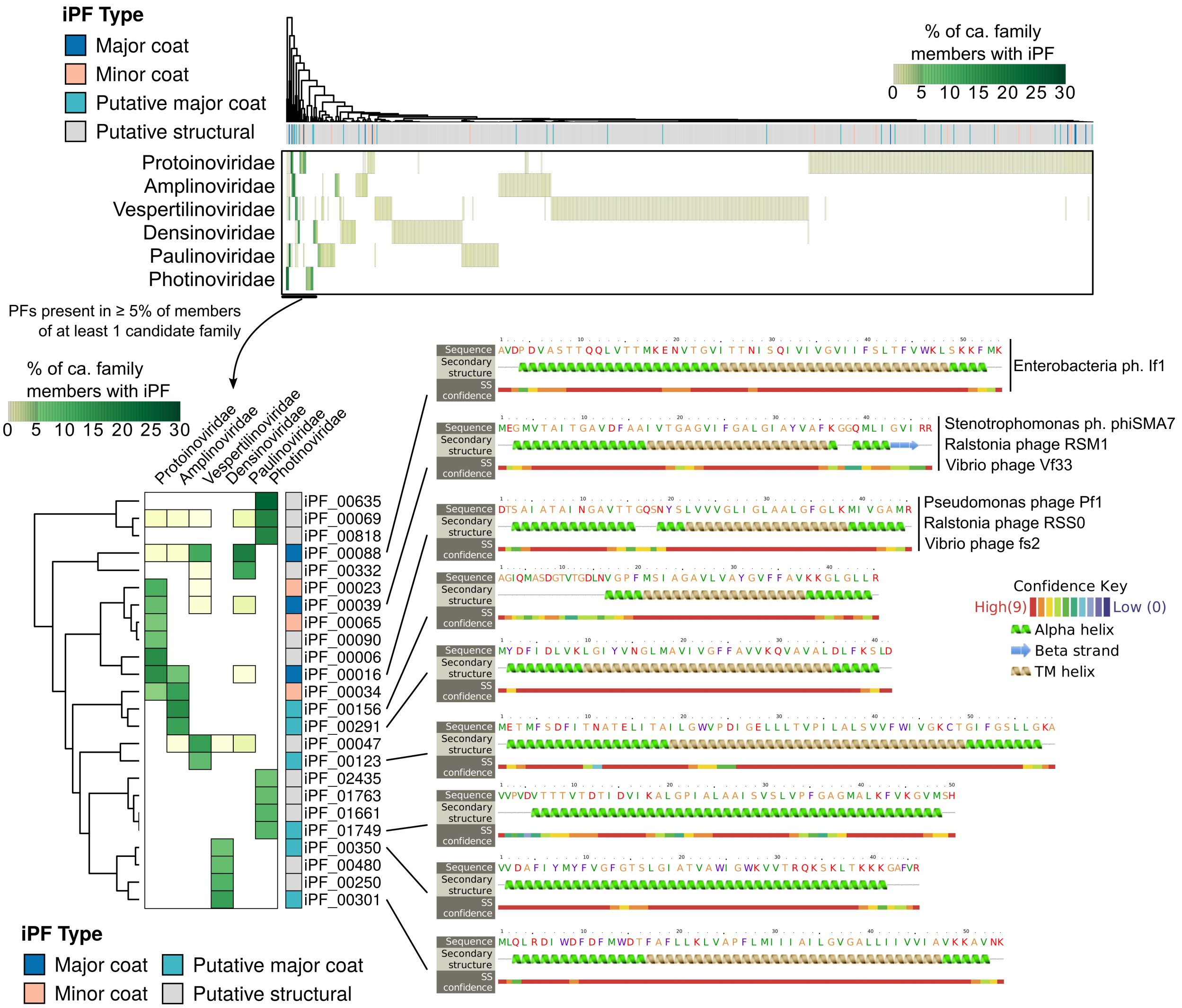

Supplementary Figure 8. Distribution of structural proteins and toxin/antitoxin proteins across inovirus species. Diversity of structural proteins across inovirus sequences. The top heatmap displays the relative abundance of each (putative) structural protein family (iPF) in each proposed family. Color scale represents the percentage of members of the proposed family encoding each iPF. A zoomed heatmap displaying only iPFs found in ≥ 5% of members of ≥ 1 proposed family is displayed in the bottom left corner. Secondary structure predictions obtained from Phyre2[26] are displayed on the right side for the most abundant iPFs predicted as major coat proteins for each candidate family.

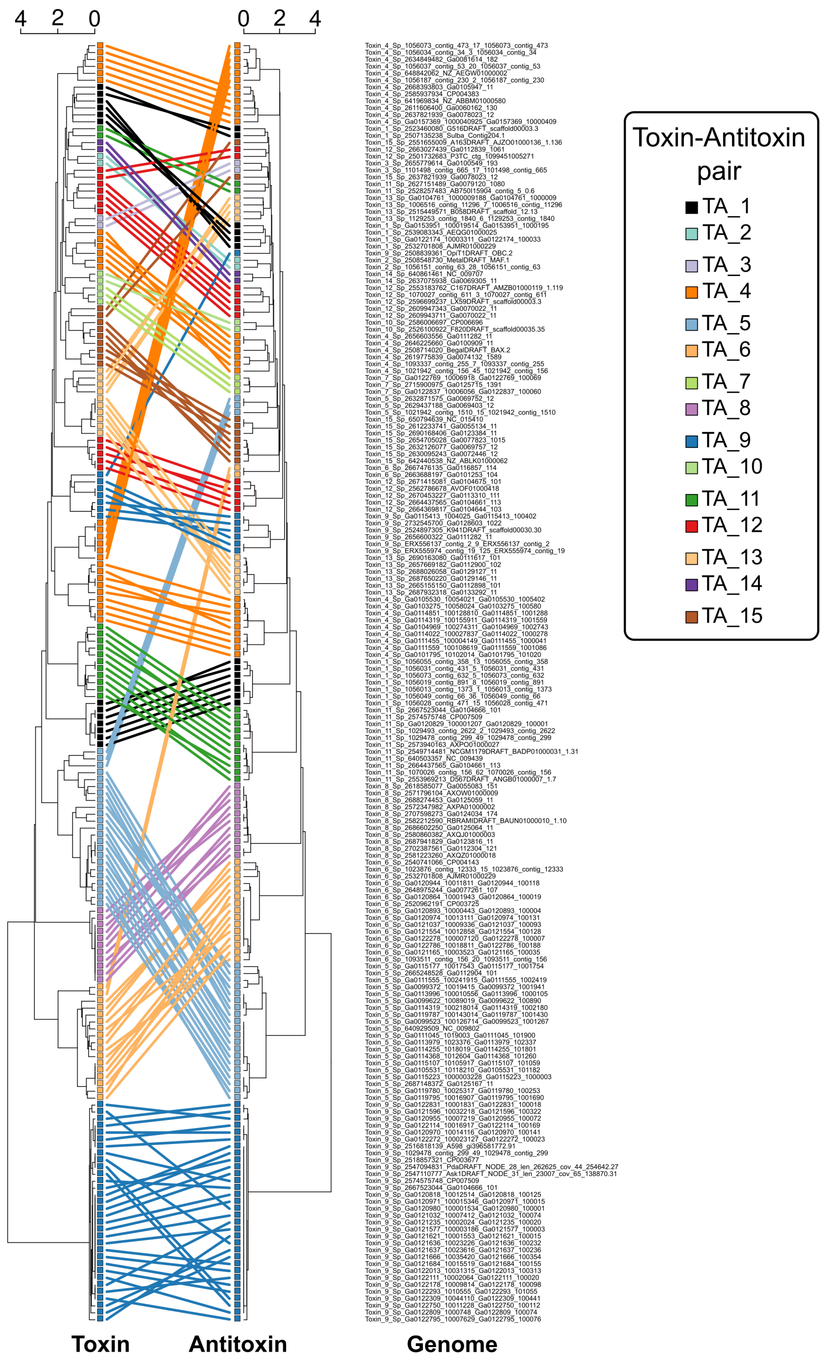

Supplementary Figure 9. Comparison of predicted toxin and antitoxin proteins similarities. Sequences predicted as toxins and antitoxins were compared using Sequence Demarcation Tool (SDT)[27], and the resulting AAI matrix was used to cluster sequences (UPGMA clustering). Predicted toxin-antitoxin (TA) pairs are highlighted with colors. The corresponding genome of the system is indicated at the bottom in the same order as the antitoxin gene.

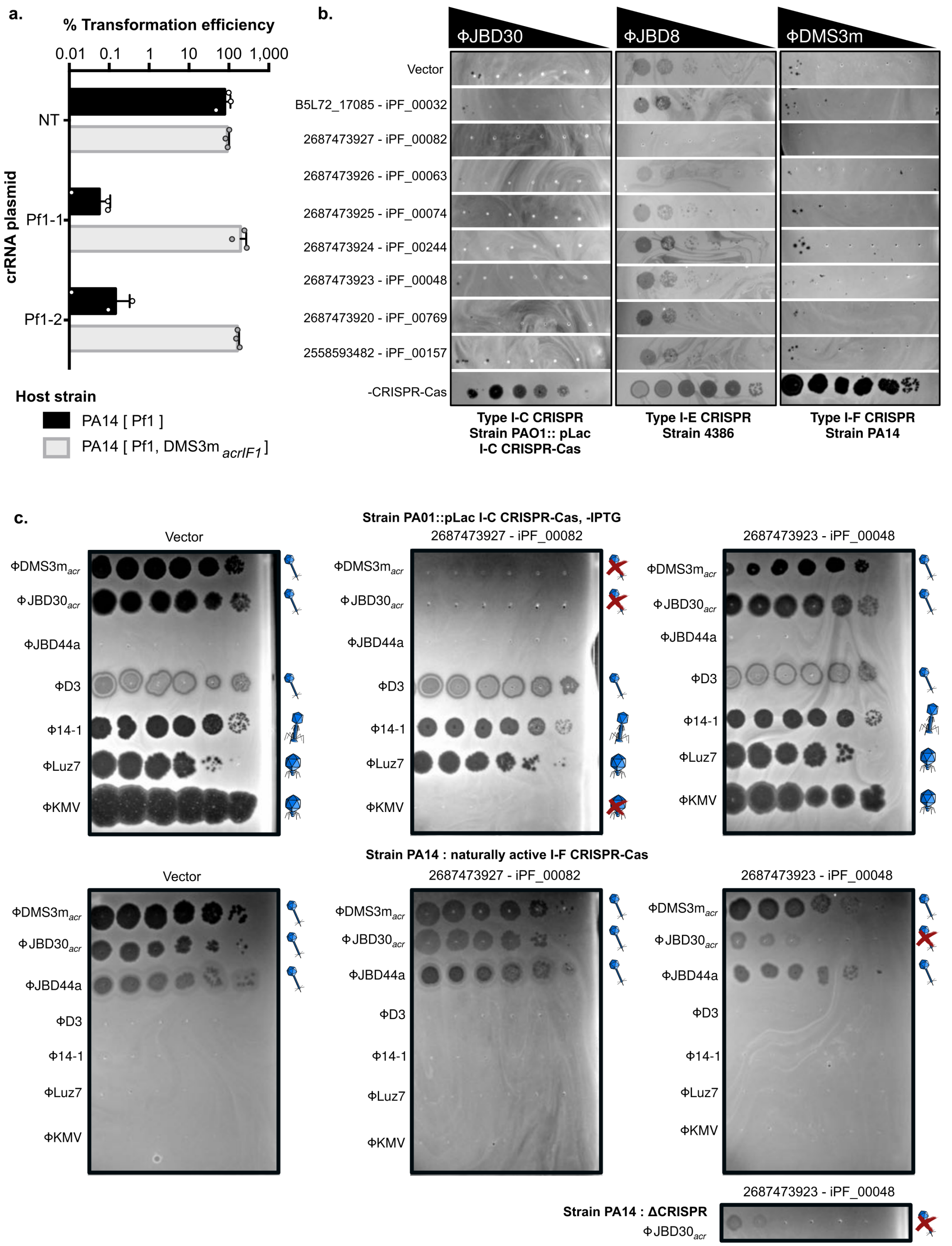

Supplementary Figure 10. Evaluation of self-targeting lethality, trans-acting anti-CRISPR activity from co-infecting prophages, and anti-CRISPR/superinfection activity of uncharacterized genes predicted on inovirus prophages in *Pseudomonas aeruginosa*. A. Transformation assay to evaluate viability of cells including a self-targeted inovirus in the

presence and absence of a co-infecting acr-encoding prophage. Percent transformation efficiency of crispr RNA (crRNA)-expressing plasmids were calculated relative to an empty vector, in *Pseudomonas aeruginosa* strains PA14 naturally lysogenized with inovirus Pf1 (PA14 [Pf1]) or dual lysogenized with Pf1 and acr-expressing siphovirus DMS3m$_{acrIF1}$ (PA14 [Pf1 , DMS3m$_{\mathrm{acrIF1}}$]). NT = non-targeting crRNA, Pf1-1 and Pf1-2 crRNAs target the coat protein gene in inovirus Pf1. For each condition, bars represent the average value of biological triplicates, and error bars represent the standard deviation across triplicates. B. Phage plaque assay to assess anti-CRISPR activity of candidate genes, using 3 host strains (left, middle, and right panel) each expressing a different type of CRISPR-Cas system, and the corresponding targeted phages (indicated on top of each panel). Host strains 4386 and PA14 encode a naturally active Type I-E and Type I-F CRISPR-Cas system (respectively), while strain PAO1 encodes Type I-C Cas genes integrated under the control of an IPTG inducible promoter, in presence of IPTG. Ten-fold serial dilutions of the targeted phages were titered on lawns of *Pseudomonas aeruginosa* expressing the empty vector (top row), a candidate gene (rows 2 to 11), or with CRISPR immunity suppressed (bottom row, condition -CRISPR-Cas). C. Phage plaque assays illustrating superinfection exclusion properties of genes 2687473927 (middle panel) and 2687473923 (right panel), relative to vector control (left panel). Serial dilutions (from left to right) of a set of phages (rows 1 to 7 in each picture) were spotted onto lawn cultures of strain PAO1 with the I-C Cas genes integrated under the control of an IPTG inducible promoter in the absence of IPTG (top), or of strain PA14 (bottom). Interpretation of infection outcome is indicated to the right of each lane, with successful infection represented by a phage symbol, and superinfection exclusion represented by a phage symbol barred by a red cross. To confirm that the inhibitory phenotype of 2687473923 on phage JBD30 and host PA14 is CRISPR-independent, the assay was repeated in a strain of PA14 lacking an active Type I-F system (PA14 ΔCRISPR, bottom right). The full antiCRISPR experiment was conducted once, while all superinfection experiments were conducted twice and produced similar results.

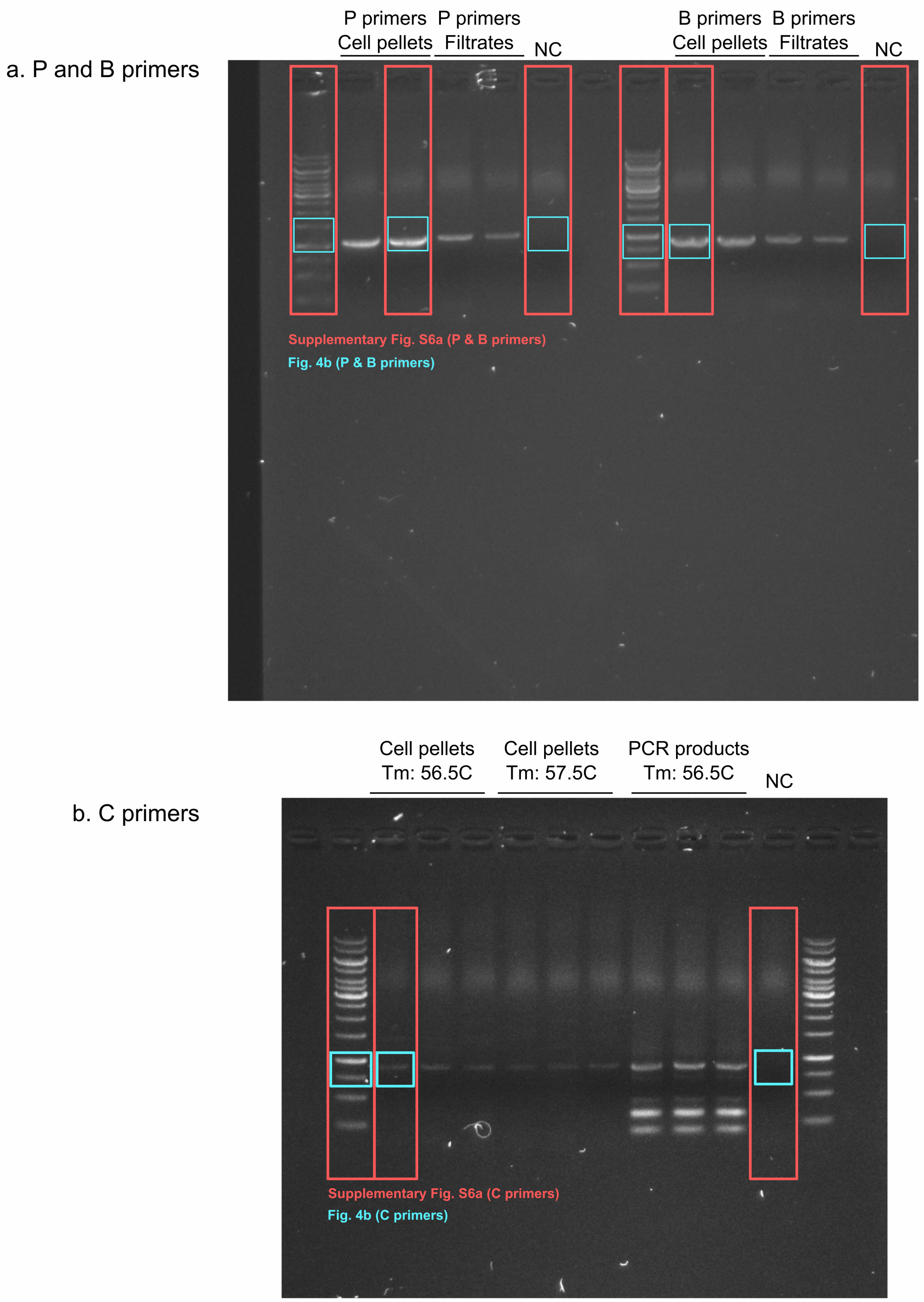

Supplementary Figure 11. Full size gels from Fig. 4 (panel b) and Supplementary Fig. 6. P primers: PCR Primers amplifying across the predicted provirus integration site. B primers: PCR primers internal to the predicted provirus, amplifying both the integrated and circularized form. C primers: PCR primers spanning across the predicted attachment site and amplifying only the circularized form. Tm: melting temperature. NC: No template control. The sections cropped and displayed in Fig. 4 and Supplementary Fig. 6 are highlighted in blue and red, respectively.

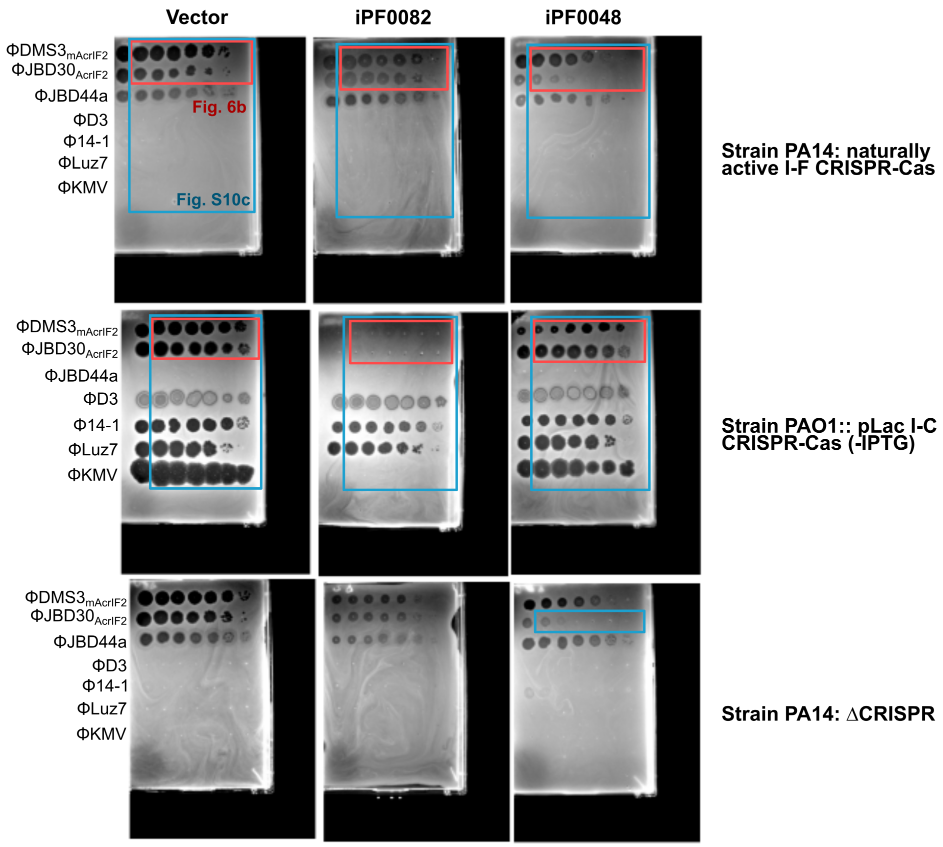

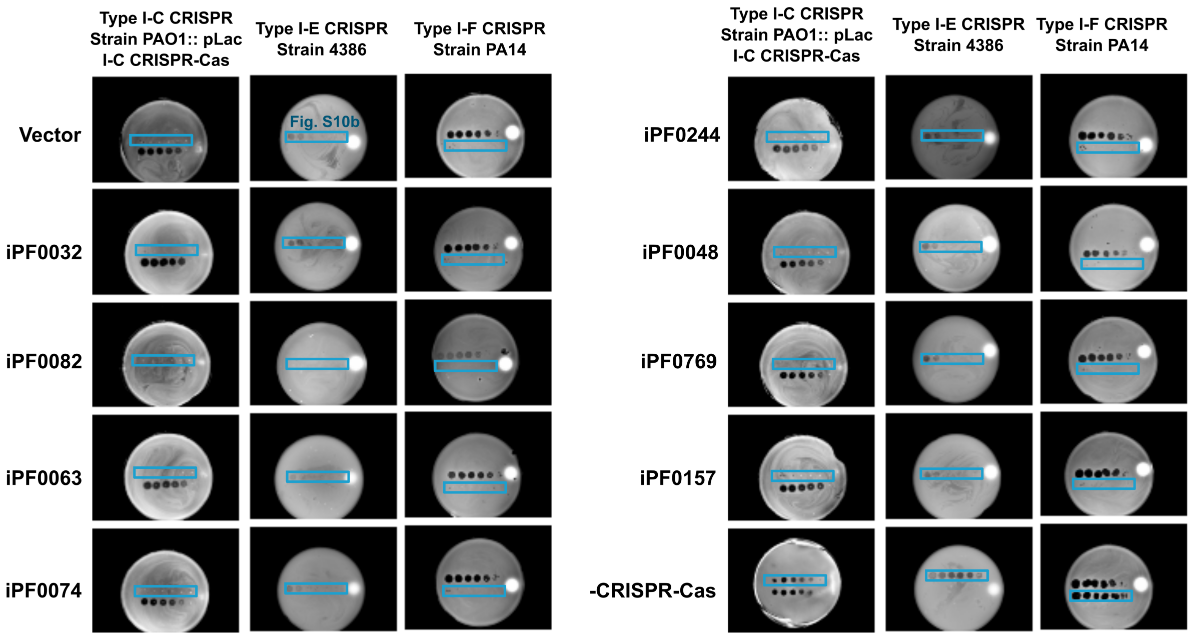

Supplementary Figure 12. Full size gels from Fig. 6b and Supplementary Fig. 10. The sections cropped and displayed in Fig. 6 and Supplementary Fig. 10 are highlighted in red and blue, respectively.

Supplementary Tables

Supplementary Table 1. List and characteristics of reference inovirus genomes used in this study. For each genome, genome features (size and type), ICTV classification, and known or predicted major coat proteins are indicated. Proteins that were not annotated as major coat but only predicted based on protein size and the presence of a single transmembrane domain (TMD) are highlighted in yellow. The tab "Structural protein detections" includes the detection of all putative structural proteins (i.e. major and minor coat proteins) in the same reference genomes. TMD: transmembrane domain.

Supplementary Table 2. List of genomes and metagenomes mined. Genomes are associated with their IMG identifiers and taxonomic affiliation, with amendment to this affiliation specifically for the inovirus-encoding contigs added in the "Notes" column. Metagenomes are associated with their GOLD biome classifications, as well as the summarized ecosystem categories and subcategories used for Fig. 2. For genomes and metagenomes in which inoviruses were detected, the associated project name, dataset name, PI, and publication information (if available) are indicated in the tab "Inovirus distribution across datasets", based on information available in the GOLD database.

Supplementary Table 3. Classification of inovirus sequences into species, proposed families, and proposed subfamilies. Putative tandem detections, i.e. neighboring inovirus prophages for which clear boundaries could not be identified, are shown in a separate tab ("Tandems") and were not included in the network from which the family/subfamily classification was derived. Each sequence is associated with its host genome affiliation or the sample ecosystem classification of the metagenome it was assembled from.

Supplementary Table 4. Additional indication of inovirus infection for 20 phylum-level putative host groups. Since inovirus sequences have only been detected in a (draft) genome for these groups, they could potentially originate from genome contamination, either physical sample contamination or *in silico* contamination for metagenome-assembled genomes. Two indicators were used to confirm the host linkage and alleviate this potential contamination: the presence of an integrated inovirus in a large host contig with confident affiliation, and the presence of match(es) between CRISPR spacer(s) and predicted inovirus sequence(s). These examples are listed here for each group highlighted in bold in Fig. 3.

Supplementary Table 5. Functional annotation of protein families (iPFs). Protein sequences were affiliated against the PFAM database and reference protein clusters derived from isolate inoviruses (affiliations starting with "PC_"). In the absence of significant hits to PFAM or the reference inovirus protein clusters, protein sequences predicted as putative structural proteins based on sequence characteristics were affiliated as "Predicted_structural", "Predicted_structural_SP", or "Putative_structural" depending on the prediction confidence (see

Supplementary Table 1, tab "Structural proteins detections"). iPFs were then organized in a two levels functional classification (columns 3 and 4). Identification of motifs for replication and integration iPFs as well as toxin-antitoxin pair iPFs are shown in separate tabs. Conserved domains were identified in iPFs affiliated as replication initiation and integration proteins, except for cases where too few sequences were available to reliably identify motifs (identified with "-"). Putative toxin-antitoxin are identified as pairs of co-occurring iPFs systematically located next to each other in inovirus genomes and for which at least one member of the pair was affiliated as either a putative toxin or antitoxin.

Supplementary Table 6. List of matches between inovirus sequences and IMG CRISPR spacer database. Only cases with 0 or 1 mismatch between the spacer and putative viral sequences are included. Characteristics of host genomes with inovirus self-target, i.e. CRISPR spacer maching an integrated inovirus prophage in the same genome, are indicated in a separate tab. For each match, the prophage and spacer ID is indicated, along with the list of putative anti-CRISPR proteins, the detection of non-inovirus prophages in the same genomes (VirSorter predictions and identification of large terminase subunit), and the number of uncharacterized proteins with an HTH domain identified in the inovirus genome (using the representative genome from the inovirus species).

Supplementary References

1. Rosvall, M. & Bergstrom, C. T. Multilevel compression of random walks on networks reveals hierarchical organization in large integrated systems. *PLoS One* **6,** (2011).
2. Remmert, M., Biegert, A., Hauser, A. & Söding, J. HHblits: lightning-fast iterative protein sequence searching by HMM-HMM alignment. *Nat. Methods* **9,** 173–175 (2011).
3. Li, W. & Godzik, A. Cd-hit: A fast program for clustering and comparing large sets of protein or nucleotide sequences. *Bioinformatics* **22,** 1658–1659 (2006).
4. Edgar, R. C. MUSCLE: a multiple sequence alignment method with reduced time and space complexity. *BMC Bioinformatics* **5,** 113 (2004).
5. Eddy, S. R. Accelerated Profile HMM Searches. *PLoS Comput. Biol.* **7,** e1002195 (2011).
6. Fouts, D. E. Phage_Finder: automated identification and classification of prophage regions in complete bacterial genome sequences. *Nucleic Acids Res.* **34,** 5839–51 (2006).
7. Akhter, S., Aziz, R. K. & Edwards, R. A. PhiSpy: a novel algorithm for finding prophages in bacterial genomes that combines similarity- and composition-based strategies. *Nucleic Acids Res.* **40,** 1–13 (2012).
8. Amgarten, D., Braga, L. P. P., da Silva, A. M. & Setubal, J. C. MARVEL, a Tool for Prediction of Bacteriophage Sequences in Metagenomic Bins. *Front. Genet.* **9,** 1–8 (2018).
9. Davis, B. M., Moyer, K. E., Fidelma Boyd, E. & Waldor, M. K. CTX prophages in classical biotype Vibrio cholerae: Functional phage genes but dysfunctional phage genomes. *J. Bacteriol.* **182,** 6992–6998 (2000).
10. Bille, E. *et al.* A virulence-associated filamentous bacteriophage of Neisseria meningitidis increases host-cell colonisation. *PLoS Pathog.* **13,** 1–23 (2017).
11. Ku, C., Lo, W. S., Chen, L. L. & Kuo, C. H. Complete genomes of two dipteran-associated spiroplasmas provided insights into the origin, dynamics, and impacts of viral invasion in Spiroplasma. *Genome Biol. Evol.* **5,** 1151–1164 (2013).
12. Roux, S., Hallam, S. J., Woyke, T. & Sullivan, M. B. Viral dark matter and virus-host interactions resolved from publicly available microbial genomes. *Elife* **4,** e08490 (2015).
13. Díaz-Muñoz, S. L., Sanjuán, R. & West, S. Sociovirology: Conflict, Cooperation, and Communication among Viruses. *Cell Host Microbe* **22,** 437–441 (2017).

14. Adriaenssens, E. M., Krupovic, M. & Knezevic, P. Taxonomy of prokaryotic viruses : 2016 update from the ICTV bacterial and archaeal viruses subcommittee. *Arch. Virol.* **162,** 1153–1157 (2017).

15. Iranzo, J., Krupovic, M. & Koonin, E. V. The double-stranded DNA virosphere as a modular hierarchical network of gene sharing. *MBio* **7,** e00978-16 (2016).

16. Wu, E. *et al.* Characterization of a cryptic plasmid from Bacillus sphaericus strain LP1-G. *Plasmid* **57,** 296–305 (2007).

17. Ilyina, T.V. ; Koonin, E. V., Ilyina, T. V & Koonin, E. V. Conserved sequence motifs in the initiator proteins for rolling circle DNA replication encoded by diverse replicaons from eubacteria, eucaryotes and archaeabacteria. *Nucleic Acids Res.* **20,** 3279–3285 (1992).

18. Kimura, M., Wang, G., Nakayama, N. & Asakawa, S. in *Biocommunication in Soil Microorganisms.* (ed. Witzany, G.) 189–213 (Springer Berlin Heidelberg, 2011).

19. Wang, Y. *et al.* Identification, characterization, and application of the replicon region of the halophilic temperate sphaerolipovirus SNJ1. *J. Bacteriol.* **198,** 1952–1964 (2016).

20. Krupovic, M., Cvirkaite-Krupovic, V., Iranzo, J., Prangishvili, D. & Koonin, E. V. Viruses of archaea: Structural, functional, environmental and evolutionary genomics. *Virus Res.* **244,** 181–193 (2018).

21. Mochimaru, H. *et al.* Methanolobus profundi sp. nov., a methylotrophic methanogen isolated from deep subsurface sediments in a natural gas field. *Int. J. Syst. Evol. Microbiol.* **59,** 714–718 (2009).

22. Edwards, R. A., McNair, K., Faust, K., Raes, J. & Dutilh, B. E. Computational approaches to predict bacteriophage-host relationships. *FEMS Microbiol. Rev.* **40,** 258–272 (2016).

23. Kim, A. Y. & Blaschek, H. P. Construction and characterization of a phage-plasmid hybrid (phagemid), pCAK1, containing the replicative form of viruslike particle CAK1 isolated from Clostridium acetobutylicum NCIB 6444. *J. Bacteriol.* **175,** 3838–3843 (1993).

24. Bondy-Denomy, J. *et al.* Prophages mediate defense against phage infection through diverse mechanisms. *ISME J.* **22,** 1–13 (2016).

25. Gupta, R. S. Origin of diderm (Gram-negative) bacteria: Antibiotic selection pressure rather than endosymbiosis likely led to the evolution of bacterial cells with two membranes. *Antonie van Leeuwenhoek, Int. J. Gen. Mol. Microbiol.* **100,** 171–182 (2011).

26. Kelley, L. A., Mezulis, S., Yates, C., Wass, M. & Sternberg, M. The Phyre2 web portal for protein modelling, prediction, and analysis. *Nat. Protoc.* **10,** 845–858 (2015).

27. Muhire, B. M., Varsani, A. & Martin, D. P. SDT: A virus classification tool based on pairwise sequence alignment and identity calculation. *PLoS One* **9,** (2014).

28. Pester, M *et al.* Complete genome sequences of Desulfosporosinus orientis DSM765T, Desulfosporosinus youngiae DSM17734T, Desulfosporosinus meridiei DSM13257T, and Desulfosporosinus acidiphilus DSM22704T. *J. Bacteriol.* **194,** 6300-1 (2012).

29. Stolze, Y *et al.* Identification and genome reconstruction of abundant distinct taxa in microbiomes from one thermophilic and three mesophilic production-scale biogas plants. *Biotechnol Biofuels.* **9,** 156 (2016).

30. Rossmassler, K *et al.* Metagenomic analysis of the microbiota in the highly compartmented hindguts of six wood- or soil-feeding higher termites. *Microbiome.* **3,** 56 (2015).

31. Brzoska, RM. Bollmann, A. The long-term effect of uranium and pH on the community composition of an artificial consortium. *FEMS Microbiol. Ecol.* **92,** (2016).

32. Sorensen, JW. Dunivin, TK. Tobin, TC. Shade, A. Ecological selection for small microbial genomes along a temperate-to-thermal soil gradient. *Nat Microbiol.* **4,** 55-61 (2019).

33. Caro-Quintero, A *et al.* Genome sequencing of five Shewanella baltica strains recovered from the oxic-anoxic interface of the Baltic Sea. *J. Bacteriol.* **194,** 1236 (2012).

34. Maresca, JA. Miller, KJ. Keffer, JL. Sabanayagam, CR. Campbell, BJ. Distribution and Diversity of Rhodopsin-Producing Microbes in the Chesapeake Bay. *Appl. Environ. Microbiol.* **84,** (2018).

35. Shetty, AR *et al.* Complete genome sequence of the phenanthrene-degrading soil bacterium Delftia acidovorans Cs1-4. *Stand Genomic Sci.* **10,** 55 (2015).

36. Seitz, KW. Lazar, CS. Hinrichs, KU. Teske, AP. Baker, BJ. Genomic reconstruction of a novel, deeply branched sediment archaeal phylum with pathways for acetogenesis and sulfur reduction. *ISME J.* **10,** 1696-705 (2016).

37. Auerbach, RK *et al.* Yersinia pestis evolution on a small timescale: comparison of whole genome sequences from North America. *PLoS ONE.* **2,** e770 (2007).

38. Chen, J *et al.* Whole genome sequences of two Xylella fastidiosa strains (M12 and M23) causing almond leaf scorch disease in California. *J. Bacteriol.* **192,** 4534 (2010).

39. Tuanyok, A *et al.* A horizontal gene transfer event defines two distinct groups within Burkholderia pseudomallei that have dissimilar geographic distributions. *J. Bacteriol.* **189,** 9044-9 (2007).

40. Peacock, JP *et al.* Pyrosequencing reveals high-temperature cellulolytic microbial consortia in Great Boiling Spring after in situ lignocellulose enrichment. *PLoS ONE.* **8,** e59927 (2013).

41. Hedlund, BP *et al.* High-Quality Draft Genome Sequence of Kallotenue papyrolyticum JKG1T Reveals Broad Heterotrophic Capacity Focused on Carbohydrate and Amino Acid Metabolism. *Genome Announc.* **3,** (2015).

42. Carlos, C. Fan, H. Currie, CR. Substrate Shift Reveals Roles for Members of Bacterial Consortia in Degradation of Plant Cell Wall Polymers. *Front Microbiol.* **9,** 364 (2018).

43. Gontang, EA *et al.* Major changes in microbial diversity and community composition across gut sections of a juvenile Panchlora cockroach. *PLoS ONE.* **12,** e0177189 (2017).

44. Angle, JC *et al.* Methanogenesis in oxygenated soils is a substantial fraction of wetland methane emissions. *Nat Commun.* **8,** 1567 (2017).

45. Blair, PM *et al.* Exploration of the Biosynthetic Potential of the *Populus* Microbiome. *mSystems.* **3,** ().

46. Burdman, S. Walcott, R. Acidovorax citrulli: generating basic and applied knowledge to tackle a global threat to the cucurbit industry. *Mol. Plant Pathol.* **13,** 805-15 (2012).

47. Baltrus, DA *et al.* Absence of genome reduction in diverse, facultative endohyphal bacteria. *Microb Genom.* **3,** e000101 (2017).

48. Ziels, RM. Sousa, DZ. Stensel, HD. Beck, DAC. DNA-SIP based genome-centric metagenomics identifies key long-chain fatty acid-degrading populations in anaerobic digesters with different feeding frequencies. *ISME J.* **12,** 112-123 (2018).

49. Weiss, M *et al.* Permanent draft genome sequence of Comamonas testosteroni KF-1. *Stand Genomic Sci.* **8,** 239-54 (2013).

50. Tran, P *et al.* Microbial life under ice: Metagenome diversity and in situ activity of Verrucomicrobia in seasonally ice-covered Lakes. *Environ. Microbiol.* **20,** 2568-2584 (2018).

51. Colatriano, D *et al.* Genomic evidence for the degradation of terrestrial organic matter by pelagic Arctic Ocean Chloroflexi bacteria. *Commun Biol.* **1,** 90 (2018).

52. Graham, EB *et al.* Oligotrophic wetland sediments susceptible to shifts in microbiomes and mercury cycling with dissolved organic matter addition. *PeerJ.* **6,** e4575 (2018).

53. Thiel, V *et al.* The Dark Side of the Mushroom Spring Microbial Mat: Life in the Shadow of Chlorophototrophs. I. Microbial Diversity Based on 16S rRNA Gene Amplicons and Metagenomic Sequencing. *Front Microbiol.* **7,** 919 (2016).

54. Tringe, SG *et al.* Comparative metagenomics of microbial communities. *Science.* **308,** 554-7 (2005).

55. Mende, DR *et al.* Environmental drivers of a microbial genomic transition zone in the ocean's interior. *Nat Microbiol.* **2,** 1367-1373 (2017).

56. Hug, LA. Beiko, RG. Rowe, AR. Richardson, RE. Edwards, EA. Comparative metagenomics of three Dehalococcoides-containing enrichment cultures: the role of the non-dechlorinating community. *BMC Genomics.* **13,** 327 (2012).

57. Nunes da Rocha, U *et al.* Isolation of a significant fraction of non-phototroph diversity from a desert Biological Soil Crust. *Front Microbiol.* **6,** 277 (2015).

58. Brisson, VL *et al.* Metagenomic analysis of a stable trichloroethene-degrading microbial community. *ISME J.* **6,** 1702-14 (2012).

59. Wilhelm, RC. Hanson, BT. Chandra, S. Madsen, E. Community dynamics and functional characteristics of naphthalene-degrading populations in contaminated surface sediments and hypoxic/anoxic groundwater. *Environ. Microbiol.* **20,** 3543-3559 (2018).

60. Aylward, FO *et al.* Convergent bacterial microbiotas in the fungal agricultural systems of insects. *MBio.* **5,** e02077 (2014).

61. Anantharaman, K *et al.* Sulfur oxidation genes in diverse deep-sea viruses. *Science.* **344,** 757-60 (2014).

62. Teeling, H *et al.* Recurring patterns in bacterioplankton dynamics during coastal spring algae blooms. *Elife.* **5,** e11888 (2016).

63. Beller, HR *et al.* Discovery of enzymes for toluene synthesis from anoxic microbial communities. *Nat. Chem. Biol.* **14,** 451-457 (2018).

64. Espínola, F *et al.* Metagenomic Analysis of Subtidal Sediments from Polar and Subpolar Coastal Environments Highlights the Relevance of Anaerobic Hydrocarbon Degradation Processes. *Microb. Ecol.* **75,** 123-139 (2018).

65. Hamilton, TL. Jones, DS. Schaperdoth, I. Macalady, JL. Metagenomic insights into S(0) precipitation in a terrestrial subsurface lithoautotrophic ecosystem. *Front Microbiol.* **5,** 756 (2014).

66. Rusch, DB *et al.* The Sorcerer II Global Ocean Sampling expedition: northwest Atlantic through eastern tropical Pacific. *PLoS Biol.* **5,** e77 (2007).

67. Lee, LL *et al.* Genus-Wide Assessment of Lignocellulose Utilization in the Extremely Thermophilic Genus Caldicellulosiruptor by Genomic, Pangenomic, and Metagenomic Analyses. *Appl. Environ. Microbiol.* **84,** (2018).

68. Graham, EB *et al.* Multi 'omics comparison reveals metabolome biochemistry, not microbiome composition or gene expression, corresponds to elevated biogeochemical function in the hyporheic zone. *Sci. Total Environ.* **642,** 742-753 (2018).

69. Coenye, T *et al.* Burkholderia ambifaria sp. nov., a novel member of the Burkholderia cepacia complex including biocontrol and cystic fibrosis-related isolates. *Int. J. Syst. Evol. Microbiol.* **51,** 1481-90 (2001).

70. Kim, SH *et al.* Genome sequence of Desulfitobacterium hafniense DCB-2, a Gram-positive anaerobe capable of dehalogenation and metal reduction. *BMC Microbiol.* **12,** 21 (2012).

71. Reddy, AP *et al.* Discovery of microorganisms and enzymes involved in high-solids decomposition of rice straw using metagenomic analyses. *PLoS ONE.* **8,** e77985 (2013).

72. Wu, YW *et al.* Ionic Liquids Impact the Bioenergy Feedstock-Degrading Microbiome and Transcription of Enzymes Relevant to Polysaccharide Hydrolysis. *mSystems.* **1,** (2016).

73. Levy, A *et al.* Genomic features of bacterial adaptation to plants. *Nat. Genet.* **50,** 138-150 (2018).

74. Probst, AJ *et al.* Differential depth distribution of microbial function and putative symbionts through sediment-hosted aquifers in the deep terrestrial subsurface. *Nat Microbiol.* **3,** 328-336 (2018).

75. Diamond *et al.*. Processing of grassland soil C-N compounds into soluble and volatile molecules is depth stratified and mediated by genomically novel bacteria and archaea. *Nat Microbiol*. **in press** (2019).

76. Butterfield, CN *et al.* Proteogenomic analyses indicate bacterial methylotrophy and archaeal heterotrophy are prevalent below the grass root zone. *PeerJ.* **4,** e2687 (2016).

77. Brown, CT *et al.* Unusual biology across a group comprising more than 15% of domain Bacteria. *Nature.* **523,** 208-11 (2015).

78. Anantharaman, K *et al.* Thousands of microbial genomes shed light on interconnected biogeochemical processes in an aquifer system. *Nat Commun.* **7,** 13219 (2016).

79. Hug, LA *et al.* Community genomic analyses constrain the distribution of metabolic traits across the Chloroflexi phylum and indicate roles in sediment carbon cycling. *Microbiome.* **1,** 22 (2013).

80. Hug, LA *et al.* Aquifer environment selects for microbial species cohorts in sediment and groundwater. *ISME J.* **9,** 1846-56 (2015).

81. Sharon, I *et al.* Accurate, multi-kb reads resolve complex populations and detect rare microorganisms. *Genome Res.* **25,** 534-43 (2015).

82. Kantor, RS *et al.* Bioreactor microbial ecosystems for thiocyanate and cyanide degradation unravelled with genome-resolved metagenomics. *Environ. Microbiol.* **17,** 4929-41 (2015).

83. Caro-Quintero, A *et al.* Unprecedented levels of horizontal gene transfer among spatially co-occurring Shewanella bacteria from the Baltic Sea. *ISME J.* **5,** 131-40 (2011).

84. Lindemann, SR *et al.* The epsomitic phototrophic microbial mat of Hot Lake, Washington: community structural responses to seasonal cycling. *Front Microbiol.* **4,** 323 (2013).

85. Byrne-Bailey, KG. Coates, JD. Complete genome sequence of the anaerobic perchlorate-reducing bacterium Azospira suillum strain PS. *J. Bacteriol.* **194,** 2767-8 (2012).

86. Byrne-Bailey, KG *et al.* Completed genome sequence of the anaerobic iron-oxidizing bacterium Acidovorax ebreus strain TPSY. *J. Bacteriol.* **192,** 1475-6 (2010).

87. Byrne-Bailey, KG. Weber, KA. Coates, JD. Draft genome sequence of the anaerobic, nitrate-dependent, Fe(II)-oxidizing bacterium Pseudogulbenkiania ferrooxidans strain 2002. *J. Bacteriol.* **194,** 2400-1 (2012).

88. Djao, OD *et al.* Complete genome sequence of Syntrophothermus lipocalidus type strain (TGB-C1). *Stand Genomic Sci.* **3,** 268-75 (2010).

89. Pagani, I *et al.* Complete genome sequence of Desulfobulbus propionicus type strain (1pr3). *Stand Genomic Sci.* **4,** 100-10 (2011).

90. Isanapong, J *et al.* High-quality draft genome sequence of the Opitutaceae bacterium strain TAV1, a symbiont of the wood-feeding termite Reticulitermes flavipes. *J. Bacteriol.* **194,** 2744-5 (2012).

91. Chua, MJ. Campen, RL. Wahl, L. Grzymski, JJ. Mikucki, JA. Genomic and physiological characterization and description of Marinobacter gelidimuriae sp. nov., a psychrophilic, moderate halophile from Blood Falls, an antarctic subglacial brine. *FEMS Microbiol. Ecol.* **94,** (2018).

92. Hesse, C *et al.* Genome-based evolutionary history of Pseudomonas spp. *Environ. Microbiol.* **20,** 2142-2159 (2018).

93. Sergeant, MJ *et al.* Extensive microbial and functional diversity within the chicken cecal microbiome. *PLoS ONE.* **9,** e91941 (2014).

94. Handley, KM *et al.* Biostimulation induces syntrophic interactions that impact C, S and N cycling in a sediment microbial community. *ISME J.* **7,** 800-16 (2013).

95. Bendall, ML *et al.* Genome-wide selective sweeps and gene-specific sweeps in natural bacterial populations. *ISME J.* **10,** 1589-601 (2016).

96. He, S *et al.* Metatranscriptomic array analysis of 'Candidatus Accumulibacter phosphatis'-enriched enhanced biological phosphorus removal sludge. *Environ. Microbiol.* **12,** 1205-17 (2010).

97. Scott, KM *et al.* Genomes of ubiquitous marine and hypersaline Hydrogenovibrio, Thiomicrorhabdus and Thiomicrospira spp. encode a diversity of mechanisms to sustain chemolithoautotrophy in heterogeneous environments. *Environ. Microbiol.* **20,** 2686-2708 (2018).

98. Daly, RA *et al.* Viruses control dominant bacteria colonizing the terrestrial deep biosphere after hydraulic fracturing. *Nat Microbiol.* **4,** 352-361 (2019).

99. Smith, GJ *et al.* Members of the Genus *Methylobacter* Are Inferred To Account for the Majority of Aerobic Methane Oxidation in Oxic Soils from a Freshwater Wetland. *MBio.* **9,** (2018).

100. Pold, G *et al.* Genome Sequence of *Verrucomicrobium* sp. Strain GAS474, a Novel Bacterium Isolated from Soil. *Genome Announc.* **6,** (2018).

101. Coleman-Derr, D *et al.* Plant compartment and biogeography affect microbiome composition in cultivated and native Agave species. *New Phytol.* **209,** 798-811 (2016).

102. Fonseca-García, C *et al.* The Cacti Microbiome: Interplay between Habitat-Filtering and Host-Specificity. *Front Microbiol.* **7,** 150 (2016).

103. Chistoserdova, L *et al.* Genome of Methylobacillus flagellatus, molecular basis for obligate methylotrophy, and polyphyletic origin of methylotrophy. *J. Bacteriol.* **189,** 4020-7 (2007).

104. Kits, KD *et al.* Genome Sequence of the Obligate Gammaproteobacterial Methanotroph Methylomicrobium album Strain BG8. *Genome Announc.* **1,** e0017013 (2013).

105. Svenning, MM *et al.* Genome sequence of the Arctic methanotroph Methylobacter tundripaludum SV96. *J. Bacteriol.* **193,** 6418-9 (2011).

106. Boden, R *et al.* Complete genome sequence of the aerobic marine methanotroph Methylomonas methanica MC09. *J. Bacteriol.* **193,** 7001-2 (2011).

107. Lapidus, A *et al.* Genomes of three methylotrophs from a single niche reveal the genetic and metabolic divergence of the methylophilaceae. *J. Bacteriol.* **193,** 3757-64 (2011).

108. Kalyuzhnaya, MG *et al.* Draft genome sequences of gammaproteobacterial methanotrophs isolated from lake washington sediment. *Genome Announc.* **3,** (2015).

109. Hamilton, R *et al.* Draft genomes of gammaproteobacterial methanotrophs isolated from terrestrial ecosystems. *Genome Announc.* **3,** (2015).

110. Frindte, K *et al.* Draft Genome Sequences of Two Gammaproteobacterial Methanotrophs Isolated from Rice Ecosystems. *Genome Announc.* **5,** (2017).

111. Narayan, KD. Badhai, J. Whitman, WB. Das, SK. Draft Genome Sequence of Comamonas thiooxydans Strain S23T (DSM 17888T), a Thiosulfate-Oxidizing Bacterium Isolated from a Sulfur Spring in India. *Genome Announc.* **4,** (2016).

112. Roux, S *et al.* Ecogenomics and potential biogeochemical impacts of globally abundant ocean viruses. *Nature.* **537,** 689-693 (2016).

113. Hawley, ER *et al.* Metagenomes from two microbial consortia associated with Santa Barbara seep oil. *Mar Genomics.* **18 Pt B,** 97-9 (2014).

114. Hess, M *et al.* Metagenomic discovery of biomass-degrading genes and genomes from cow rumen. *Science.* **331,** 463-7 (2011).

115. Dalcin Martins, P *et al.* Viral and metabolic controls on high rates of microbial sulfur and carbon cycling in wetland ecosystems. *Microbiome.* **6,** 138 (2018).

116. Shiller, AM. Chan, EW. Joung, DJ. Redmond, MC. Kessler, JD. Light rare earth element depletion during Deepwater Horizon blowout methanotrophy. *Sci Rep.* **7,** 10389 (2017).

117. Strous, M. Kraft, B. Bisdorf, R. Tegetmeyer, HE. The binning of metagenomic contigs for microbial physiology of mixed cultures. *Front Microbiol.* **3,** 410 (2012).

118. Seshadri, R *et al.* Discovery of Novel Plant Interaction Determinants from the Genomes of 163 Root Nodule Bacteria. *Sci Rep.* **5,** 16825 (2015).

119. De Meyer, SE *et al.* High-quality permanent draft genome sequence of the Lebeckia ambigua-nodulating Burkholderia sp. strain WSM4176. *Stand Genomic Sci.* **10,** 79 (2015).

120. Hutt, LP *et al.* Permanent draft genome of *Thiobacillus thioparus* DSM 505^{T}, an obligately chemolithoautotrophic member of the *Betaproteobacteria*. *Stand Genomic Sci.* **12,** 10 (2017).

121. Mukherjee, S *et al.* 1,003 reference genomes of bacterial and archaeal isolates expand coverage of the tree of life. *Nat. Biotechnol.* **35,** 676-683 (2017).

122. Zhou, Y *et al.* High quality draft genome sequence of the slightly halophilic bacterium Halomonas zhanjiangensis type strain JSM 078169(T) (DSM 21076(T)) from a sea urchin in southern China. *Stand Genomic Sci.* **9,** 1020-30 (2014).

123. Satomi, M. Kimura, B. Hamada, T. Harayama, S. Fujii, T. Phylogenetic study of the genus Oceanospirillum based on 16S rRNA and gyrB genes: emended description of the genus

Oceanospirillum, description of Pseudospirillum gen. nov., Oceanobacter gen. nov. and Terasakiella gen. nov. and transfer of Oceanospirillum jannaschii and Pseudomonas stanieri to Marinobacterium as Marinobacterium jannaschii comb. nov. and Marinobacterium stanieri comb. no. *Int. J. Syst. Evol. Microbiol.* **52,** 739-47 (2002).

124. Oulas, A *et al.* Metagenomic investigation of the geologically unique Hellenic Volcanic Arc reveals a distinctive ecosystem with unexpected physiology. *Environ. Microbiol.* **18,** 1122-36 (2016).

125. Spang, A *et al.* Complex archaea that bridge the gap between prokaryotes and eukaryotes. *Nature.* **521,** 173-179 (2015).

126. Dombrowski, N. Teske, AP. Baker, BJ. Expansive microbial metabolic versatility and biodiversity in dynamic Guaymas Basin hydrothermal sediments. *Nat Commun.* **9,** 4999 (2018).

127. Suwa, Y *et al.* Genome sequence of Nitrosomonas sp. strain AL212, an ammonia-oxidizing bacterium sensitive to high levels of ammonia. *J. Bacteriol.* **193,** 5047-8 (2011).

128. Wen, A. Fegan, M. Hayward, C. Chakraborty, S. Sly, LI. Phylogenetic relationships among members of the Comamonadaceae, and description of Delftia acidovorans (den Dooren de Jong 1926 and Tamaoka et al. 1987) gen. nov., comb. nov. *Int. J. Syst. Bacteriol.* **49 Pt 2,** 567-76 (1999).

129. García Martín, H *et al.* Metagenomic analysis of two enhanced biological phosphorus removal (EBPR) sludge communities. *Nat. Biotechnol.* **24,** 1263-9 (2006).

130. Jungbluth, SP. Amend, JP. Rappé, MS. Metagenome sequencing and 98 microbial genomes from Juan de Fuca Ridge flank subsurface fluids. *Sci Data.* **4,** 170037 (2017).

131. Tschitschko, B *et al.* Genomic variation and biogeography of Antarctic haloarchaea. *Microbiome.* **6,** 113 (2018).

132. Bagnoud, A *et al.* Reconstructing a hydrogen-driven microbial metabolic network in Opalinus Clay rock. *Nat Commun.* **7,** 12770 (2016).

133. Janssen, PJ *et al.* The complete genome sequence of Cupriavidus metallidurans strain CH34, a master survivalist in harsh and anthropogenic environments. *PLoS ONE.* **5,** e10433 (2010).

134. Chain, P *et al.* Complete genome sequence of the ammonia-oxidizing bacterium and obligate chemolithoautotroph Nitrosomonas europaea. *J. Bacteriol.* **185,** 2759-73 (2003).

135. Garcia, SL *et al.* Model Communities Hint at Promiscuous Metabolic Linkages between Ubiquitous Free-Living Freshwater Bacteria. *mSphere.* **3,** (2018).

136. Hawley, AK *et al.* A compendium of multi-omic sequence information from the Saanich Inlet water column. *Sci Data.* **4,** 170160 (2017).

137. Roux, S *et al.* Ecology and evolution of viruses infecting uncultivated SUP05 bacteria as revealed by single-cell- and meta-genomics. *Elife.* **3,** e03125 (2014).

138. Kolinko, S *et al.* A bacterial pioneer produces cellulase complexes that persist through community succession. *Nat Microbiol.* **3,** 99-107 (2018).

139. Wu, YW. Tang, YH. Tringe, SG. Simmons, BA. Singer, SW. MaxBin: an automated binning method to recover individual genomes from metagenomes using an expectation-maximization algorithm. *Microbiome.* **2,** 26 (2014).

140. Hiras, J *et al.* Comparative Community Proteomics Demonstrates the Unexpected Importance of Actinobacterial Glycoside Hydrolase Family 12 Protein for Crystalline Cellulose Hydrolysis. *MBio.* **7,** (2016).

141. Hiras, J. Wu, YW. Eichorst, SA. Simmons, BA. Singer, SW. Refining the phylum Chlorobi by resolving the phylogeny and metabolic potential of the representative of a deeply branching, uncultivated lineage. *ISME J.* **10,** 833-45 (2016).

142. Yao, Q *et al.* Community proteogenomics reveals the systemic impact of phosphorus availability on microbial functions in tropical soil. *Nat Ecol Evol.* **2,** 499-509 (2018).

143. Lykidis, A *et al.* Multiple syntrophic interactions in a terephthalate-degrading methanogenic consortium. *ISME J.* **5,** 122-30 (2011).

144. He, S *et al.* Patterns in wetland microbial community composition and functional gene repertoire associated with methane emissions. *MBio.* **6,** e00066-15 (2015).

145. Nasu, H *et al.* A filamentous phage associated with recent pandemic Vibrio parahaemolyticus O3:K6 strains. *J. Clin. Microbiol.* **38,** 2156-61 (2000).

146. Singer, E *et al.* High-resolution phylogenetic microbial community profiling. *ISME J.* **10,** 2020-32 (2016).

147. Sunagawa, S *et al.* Ocean plankton. Structure and function of the global ocean microbiome. *Science.* **348,** 1261359 (2015).

148. Stolz, JF *et al.* Sulfurospirillum barnesii sp. nov. and Sulfurospirillum arsenophilum sp. nov., new members of the Sulfurospirillum clade of the epsilon Proteobacteria. *Int. J. Syst. Bacteriol.* **49 Pt 3,** 1177-80 (1999).

149. Chakraborty, R *et al.* Complete genome sequence of *Pseudomonas stutzeri* strain RCH2 isolated from a Hexavalent Chromium [Cr(VI)] contaminated site. *Stand Genomic Sci.* **12,** 23 (2017).

150. Deangelis, KM *et al.* Metagenomes of tropical soil-derived anaerobic switchgrass-adapted consortia with and without iron. *Stand Genomic Sci.* **7,** 382-98 (2013).

151. Azakami, H *et al.* Isolation and characterization of a plasmid DNA from periodontopathogenic bacterium, Eikenella corrodens 1073, which affects pilus formation and colony morphology. *Gene.* **351,** 143-8 (2005).

152. Whitman, T *et al.* Dynamics of microbial community composition and soil organic carbon mineralization in soil following addition of pyrogenic and fresh organic matter. *ISME J.* **10,** 2918-2930 (2016).

153. van Passel, MW *et al.* Genome sequence of Victivallis vadensis ATCC BAA-548, an anaerobic bacterium from the phylum Lentisphaerae, isolated from the human gastrointestinal tract. *J. Bacteriol.* **193,** 2373-4 (2011).

154. Porcar, M *et al.* Microbial Ecology on Solar Panels in Berkeley, CA, United States. *Front Microbiol.* **9,** 3043 (2018).

155. Seshadri, R *et al.* Cultivation and sequencing of rumen microbiome members from the Hungate1000 Collection. *Nat. Biotechnol.* **36,** 359-367 (2018).

156. Inskeep, WP *et al.* The YNP Metagenome Project: Environmental Parameters Responsible for Microbial Distribution in the Yellowstone Geothermal Ecosystem. *Front Microbiol.* **4,** 67 (2013).

157. Gutierrez, T *et al.* Genome Sequence of Polycyclovorans algicola Strain TG408, an Obligate Polycyclic Aromatic Hydrocarbon-Degrading Bacterium Associated with Marine Eukaryotic Phytoplankton. *Genome Announc.* **3,** (2015).

158. Liu, J. Liu, Q. Shen, P. Huang, YP. Isolation and characterization of a novel filamentous phage from Stenotrophomonas maltophilia. *Arch. Virol.* **157,** 1643-50 (2012).

159. Wu, E *et al.* Characterization of a cryptic plasmid from Bacillus sphaericus strain LP1-G. *Plasmid.* **57,** 296-305 (2007).

160. Wang, T. Sun, B. Yang, Y. Zhao, T. Genome Sequence of Acidovorax citrulli Group 1 Strain pslb65 Causing Bacterial Fruit Blotch of Melons. *Genome Announc.* **3,** (2015).

161. Adams, MD *et al.* Comparative genome sequence analysis of multidrug-resistant Acinetobacter baumannii. *J. Bacteriol.* **190,** 8053-64 (2008).

162. Vallenet, D *et al.* Comparative analysis of Acinetobacters: three genomes for three lifestyles. *PLoS ONE.* **3,** e1805 (2008).

163. Singh, NK. Kumar, S. Raghava, GP. Mayilraj, S. Draft Genome Sequence of Acinetobacter baumannii Strain MSP4-16. *Genome Announc.* **1,** e0013713 (2013).

164. Ho, AY. Chow, KH. Law, PY. Tse, H. Ho, PL. Draft Genome Sequences of Two Multidrug-Resistant Acinetobacter baumannii Strains of Sequence Type ST92 and ST96. *Genome Announc.* **1,** (2013).

165. Sahl, JW *et al.* Genomic comparison of multi-drug resistant invasive and colonizing Acinetobacter baumannii isolated from diverse human body sites reveals genomic plasticity. *BMC Genomics.* **12,** 291 (2011).

166. Barbe, V *et al.* Unique features revealed by the genome sequence of Acinetobacter sp. ADP1, a versatile and naturally transformation competent bacterium. *Nucleic Acids Res.* **32,** 5766-79 (2004).

167. Chen, Y *et al.* Draft genome sequence of an Acinetobacter genomic species 3 strain harboring a bla(NDM-1) gene. *J. Bacteriol.* **194,** 204-5 (2012).

168. Huang, BF. Kropinski, AM. Bujold, AR. MacInnes, JI. Complete genome sequence of Actinobacillus equuli subspecies equuli ATCC 19392(T). *Stand Genomic Sci.* **10,** 32 (2015).

169. Arya, G. Niven, DF. Production of haemolysins by strains of the Actinobacillus minor/"porcitonsillarum" complex. *Vet. Microbiol.* **141,** 332-41 (2010).

170. Wu, CJ *et al.* Genome sequence of a novel human pathogen, Aeromonas aquariorum. *J. Bacteriol.* **194,** 4114-5 (2012).

171. Chan, KG *et al.* Draft Genome Sequence of Aeromonas caviae Strain L12, a Quorum-Sensing Strain Isolated from a Freshwater Lake in Malaysia. *Genome Announc.* **3,** (2015).

172. Han, JE *et al.* Draft Genome Sequence of a Clinical Isolate, Aeromonas hydrophila SNUFPC-A8, from a Moribund Cherry Salmon (Oncorhynchus masou masou). *Genome Announc.* **1,** (2013).

173. Chan, XY. Chua, KH. Puthucheary, SD. Yin, WF. Chan, KG. Draft genome sequence of an Aeromonas sp. strain 159 clinical isolate that shows quorum-sensing activity. *J. Bacteriol.* **194,** 6350 (2012).

174. Bomar, L *et al.* Draft Genome Sequence of Aeromonas veronii Hm21, a Symbiotic Isolate from the Medicinal Leech Digestive Tract. *Genome Announc.* **1,** (2013).

175. Huang, Y *et al.* Comparative genomic hybridization and transcriptome analysis with a pan-genome microarray reveal distinctions between JP2 and non-JP2 genotypes of Aggregatibacter actinomycetemcomitans. *Mol Oral Microbiol.* **28,** 1-17 (2013).

176. Kapley, A *et al.* Genome Sequence of Alcaligenes sp. Strain HPC1271. *Genome Announc.* **1,** (2013).

177. Lai, Q. Shao, Z. Genome sequence of the alkane-degrading bacterium Alcanivorax hongdengensis type strain A-11-3. *J. Bacteriol.* **194,** 6972 (2012).

178. Zhang, H *et al.* Draft Genome Sequence of Alcanivorax sp. Strain KX64203 Isolated from Deep-Sea Sediments of Iheya North, Okinawa Trough. *Genome Announc.* **4,** (2016).

179. Jung, J. Chun, J. Park, W. Genome sequence of extracellular-protease-producing Alishewanella jeotgali isolated from traditional Korean fermented seafood. *J. Bacteriol.* **194,** 2097 (2012).

180. Xia, X *et al.* Draft genomic sequence of a chromate- and sulfate-reducing Alishewanella strain with the ability to bioremediate Cr and Cd contamination. *Stand Genomic Sci.* **11,** 48 (2016).

181. Adam, Z *et al.* Draft Genome Sequence of Arcobacter cibarius Strain LMG21996T, Isolated from Broiler Carcasses. *Genome Announc.* **2,** (2014).

182. Crovadore, J *et al.* Whole-Genome Sequences of Seven Strains of Bacillus cereus Isolated from Foodstuff or Poisoning Incidents. *Genome Announc.* **4,** (2016).

183. Zwick, ME *et al.* Genomic characterization of the Bacillus cereus sensu lato species: backdrop to the evolution of Bacillus anthracis. *Genome Res.* **22,** 1512-24 (2012).

184. Wang, A. Pattemore, J. Ash, G. Williams, A. Hane, J. Draft genome sequence of Bacillus thuringiensis strain DAR 81934, which exhibits molluscicidal activity. *Genome Announc.* **1,** e0017512 (2013).

185. Liu, G *et al.* Complete genome sequence of Bacillus thuringiensis subsp. kurstaki strain HD73. *Genome Announc.* **1,** e0008013 (2013).

186. He, J *et al.* Complete genome sequence of Bacillus thuringiensis subsp. chinensis strain CT-43. *J. Bacteriol.* **193,** 3407-8 (2011).

187. Murawska, E. Fiedoruk, K. Bideshi, DK. Swiecicka, I. Complete genome sequence of Bacillus thuringiensis subsp. thuringiensis strain IS5056, an isolate highly toxic to Trichoplusia ni. *Genome Announc.* **1,** e0010813 (2013).

188. Martínez-Ocampo, F *et al.* Draft Genome Sequence of Burkholderia cenocepacia Strain CEIB S5-2, a Methyl Parathion- and p-Nitrophenol-Degrading Bacterium, Isolated from Agricultural Soils in Morelos, Mexico. *Genome Announc.* **4,** (2016).

189. Martínez-Ocampo, F *et al.* Burkholderia cenocepacia Strain CEIB S5-1, a Rhizosphere-Inhabiting Bacterium with Potential in Bioremediation. *Genome Announc.* **3,** (2015).

190. Lim, J *et al.* Complete genome sequence of Burkholderia glumae BGR1. *J. Bacteriol.* **191,** 3758-9 (2009).

191. Holden, MT *et al.* Genomic plasticity of the causative agent of melioidosis, Burkholderia pseudomallei. *Proc. Natl. Acad. Sci. U.S.A.* **101,** 14240-5 (2004).

192. Stone, JK *et al.* Complete Genome Sequence of the Encephalomyelitic Burkholderia pseudomallei Strain MSHR305. *Genome Announc.* **1,** (2013).

193. Song, JY *et al.* Draft genome sequence of the antifungal-producing plant-benefiting bacterium Burkholderia pyrrocinia CH-67. *J. Bacteriol.* **194,** 6649-50 (2012).

194. Khan, A. Asif, H. Studholme, DJ. Khan, IA. Azim, MK. Genome characterization of a novel Burkholderia cepacia complex genomovar isolated from dieback affected mango orchards. *World J. Microbiol. Biotechnol.* **29,** 2033-44 (2013).

195. Kim, HS *et al.* Bacterial genome adaptation to niches: divergence of the potential virulence genes in three Burkholderia species of different survival strategies. *BMC Genomics.* **6,** 174 (2005).

196. Sim, BM *et al.* Genomic acquisition of a capsular polysaccharide virulence cluster by non-pathogenic Burkholderia isolates. *Genome Biol.* **11,** R89 (2010).

197. Zhuo, Y *et al.* Revised genome sequence of Burkholderia thailandensis MSMB43 with improved annotation. *J. Bacteriol.* **194,** 4749-50 (2012).

198. Cornelius, AJ *et al.* Complete Genome Sequence of *Campylobacter concisus* ATCC 33237^{T} and Draft Genome Sequences for an Additional Eight Well-Characterized *C. concisus* Strains. *Genome Announc.* **5,** (2017).

199. Miller, WG. Yee, E. Chapman, MH. Complete Genome Sequences of Campylobacter hyointestinalis subsp. hyointestinalis Strain LMG 9260 and C. hyointestinalis subsp. lawsonii Strain LMG 15993. *Genome Announc.* **4,** (2016).

200. Miller, WG. Yee, E. Huynh, S. Chapman, MH. Parker, CT. Complete Genome Sequence of Campylobacter iguaniorum Strain RM11343, Isolated from an Alpaca. *Genome Announc.* **4,** (2016).

201. Marasini, D. Fakhr, MK. Whole-Genome Sequencing of a Campylobacter jejuni Strain Isolated from Retail Chicken Meat Reveals the Presence of a Megaplasmid with Mu-Like Prophage and Multidrug Resistance Genes. *Genome Announc.* **4,** (2016).

202. Miller, WG. Yee, E. On, SL. Andersen, LP. Bono, JL. Complete Genome Sequence of the Campylobacter ureolyticus Clinical Isolate RIGS 9880. *Genome Announc.* **3,** (2015).

203. Vöing, K. Harrison, A. Soby, SD. Draft Genome Sequence of Chromobacterium vaccinii, a Potential Biocontrol Agent against Mosquito (Aedes aegypti) Larvae. *Genome Announc.* **3,** (2015).

204. Wang, X. Hinshaw, KC. Macdonald, SJ. Chandler, JR. Draft Genome Sequence of Chromobacterium violaceum Strain CV017. *Genome Announc.* **4,** (2016).

205. Chan, GF. Gan, HM. Rashid, NA. Genome sequence of Citrobacter sp. strain A1, a dye-degrading bacterium. *J. Bacteriol.* **194,** 5485-6 (2012).

206. Xin, B *et al.* Genome Sequence of Clostridium butyricum Strain DSM 10702, a Promising Producer of Biofuels and Biochemicals. *Genome Announc.* **1,** (2013).

207. Mela, F *et al.* Dual transcriptional profiling of a bacterial/fungal confrontation: Collimonas fungivorans versus Aspergillus niger. *ISME J.* **5,** 1494-504 (2011).

208. Methé, BA *et al.* The psychrophilic lifestyle as revealed by the genome sequence of Colwellia psychrerythraea 34H through genomic and proteomic analyses. *Proc. Natl. Acad. Sci. U.S.A.* **102,** 10913-8 (2005).

209. Ma, YF *et al.* The complete genome of Comamonas testosteroni reveals its genetic adaptations to changing environments. *Appl. Environ. Microbiol.* **75,** 6812-9 (2009).

210. Gibbons, HS *et al.* Comparative genomics of 2009 seasonal plague (Yersinia pestis) in New Mexico. *PLoS ONE.* **7,** e31604 (2012).

211. Baltrus, DA *et al.* Dynamic evolution of pathogenicity revealed by sequencing and comparative genomics of 19 Pseudomonas syringae isolates. *PLoS Pathog.* **7,** e1002132 (2011).

212. Toh, H. Oshima, K. Suzuki, T. Hattori, M. Morita, H. Complete Genome Sequence of the Equol-Producing Bacterium Adlercreutzia equolifaciens DSM 19450T. *Genome Announc.* **1,** (2013).

213. Boyle, B *et al.* Complete genome sequences of three Pseudomonas aeruginosa isolates with phenotypes of polymyxin B adaptation and inducible resistance. *J. Bacteriol.* **194,** 529-30 (2012).

214. Cserháti, M *et al.* De novo genome project of Cupriavidus basilensis OR16. *J. Bacteriol.* **194,** 2109-10 (2012).

215. Li, LG. Cai, L. Zhang, T. Genome of Cupriavidus sp. HMR-1, a Heavy Metal-Resistant Bacterium. *Genome Announc.* **1,** (2013).

216. Shafie, NA. Lau, NS. Ramachandran, H. Amirul, AA. Complete Genome Sequences of Three Cupriavidus Strains Isolated from Various Malaysian Environments. *Genome Announc.* **5,** (2017).

217. Seshadri, R *et al.* Genome sequence of the PCE-dechlorinating bacterium Dehalococcoides ethenogenes. *Science.* **307,** 105-8 (2005).

218. Saffarian, A *et al.* Complete Genome Sequence of Delftia tsuruhatensis CM13 Isolated from Murine Proximal Colonic Tissue. *Genome Announc.* **4,** (2016).

219. Abicht, HK. Mancini, S. Karnachuk, OV. Solioz, M. Genome sequence of Desulfosporosinus sp. OT, an acidophilic sulfate-reducing bacterium from copper mining waste in Norilsk, Northern Siberia. *J. Bacteriol.* **193,** 6104-5 (2011).

220. Poehlein, A. Daniel, R. Simeonova, DD. Draft Genome Sequence of Desulfotignum phosphitoxidans DSM 13687 Strain FiPS-3. *Genome Announc.* **1,** (2013).

221. Utturkar, SM *et al.* Draft Genome Sequence for Ralstonia sp. Strain OR214, a Bacterium with Potential for Bioremediation. *Genome Announc.* **1,** (2013).

222. Soares-Castro, P. Marques, D. Demyanchuk, S. Faustino, A. Santos, PM. Draft genome sequences of two Pseudomonas aeruginosa clinical isolates with different antibiotic susceptibilities. *J. Bacteriol.* **193,** 5573 (2011).

223. Hwangbo, K *et al.* Complete Genome Sequence of Dyella thiooxydans ATSB10, a Thiosulfate-Oxidizing Bacterium Isolated from Sunflower Fields in South Korea. *Genome Announc.* **4,** (2016).

224. Mathee, K *et al.* Dynamics of Pseudomonas aeruginosa genome evolution. *Proc. Natl. Acad. Sci. U.S.A.* **105,** 3100-5 (2008).

225. Cordero, OX *et al.* Ecological populations of bacteria act as socially cohesive units of antibiotic production and resistance. *Science.* **337,** 1228-31 (2012).

226. Chan, GF. Gan, HM. Rashid, NA. Genome sequence of Enterococcus sp. strain C1, an azo dye decolorizer. *J. Bacteriol.* **194,** 5716-7 (2012).

227. Hazen, TH *et al.* Draft genome sequences of the diarrheagenic Escherichia coli collection. *J. Bacteriol.* **194,** 3026-7 (2012).

228. Krause, DO. Little, AC. Dowd, SE. Bernstein, CN. Complete genome sequence of adherent invasive Escherichia coli UM146 isolated from Ileal Crohn's disease biopsy tissue. *J. Bacteriol.* **193,** 583 (2011).

229. Stephens, CM. Skerker, JM. Sekhon, MS. Arkin, AP. Riley, LW. Complete Genome Sequences of Four Escherichia coli ST95 Isolates from Bloodstream Infections. *Genome Announc.* **3,** (2015).

230. Zurfluh, K. Tasara, T. Stephan, R. Full-Genome Sequence of Escherichia coli K-15KW01, a Uropathogenic E. coli B2 Sequence Type 127 Isolate Harboring a Chromosomally Carried blaCTX-M-15 Gene. *Genome Announc.* **4,** (2016).

231. Rasko, DA *et al.* The pangenome structure of Escherichia coli: comparative genomic analysis of E. coli commensal and pathogenic isolates. *J. Bacteriol.* **190,** 6881-93 (2008).

232. Chen, SL *et al.* Identification of genes subject to positive selection in uropathogenic strains of Escherichia coli: a comparative genomics approach. *Proc. Natl. Acad. Sci. U.S.A.* **103,** 5977-82 (2006).

233. Luo, C *et al.* Genome sequencing of environmental Escherichia coli expands understanding of the ecology and speciation of the model bacterial species. *Proc. Natl. Acad. Sci. U.S.A.* **108,** 7200-5 (2011).

234. Jang, Y. Oh, HM. Kim, H. Kang, I. Cho, JC. Genome sequence of strain IMCC1989, a novel member of the marine gammaproteobacteria. *J. Bacteriol.* **193,** 3672-3 (2011).

235. Qi, M. Wang, D. Bradley, CA. Zhao, Y. Genome sequence analyses of Pseudomonas savastanoi pv. glycinea and subtractive hybridization-based comparative genomics with nine pseudomonads. *PLoS ONE.* **6,** e16451 (2011).

236. Woo, JK *et al.* Draft genome sequence of the chronic, nonclonal cystic fibrosis isolate Pseudomonas aeruginosa strain 18A. *Genome Announc.* **1,** e0000113 (2013).

237. Shapiro, BJ *et al.* Population genomics of early events in the ecological differentiation of bacteria. *Science.* **336,** 48-51 (2012).

238. Liu, B. Frostegård, A. Shapleigh, JP. Draft genome sequences of five strains in the genus thauera. *Genome Announc.* **1,** (2013).

239. Garzetti, D. Heesemann, J. Rakin, A. Genome Sequences of Four Yersinia enterocolitica Bioserotype 4/O:3 Isolates from Mammals. *Genome Announc.* **1,** (2013).

240. Eppinger, M *et al.* Draft genome sequences of Yersinia pestis isolates from natural foci of endemic plague in China. *J. Bacteriol.* **191,** 7628-9 (2009).

241. Semova, I *et al.* Microbiota regulate intestinal absorption and metabolism of fatty acids in the zebrafish. *Cell Host Microbe.* **12,** 277-88 (2012).

242. Uchimura, Y *et al.* Complete Genome Sequences of 12 Species of Stable Defined Moderately Diverse Mouse Microbiota 2. *Genome Announc.* **4,** (2016).

243. Richard, D *et al.* Complete Genome Sequences of Six Copper-Resistant *Xanthomonas citri* pv. citri Strains Causing Asiatic Citrus Canker, Obtained Using Long-Read Technology. *Genome Announc.* **5,** (2017).

244. Richard, D *et al.* Complete Genome Sequences of Six Copper-Resistant *Xanthomonas* Strains Causing Bacterial Spot of Solaneous Plants, Belonging to *X. gardneri*, *X.*

euvesicatoria, and *X. vesicatoria*, Using Long-Read Technology. *Genome Announc.* **5,** (2017).

245. Richard, D. Boyer, C. Lefeuvre, P. Pruvost, O. Complete Genome Sequence of a Copper-Resistant Bacterium from the Citrus Phyllosphere, Stenotrophomonas sp. Strain LM091, Obtained Using Long-Read Technology. *Genome Announc.* **4,** (2016).

246. Pillay, A *et al.* Complete Genome Sequences of 11 Haemophilus ducreyi Isolates from Children with Cutaneous Lesions in Vanuatu and Ghana. *Genome Announc.* **4,** (2016).

247. Coil, DA. Jospin, G. Eisen, JA. Adams, JY. Additional Draft Genome Sequences of Escherichia coli Strains Isolated from Septic Patients. *Genome Announc.* **4,** (2016).

248. Appolinario, LR *et al.* Description of Endozoicomonas arenosclerae sp. nov. using a genomic taxonomy approach. *Antonie Van Leeuwenhoek.* **109,** 431-8 (2016).

249. Spilker, T. LiPuma, JJ. Draft Genome Sequences of 63 Pseudomonas aeruginosa Isolates Recovered from Cystic Fibrosis Sputum. *Genome Announc.* **4,** (2016).

250. Marasini, D. Abo-Shama, UH. Fakhr, MK. Complete Genome Sequences of Salmonella enterica Serovars Anatum and Anatum var. 15+, Isolated from Retail Ground Turkey. *Genome Announc.* **4,** (2016).

251. Hau, SJ *et al.* Draft Genome Sequences of Nine Streptococcus suis Strains Isolated in the United States. *Genome Announc.* **3,** (2015).

252. Remenant, B *et al.* Genomes of three tomato pathogens within the Ralstonia solanacearum species complex reveal significant evolutionary divergence. *BMC Genomics.* **11,** 379 (2010).

253. Snitkin, ES *et al.* Genomic insights into the fate of colistin resistance and Acinetobacter baumannii during patient treatment. *Genome Res.* **23,** 1155-62 (2013).

254. Snitkin, ES *et al.* Genome-wide recombination drives diversification of epidemic strains of Acinetobacter baumannii. *Proc. Natl. Acad. Sci. U.S.A.* **108,** 13758-63 (2011).

255. Arivett, BA. Ream, DC. Fiester, SE. Kidane, D. Actis, LA. Draft Genome Sequences of Pseudomonas aeruginosa Isolates from Wounded Military Personnel. *Genome Announc.* **4,** (2016).

256. Arivett, BA. Ream, DC. Fiester, SE. Kidane, D. Actis, LA. Draft Genome Sequences of Acinetobacter baumannii Isolates from Wounded Military Personnel. *Genome Announc.* **4,** (2016).

257. Jordan, IK *et al.* Genome sequences for five strains of the emerging pathogen Haemophilus haemolyticus. *J. Bacteriol.* **193,** 5879-80 (2011).

258. Hogg, JS *et al.* Characterization and modeling of the Haemophilus influenzae core and supragenomes based on the complete genomic sequences of Rd and 12 clinical nontypeable strains. *Genome Biol.* **8,** R103 (2007).

259. Cardinali-Rezende, J *et al.* Draft Genome Sequence of Halomonas sp. HG01, a Polyhydroxyalkanoate-Accumulating Strain Isolated from Peru. *Genome Announc.* **4,** (2016).

260. O'Dell, KB *et al.* Genome Sequence of Halomonas sp. Strain KO116, an Ionic Liquid-Tolerant Marine Bacterium Isolated from a Lignin-Enriched Seawater Microcosm. *Genome Announc.* **3,** (2015).

261. Ye, W *et al.* Genome sequence of the pathogenic Herbaspirillum seropedicae strain Os34, isolated from rice roots. *J. Bacteriol.* **194,** 6993-4 (2012).

262. Zhu, B *et al.* Genome sequence of the pathogenic Herbaspirillum seropedicae strain Os45, isolated from rice roots. *J. Bacteriol.* **194,** 6995-6 (2012).

263. Muller, D *et al.* A tale of two oxidation states: bacterial colonization of arsenic-rich environments. *PLoS Genet.* **3,** e53 (2007).

264. Tao, F *et al.* Genome sequence of Xanthomonas campestris JX, an industrially productive strain for Xanthan gum. *J. Bacteriol.* **194,** 4755-6 (2012).

265. Bart, R *et al.* High-throughput genomic sequencing of cassava bacterial blight strains identifies conserved effectors to target for durable resistance. *Proc. Natl. Acad. Sci. U.S.A.* **109,** E1972-9 (2012).

266. Dewhirst, FE. Chen, CK. Paster, BJ. Zambon, JJ. Phylogeny of species in the family Neisseriaceae isolated from human dental plaque and description of Kingella oralis sp. nov [corrected]. *Int. J. Syst. Bacteriol.* **43,** 490-9 (1993).

267. Peleg, AY *et al.* The success of acinetobacter species; genetic, metabolic and virulence attributes. *PLoS ONE.* **7,** e46984 (2012).

268. Robinson, LS *et al.* Genome Sequences of 15 Gardnerella vaginalis Strains Isolated from the Vaginas of Women with and without Bacterial Vaginosis. *Genome Announc.* **4,** (2016).

269. Marcy, Y *et al.* Dissecting biological "dark matter" with single-cell genetic analysis of rare and uncultivated TM7 microbes from the human mouth. *Proc. Natl. Acad. Sci. U.S.A.* **104,** 11889-94 (2007).

270. Kittichotirat, W *et al.* Genome sequence of Methyloversatilis universalis FAM5T, a methylotrophic representative of the order Rhodocyclales. *J. Bacteriol.* **193,** 4541-2 (2011).

271. Audic, S *et al.* Genome analysis of Minibacterium massiliensis highlights the convergent evolution of water-living bacteria. *PLoS Genet.* **3,** e138 (2007).

272. Kaplan, JB *et al.* Genome sequence of Kingella kingae septic arthritis isolate PYKK081. *J. Bacteriol.* **194,** 3017 (2012).

273. Alexiev, A. Coil, DA. Jospin, G. Eisen, JA. Adams, JY. Draft Genome Sequence of Klebsiella pneumoniae UCD-JA29 Isolated from a Patient with Sepsis. *Genome Announc.* **4,** (2016).

274. Liu, PP. Liu, Y. Wang, LH. Wei, DD. Wan, LG. Draft Genome Sequence of an NDM-5-Producing Klebsiella pneumoniae Sequence Type 14 Strain of Serotype K2. *Genome Announc.* **4,** (2016).

275. Naka, H *et al.* Complete genome sequence of the marine fish pathogen Vibrio anguillarum harboring the pJM1 virulence plasmid and genomic comparison with other virulent strains of V. anguillarum and V. ordalii. *Infect. Immun.* **79,** 2889-900 (2011).

276. Yi, JL *et al.* Draft Genome Sequence of the Bacterium Lysobacter capsici X2-3, with a Broad Spectrum of Antimicrobial Activity against Multiple Plant-Pathogenic Microbes. *Genome Announc.* **3,** (2015).

277. Hauglund, MJ. Tatum, FM. Bayles, DO. Maheswaran, SK. Briggs, RE. Genome Sequences of Mannheimia haemolytica Serotype A2 Isolates D171 and D35, Recovered from Bovine Pneumonia. *Genome Announc.* **3,** (2015).

278. Eidam, C *et al.* Complete Genome Sequence of Mannheimia haemolytica Strain 42548 from a Case of Bovine Respiratory Disease. *Genome Announc.* **1,** (2013).

279. Harhay, GP *et al.* Complete Closed Genome Sequences of Mannheimia haemolytica Serotypes A1 and A6, Isolated from Cattle. *Genome Announc.* **1,** (2013).

280. Duhaime, MB. Wichels, A. Sullivan, MB. Six Pseudoalteromonas Strains Isolated from Surface Waters of Kabeltonne, Offshore Helgoland, North Sea. *Genome Announc.* **4,** (2016).

281. Gärdes, A *et al.* Complete genome sequence of Marinobacter adhaerens type strain (HP15), a diatom-interacting marine microorganism. *Stand Genomic Sci.* **3,** 97-107 (2010).

282. Grimaud, R *et al.* Genome sequence of the marine bacterium Marinobacter hydrocarbonoclasticus SP17, which forms biofilms on hydrophobic organic compounds. *J. Bacteriol.* **194,** 3539-40 (2012).

283. Regar, RK *et al.* Draft Genome Sequence of Acinetobacter baumannii IITR88, a Bacterium Degrading Indoles and Other Aromatic Compounds. *Genome Announc.* **4,** (2016).

284. Davie, JJ *et al.* Comparative analysis and supragenome modeling of twelve Moraxella catarrhalis clinical isolates. *BMC Genomics.* **12,** 70 (2011).

285. Campbell, BJ *et al.* Adaptations to submarine hydrothermal environments exemplified by the genome of Nautilia profundicola. *PLoS Genet.* **5,** e1000362 (2009).

286. Ribeiro, FJ *et al.* Finished bacterial genomes from shotgun sequence data. *Genome Res.* **22,** 2270-7 (2012).

287. Chung, GT *et al.* Complete genome sequence of Neisseria gonorrhoeae NCCP11945. *J. Bacteriol.* **190,** 6035-6 (2008).

288. Chen, CC *et al.* Draft genome sequence of a dominant, multidrug-resistant Neisseria gonorrhoeae strain, TCDC-NG08107, from a sexual group at high risk of acquiring human immunodeficiency virus infection and syphilis. *J. Bacteriol.* **193,** 1788-9 (2011).

289. Bennett, JS *et al.* Independent evolution of the core and accessory gene sets in the genus Neisseria: insights gained from the genome of Neisseria lactamica isolate 020-06. *BMC Genomics.* **11,** 652 (2010).

290. Peng, J *et al.* Characterization of ST-4821 complex, a unique Neisseria meningitidis clone. *Genomics.* **91,** 78-87 (2008).

291. Joseph, B *et al.* Comparative genome biology of a serogroup B carriage and disease strain supports a polygenic nature of meningococcal virulence. *J. Bacteriol.* **192,** 5363-77 (2010).

292. Bentley, SD *et al.* Meningococcal genetic variation mechanisms viewed through comparative analysis of serogroup C strain FAM18. *PLoS Genet.* **3,** e23 (2007).

293. Budroni, S *et al.* Neisseria meningitidis is structured in clades associated with restriction modification systems that modulate homologous recombination. *Proc. Natl. Acad. Sci. U.S.A.* **108,** 4494-9 (2011).

294. Lavezzo, E *et al.* Draft genome sequences of two Neisseria meningitidis serogroup C clinical isolates. *J. Bacteriol.* **192,** 5270-1 (2010).

295. Vogel, U *et al.* Ion torrent personal genome machine sequencing for genomic typing of Neisseria meningitidis for rapid determination of multiple layers of typing information. *J. Clin. Microbiol.* **50,** 1889-94 (2012).

296. Piet, JR *et al.* Genome sequence of Neisseria meningitidis serogroup B strain H44/76. *J. Bacteriol.* **193,** 2371-2 (2011).

297. Tettelin, H *et al.* Complete genome sequence of Neisseria meningitidis serogroup B strain MC58. *Science.* **287,** 1809-15 (2000).

298. Hao, W *et al.* Extensive genomic variation within clonal complexes of Neisseria meningitidis. *Genome Biol Evol.* **3,** 1406-18 (2011).

299. Schoen, C *et al.* Whole-genome sequence of the transformable Neisseria meningitidis serogroup A strain WUE2594. *J. Bacteriol.* **193,** 2064-5 (2011).

300. Parkhill, J *et al.* Complete DNA sequence of a serogroup A strain of Neisseria meningitidis Z2491. *Nature.* **404,** 502-6 (2000).

301. Diéguez, AL. Romalde, JL. Draft Genome Sequences of Neptuniibacter sp. Strains LFT 1.8 and ATR 1.1. *Genome Announc.* **5,** (2017).

302. Yeganeh, LP *et al.* Complete genome sequence of Oceanimonas sp. GK1, a halotolerant bacterium from Gavkhouni Wetland in Iran. *J. Bacteriol.* **194,** 2123-4 (2012).

303. Chauhan, A. Green, S. Pathak, A. Thomas, J. Venkatramanan, R. Whole-genome sequences of five oyster-associated bacteria show potential for crude oil hydrocarbon degradation. *Genome Announc.* **1,** (2013).

304. Qin, X. Evans, JD. Aronstein, KA. Murray, KD. Weinstock, GM. Genome sequences of the honey bee pathogens Paenibacillus larvae and Ascosphaera apis. *Insect Mol. Biol.* **15,** 715-8 (2006).

305. Pushiri, H. Pearce, SL. Oakeshott, JG. Russell, RJ. Pandey, G. Draft Genome Sequence of Pandoraea sp. Strain SD6-2, Isolated from Lindane-Contaminated Australian Soil. *Genome Announc.* **1,** (2013).

306. Koskinen, JP *et al.* Genome sequence of Pectobacterium sp. strain SCC3193. *J. Bacteriol.* **194,** 6004 (2012).

307. Nykyri, J *et al.* Revised phylogeny and novel horizontally acquired virulence determinants of the model soft rot phytopathogen Pectobacterium wasabiae SCC3193. *PLoS Pathog.* **8,** e1003013 (2012).

308. Ghazal, S *et al.* Draft Genome Sequence of Photorhabdus luminescens subsp. laumondii HP88, an Entomopathogenic Bacterium Isolated from Nematodes. *Genome Announc.* **4,** (2016).

309. Hira, D *et al.* Anammox organism KSU-1 expresses a NirK-type copper-containing nitrite reductase instead of a NirS-type with cytochrome cd1. *FEBS Lett.* **586,** 1658-63 (2012).

310. Brown, SD *et al.* Twenty-one genome sequences from Pseudomonas species and 19 genome sequences from diverse bacteria isolated from the rhizosphere and endosphere of Populus deltoides. *J. Bacteriol.* **194,** 5991-3 (2012).

311. Di Pilato, V *et al.* Complete Genome Sequence of the First KPC-Type Carbapenemase-Positive Proteus mirabilis Strain from a Bloodstream Infection. *Genome Announc.* **4,** (2016).

312. Galac, MR. Lazzaro, BP. Comparative genomics of bacteria in the genus Providencia isolated from wild Drosophila melanogaster. *BMC Genomics.* **13,** 612 (2012).

313. Clifford, RJ *et al.* Complete genome sequence of Providencia stuartii clinical isolate MRSN 2154. *J. Bacteriol.* **194,** 3736-7 (2012).

314. de la Haba, RR. Sánchez-Porro, C. León, MJ. Papke, RT. Ventosa, A. Draft Genome Sequence of the Moderately Halophilic Bacterium Pseudoalteromonas ruthenica Strain CP76. *Genome Announc.* **1,** (2013).

315. Ishii, S *et al.* Complete genome sequence of the denitrifying and N(2)O-reducing bacterium Pseudogulbenkiania sp. strain NH8B. *J. Bacteriol.* **193,** 6395-6 (2011).

316. Karna, SL *et al.* Genome Sequence of a Virulent Pseudomonas aeruginosa Strain, 12-4-4(59), Isolated from the Blood Culture of a Burn Patient. *Genome Announc.* **4,** (2016).

317. Dotson, GA *et al.* Draft Genome Sequence of a Klebsiella pneumoniae Carbapenemase-Positive Sequence Type 111 Pseudomonas aeruginosa Strain. *Genome Announc.* **4,** (2016).

318. Naughton, S *et al.* Pseudomonas aeruginosa AES-1 exhibits increased virulence gene expression during chronic infection of cystic fibrosis lung. *PLoS ONE.* **6,** e24526 (2011).

319. Wu, DQ *et al.* Genome sequence of Pseudomonas aeruginosa strain AH16, isolated from a patient with chronic pneumonia in China. *J. Bacteriol.* **194,** 5976-7 (2012).

320. Zhong, C. Nelson, M. Cao, G. Sadowsky, MJ. Yan, T. Complete Genome Sequence of the Triclosan- and Multidrug-Resistant Pseudomonas aeruginosa Strain B10W Isolated from Municipal Wastewater. *Genome Announc.* **5,** (2017).

321. Sanjar, F *et al.* Whole-Genome Sequence of Multidrug-Resistant Pseudomonas aeruginosa Strain BAMCPA07-48, Isolated from a Combat Injury Wound. *Genome Announc.* **4,** (2016).

322. Yin, Y. Withers, TR. Johnson, SL. Yu, HD. Draft Genome Sequence of a Mucoid Isolate of Pseudomonas aeruginosa Strain C7447m from a Patient with Cystic Fibrosis. *Genome Announc.* **1,** (2013).

323. Winstanley, C *et al.* Newly introduced genomic prophage islands are critical determinants of in vivo competitiveness in the Liverpool Epidemic Strain of Pseudomonas aeruginosa. *Genome Res.* **19,** 12-23 (2009).

324. Wu, DQ *et al.* Genomic analysis and temperature-dependent transcriptome profiles of the rhizosphere originating strain Pseudomonas aeruginosa M18. *BMC Genomics.* **12,** 438 (2011).

325. Olivas, AD *et al.* Intestinal tissues induce an SNP mutation in Pseudomonas aeruginosa that enhances its virulence: possible role in anastomotic leak. *PLoS ONE.* **7,** e44326 (2012).

326. Weigand, MR. Sundin, GW. General and inducible hypermutation facilitate parallel adaptation in Pseudomonas aeruginosa despite divergent mutation spectra. *Proc. Natl. Acad. Sci. U.S.A.* **109,** 13680-5 (2012).

327. Chan, KG. Wong, CS. Yin, WF. Chan, XY. Draft Genome Sequence of Quorum-Sensing and Quorum-Quenching Pseudomonas aeruginosa Strain MW3a. *Genome Announc.* **2,** (2014).

328. Tada, T. Kitao, T. Miyoshi-Akiyama, T. Kirikae, T. Genome sequence of multidrug-resistant Pseudomonas aeruginosa NCGM1179. *J. Bacteriol.* **193,** 6397 (2011).

329. Viedma, E. Juan, C. Otero, JR. Oliver, A. Chaves, F. Draft Genome Sequence of VIM-2-Producing Multidrug-Resistant Pseudomonas aeruginosa ST175, an Epidemic High-Risk Clone. *Genome Announc.* **1,** e0011213 (2013).

330. Segata, N. Ballarini, A. Jousson, O. Genome Sequence of Pseudomonas aeruginosa PA45, a Highly Virulent Strain Isolated from a Patient with Bloodstream Infection. *Genome Announc.* **1,** (2013).

331. Roy, PH *et al.* Complete genome sequence of the multiresistant taxonomic outlier Pseudomonas aeruginosa PA7. *PLoS ONE.* **5,** e8842 (2010).

332. Ozer, EA. Allen, JP. Hauser, AR. Draft genome sequence of the Pseudomonas aeruginosa bloodstream isolate PABL056. *J. Bacteriol.* **194,** 5999 (2012).

333. Stover, CK *et al.* Complete genome sequence of Pseudomonas aeruginosa PAO1, an opportunistic pathogen. *Nature.* **406,** 959-64 (2000).

334. Withers, TR. Johnson, SL. Yu, HD. Draft genome sequence for Pseudomonas aeruginosa strain PAO579, a mucoid derivative of PAO381. *J. Bacteriol.* **194,** 6617 (2012).

335. Qiu, D. Eisinger, VM. Head, NE. Pier, GB. Yu, HD. ClpXP proteases positively regulate alginate overexpression and mucoid conversion in Pseudomonas aeruginosa. *Microbiology (Reading, Engl.).* **154,** 2119-30 (2008).

336. Pragasam, AK *et al.* Draft Genome Sequence of Extremely Drug-Resistant Pseudomonas aeruginosa (ST357) Strain CMC_VB_PA_B22862 Isolated from a Community-Acquired Bloodstream Infection. *Genome Announc.* **4,** (2016).

337. Silo-Suh, LA. Suh, SJ. Ohman, DE. Wozniak, DJ. Pridgeon, JW. Complete Genome Sequence of Pseudomonas aeruginosa Mucoid Strain FRD1, Isolated from a Cystic Fibrosis Patient. *Genome Announc.* **3,** (2015).

338. Wang, D. Hildebrand, F. Ye, L. Wei, Q. Ma, LZ. Genome Sequence of Mucoid Pseudomonas aeruginosa Strain FRD1. *Genome Announc.* **3,** (2015).

339. Manivannan, B *et al.* Draft Genome Sequence of a Clinically Isolated Extensively Drug-Resistant Pseudomonas aeruginosa Strain. *Genome Announc.* **4,** (2016).

340. Lee, DG *et al.* Genomic analysis reveals that Pseudomonas aeruginosa virulence is combinatorial. *Genome Biol.* **7,** R90 (2006).

341. Santopolo, L *et al.* Draft Genome Sequence of Chromate-Resistant and Biofilm-Producing Strain Pseudomonas alcaliphila 34. *Genome Announc.* **1,** (2013).

342. Ainala, SK. Somasundar, A. Park, S. Complete Genome Sequence of Pseudomonas denitrificans ATCC 13867. *Genome Announc.* **1,** (2013).

343. Wong, CF *et al.* Genome sequence of Pseudomonas mendocina DLHK, isolated from a biotrickling reactor. *J. Bacteriol.* **194,** 6326 (2012).

344. Guo, W *et al.* Complete genome of Pseudomonas mendocina NK-01, which synthesizes medium-chain-length polyhydroxyalkanoates and alginate oligosaccharides. *J. Bacteriol.* **193,** 3413-4 (2011).

345. El-Said Mohamed, M *et al.* Genome Sequence of Pseudomonas azelaica Strain Aramco J. *Genome Announc.* **3,** (2015).

346. Triscari-Barberi, T *et al.* Genome sequence of the polychlorinated-biphenyl degrader Pseudomonas pseudoalcaligenes KF707. *J. Bacteriol.* **194,** 4426-7 (2012).

347. Liu, B *et al.* Complete Genome Sequence of a Marine Bacterium, Pseudomonas pseudoalcaligenes Strain S1, with High Mercury Resistance and Bioaccumulation Capacity. *Genome Announc.* **4,** (2016).

348. Park, JY *et al.* Draft genome sequence of the biocontrol bacterium Pseudomonas putida B001, an oligotrophic bacterium that induces systemic resistance to plant diseases. *J. Bacteriol.* **193,** 6795-6 (2011).

349. Shintani, M *et al.* Complete Genome Sequence of the Carbazole Degrader Pseudomonas resinovorans Strain CA10 (NBRC 106553). *Genome Announc.* **1,** (2013).

350. Mishra, SR *et al.* Draft Genome Sequence of Pseudomonas sp. Strain BMS12, a Plant Growth-Promoting and Protease-Producing Bacterium, Isolated from the Rhizosphere Sediment of Phragmites karka of Chilika Lake, India. *Genome Announc.* **4,** (2016).

351. Qu, Y *et al.* Genome Sequence of an Indigoid-Producing Strain, Pseudomonas sp. PI1. *Genome Announc.* **3,** (2015).

352. Chong, TM *et al.* Heavy-metal resistance of a France vineyard soil bacterium, Pseudomonas mendocina strain S5.2, revealed by whole-genome sequencing. *J. Bacteriol.* **194,** 6366 (2012).

353. Brunet-Galmés, I *et al.* Complete genome sequence of the naphthalene-degrading bacterium Pseudomonas stutzeri AN10 (CCUG 29243). *J. Bacteriol.* **194,** 6642-3 (2012).

354. Busquets, A *et al.* Genome sequence of Pseudomonas stutzeri strain JM300 (DSM 10701), a soil isolate and model organism for natural transformation. *J. Bacteriol.* **194,** 5477-8 (2012).

355. Li, A *et al.* Genome sequence of a highly efficient aerobic denitrifying bacterium, Pseudomonas stutzeri T13. *J. Bacteriol.* **194,** 5720 (2012).

356. Liu, X *et al.* Genome sequences of Pseudomonas luteola XLDN4-9 and Pseudomonas stutzeri XLDN-R, two efficient carbazole-degrading strains. *J. Bacteriol.* **194,** 5701-2 (2012).

357. Visnovsky, SB *et al.* Draft Genome Sequences of 18 Strains of Pseudomonas Isolated from Kiwifruit Plants in New Zealand and Overseas. *Genome Announc.* **4,** (2016).

358. Zheng, D *et al.* Genome Sequence of Pseudomonas citronellolis SJTE-3, an Estrogen- and Polycyclic Aromatic Hydrocarbon-Degrading Bacterium. *Genome Announc.* **4,** (2016).

359. Zou, C *et al.* Draft Genome Sequence of Ralstonia solanacearum Strain Rs-T02, Which Represents the Most Prevalent Phylotype in Guangxi, China. *Genome Announc.* **4,** (2016).

360. Gabriel, DW *et al.* Identification of open reading frames unique to a select agent: Ralstonia solanacearum race 3 biovar 2. *Mol. Plant Microbe Interact.* **19,** 69-79 (2006).

361. Guarischi-Sousa, R *et al.* Complete genome sequence of the potato pathogen Ralstonia solanacearum UY031. *Stand Genomic Sci.* **11,** 7 (2016).

362. Li, Z *et al.* Genome sequence of the tobacco bacterial wilt pathogen Ralstonia solanacearum. *J. Bacteriol.* **193,** 6088-9 (2011).

363. Kelly, WJ *et al.* The complete genome sequence of Eubacterium limosum SA11, a metabolically versatile rumen acetogen. *Stand Genomic Sci.* **11,** 26 (2016).

364. Bao, HX *et al.* Differential efficiency in exogenous DNA acquisition among closely related Salmonella strains: implications in bacterial speciation. *BMC Microbiol.* **14,** 157 (2014).

365. Allard, MW *et al.* On the evolutionary history, population genetics and diversity among isolates of Salmonella Enteritidis PFGE pattern JEGX01.0004. *PLoS ONE.* **8,** e55254 (2013).

366. Allard, MW *et al.* High resolution clustering of Salmonella enterica serovar Montevideo strains using a next-generation sequencing approach. *BMC Genomics.* **13,** 32 (2012).

367. Yap, KP *et al.* Genome sequence and comparative pathogenomics analysis of a Salmonella enterica Serovar Typhi strain associated with a typhoid carrier in Malaysia. *J. Bacteriol.* **194,** 5970-1 (2012).

368. Zhu, S *et al.* Non-contiguous finished genome sequence and description of Salmonella enterica subsp. houtenae str. RKS3027. *Stand Genomic Sci.* **8,** 198-205 (2013).

369. Baddam, R *et al.* Genetic fine structure of a Salmonella enterica serovar Typhi strain associated with the 2005 outbreak of typhoid fever in Kelantan, Malaysia. *J. Bacteriol.* **194,** 3565-6 (2012).

370. Wang, F *et al.* Environmental adaptation: genomic analysis of the piezotolerant and psychrotolerant deep-sea iron reducing bacterium Shewanella piezotolerans WP3. *PLoS ONE.* **3,** e1937 (2008).

371. Lo, WS. Chen, LL. Chung, WC. Gasparich, GE. Kuo, CH. Comparative genome analysis of Spiroplasma melliferum IPMB4A, a honeybee-associated bacterium. *BMC Genomics.* **14,** 22 (2013).

372. Zhang, L. Morrison, M. O Cuív, P. Evans, P. Rickard, CM. Genome Sequence of Stenotrophomonas maltophilia Strain AU12-09, Isolated from an Intravascular Catheter. *Genome Announc.* **1,** (2013).

373. Lira, F *et al.* Whole-genome sequence of Stenotrophomonas maltophilia D457, a clinical isolate and a model strain. *J. Bacteriol.* **194,** 3563-4 (2012).

374. Sassera, D *et al.* Draft Genome Sequence of Stenotrophomonas maltophilia Strain EPM1, Found in Association with a Culture of the Human Parasite Giardia duodenalis. *Genome Announc.* **1,** e0018213 (2013).

375. Crossman, LC *et al.* The complete genome, comparative and functional analysis of Stenotrophomonas maltophilia reveals an organism heavily shielded by drug resistance determinants. *Genome Biol.* **9,** R74 (2008).

376. Conchillo-Solé, O *et al.* Draft Genome Sequence of Stenotrophomonas maltophilia Strain UV74 Reveals Extensive Variability within Its Genomic Group. *Genome Announc.* **3,** (2015).

377. Chugani, S *et al.* Strain-dependent diversity in the Pseudomonas aeruginosa quorum-sensing regulon. *Proc. Natl. Acad. Sci. U.S.A.* **109,** E2823-31 (2012).

378. Cai, L. Shao, MF. Zhang, T. Non-contiguous finished genome sequence and description of Sulfurimonas hongkongensis sp. nov., a strictly anaerobic denitrifying, hydrogen- and sulfur-oxidizing chemolithoautotroph isolated from marine sediment. *Stand Genomic Sci.* **9,** 1302-10 (2014).

379. Jeon, W *et al.* Complete genome sequence of the sulfur-oxidizing chemolithoautotrophic *Sulfurovum lithotrophicum* 42BKT^{T}. *Stand Genomic Sci.* **12,** 54 (2017).

380. Dichosa, AE *et al.* Draft Genome Sequence of Thauera sp. Strain SWB20, Isolated from a Singapore Wastewater Treatment Facility Using Gel Microdroplets. *Genome Announc.* **3,** (2015).

381. Chen, D *et al.* Complete Genome Sequence of *Ralstonia solanacearum* FJAT-1458, a Potential Biocontrol Agent for Tomato Wilt. *Genome Announc.* **5,** (2017).

382. Kondo, H *et al.* Draft Genome Sequences of Six Strains of Vibrio parahaemolyticus Isolated from Early Mortality Syndrome/Acute Hepatopancreatic Necrosis Disease Shrimp in Thailand. *Genome Announc.* **2,** (2014).

383. Gómez-Consarnau, L *et al.* Proteorhodopsin phototrophy promotes survival of marine bacteria during starvation. *PLoS Biol.* **8,** e1000358 (2010).

384. Amaral, GR *et al.* Genome sequence of the bacterioplanktonic, mixotrophic Vibrio campbellii strain PEL22A, isolated in the Abrolhos Bank. *J. Bacteriol.* **194,** 2759-60 (2012).

385. Gan, HY *et al.* Genome Sequence of Vibrio campbellii Strain UMTGB204, a Marine Bacterium Isolated from a Green Barrel Tunicate. *Genome Announc.* **3,** (2015).

386. Taviani, E. Grim, CJ. Chun, J. Huq, A. Colwell, RR. Genomic analysis of a novel integrative conjugative element in Vibrio cholerae. *FEBS Lett.* **583,** 3630-6 (2009).

387. Grim, CJ *et al.* Genome sequence of hybrid Vibrio cholerae O1 MJ-1236, B-33, and CIRS101 and comparative genomics with V. cholerae. *J. Bacteriol.* **192,** 3524-33 (2010).

388. Garza, DR *et al.* Genome-wide study of the defective sucrose fermenter strain of Vibrio cholerae from the Latin American cholera epidemic. *PLoS ONE.* **7,** e37283 (2012).

389. Balakhonov, SV *et al.* Whole-Genome Sequencing of a Vibrio cholerae El Tor Strain Isolated in the Imported Cholera Focus in Siberia. *Genome Announc.* **3,** (2015).

390. Heidelberg, JF *et al.* DNA sequence of both chromosomes of the cholera pathogen Vibrio cholerae. *Nature.* **406,** 477-83 (2000).

391. Smirnova, NI *et al.* Whole-Genome Sequencing of *Vibrio cholerae* O1 El Tor Strains Isolated in Ukraine (2011) and Russia (2014). *Genome Announc.* **5,** (2017).

392. le Roux, WJ. Chan, WY. De Maayer, P. Venter, SN. Genome Sequence of Vibrio cholerae G4222, a South African Clinical Isolate. *Genome Announc.* **1,** e0004013 (2013).

393. Feng, L *et al.* A recalibrated molecular clock and independent origins for the cholera pandemic clones. *PLoS ONE.* **3,** e4053 (2008).

394. Kuleshov, KV *et al.* Draft Genome Sequences of Vibrio cholerae O1 ElTor Strains 2011EL-301 and P-18785, Isolated in Russia. *Genome Announc.* **1,** (2013).

395. Reimer, AR *et al.* Comparative genomics of Vibrio cholerae from Haiti, Asia, and Africa. *Emerging Infect. Dis.* **17,** 2113-21 (2011).

396. Kimes, NE *et al.* Temperature regulation of virulence factors in the pathogen Vibrio coralliilyticus. *ISME J.* **6,** 835-46 (2012).

397. Santos, Ede O *et al.* Genomic and proteomic analyses of the coral pathogen Vibrio coralliilyticus reveal a diverse virulence repertoire. *ISME J.* **5,** 1471-83 (2011).

398. Khatri, I. Mahajan, S. Dureja, C. Subramanian, S. Raychaudhuri, S. Evidence of a new metabolic capacity in an emerging diarrheal pathogen: lessons from the draft genomes of Vibrio fluvialis strains PG41 and I21563. *Gut Pathog.* **5,** 20 (2013).

399. Thompson, CC *et al.* Genomic taxonomy of Vibrios. *BMC Evol. Biol.* **9,** 258 (2009).

400. Espinoza-Valles, I *et al.* Draft genome sequence of the shrimp pathogen Vibrio harveyi CAIM 1792. *J. Bacteriol.* **194,** 2104 (2012).

401. Huang, Y *et al.* Draft genome sequence of the fish pathogen Vibrio harveyi strain ZJ0603. *J. Bacteriol.* **194,** 6644-5 (2012).

402. Hoffmann, M *et al.* Vibrio caribbeanicus sp. nov., isolated from the marine sponge Scleritoderma cyanea. *Int. J. Syst. Evol. Microbiol.* **62,** 1736-43 (2012).

403. Hasan, NA *et al.* Comparative genomics of clinical and environmental Vibrio mimicus. *Proc. Natl. Acad. Sci. U.S.A.* **107,** 21134-9 (2010).

404. Gonzalez-Escalona, N. Strain, EA. De Jesús, AJ. Jones, JL. Depaola, A. Genome sequence of the clinical O4:K12 serotype Vibrio parahaemolyticus strain 10329. *J. Bacteriol.* **193,** 3405-6 (2011).

405. Chen, Y *et al.* Comparative genomic analysis of Vibrio parahaemolyticus: serotype conversion and virulence. *BMC Genomics.* **12,** 294 (2011).

406. Makino, K *et al.* Genome sequence of Vibrio parahaemolyticus: a pathogenic mechanism distinct from that of V cholerae. *Lancet.* **361,** 743-9 (2003).

407. Jun, JW *et al.* Draft Genome Sequence of Vibrio parahaemolyticus SNUVpS-1 Isolated from Korean Seafood. *Genome Announc.* **1,** (2013).

408. Prabhakaran, DM. Chowdhury, G. Pazhani, GP. Ramamurthy, T. Thomas, S. Draft Genome Sequence of an Environmental trh+ Vibrio parahaemolyticus K23 Strain Isolated from Kerala, India. *Genome Announc.* **4,** (2016).

409. Liu, M. Chen, S. Draft Genome Sequence of Vibrio parahaemolyticus V110, Isolated from Shrimp in Hong Kong. *Genome Announc.* **1,** (2013).

410. Roy Chowdhury, P *et al.* Genome sequence of Vibrio rotiferianus strain DAT722. *J. Bacteriol.* **193,** 3381-2 (2011).

411. Lee, RD. Jospin, G. Lang, JM. Eisen, JA. Coil, DA. Draft Genome Sequences of Two Vibrio splendidus Strains, Isolated from Seagrass Sediment. *Genome Announc.* **4,** (2016).

412. Morrison, SS *et al.* Pyrosequencing-based comparative genome analysis of Vibrio vulnificus environmental isolates. *PLoS ONE.* **7,** e37553 (2012).

413. Park, JH *et al.* Complete genome sequence of Vibrio vulnificus MO6-24/O. *J. Bacteriol.* **193,** 2062-3 (2011).

414. Wu, CH. Chen, CY. Morales, C. Kiang, D. Draft Genome Sequence of an ortho-Nitrophenyl-β-d-Galactoside (ONPG)-Negative Strain of Vibrio cholerae, Isolated from Drakes Bay, California. *Genome Announc.* **4,** (2016).

415. Salvà-Serra, F *et al.* Genome Sequences of Two Naphthalene-Degrading Strains of *Pseudomonas balearica*, Isolated from Polluted Marine Sediment and from an Oil Refinery Site. *Genome Announc.* **5,** (2017).

416. Pieretti, I *et al.* The complete genome sequence of Xanthomonas albilineans provides new insights into the reductive genome evolution of the xylem-limited Xanthomonadaceae. *BMC Genomics.* **10,** 616 (2009).

417. Robène, I *et al.* High-Quality Draft Genome Sequences of Two Xanthomonas Pathotype Strains Infecting Aroid Plants. *Genome Announc.* **4,** (2016).

418. Qian, W *et al.* Comparative and functional genomic analyses of the pathogenicity of phytopathogen Xanthomonas campestris pv. campestris. *Genome Res.* **15,** 757-67 (2005).

419. da Silva, AC *et al.* Comparison of the genomes of two Xanthomonas pathogens with differing host specificities. *Nature.* **417,** 459-63 (2002).

420. Thieme, F *et al.* Insights into genome plasticity and pathogenicity of the plant pathogenic bacterium Xanthomonas campestris pv. vesicatoria revealed by the complete genome sequence. *J. Bacteriol.* **187,** 7254-66 (2005).

421. Bolot, S *et al.* Genome Sequence of Xanthomonas campestris pv. campestris Strain Xca5. *Genome Announc.* **1,** (2013).

422. Studholme, DJ *et al.* Genome-wide sequencing data reveals virulence factors implicated in banana Xanthomonas wilt. *FEMS Microbiol. Lett.* **310,** 182-92 (2010).

423. Bogdanove, AJ *et al.* Two new complete genome sequences offer insight into host and tissue specificity of plant pathogenic Xanthomonas spp. *J. Bacteriol.* **193,** 5450-64 (2011).

424. Bolot, S *et al.* Draft Genome Sequence of the Xanthomonas cassavae Type Strain CFBP 4642. *Genome Announc.* **1,** (2013).

425. Jalan, N *et al.* Complete Genome Sequence of Xanthomonas citri subsp. citri Strain Aw12879, a Restricted-Host-Range Citrus Canker-Causing Bacterium. *Genome Announc.* **1,** (2013).

426. Cunnac, S *et al.* High-Quality Draft Genome Sequences of Two Xanthomonas citri pv. malvacearum Strains. *Genome Announc.* **1,** (2013).

427. Henry, PM. Leveau, JH. Finished Genome Sequences of Xanthomonas fragariae, the Cause of Bacterial Angular Leaf Spot of Strawberry. *Genome Announc.* **4,** (2016).

428. Moreira, LM *et al.* Novel insights into the genomic basis of citrus canker based on the genome sequences of two strains of Xanthomonas fuscans subsp. aurantifolii. *BMC Genomics.* **11,** 238 (2010).

429. Darrasse, A *et al.* Genome sequence of Xanthomonas fuscans subsp. fuscans strain 4834-R reveals that flagellar motility is not a general feature of xanthomonads. *BMC Genomics.* **14,** 761 (2013).

430. Potnis, N *et al.* Comparative genomics reveals diversity among xanthomonads infecting tomato and pepper. *BMC Genomics.* **12,** 146 (2011).

431. Kimbrel, JA. Givan, SA. Temple, TN. Johnson, KB. Chang, JH. Genome sequencing and comparative analysis of the carrot bacterial blight pathogen, Xanthomonas hortorum pv.

carotae M081, for insights into pathogenicity and applications in molecular diagnostics. *Mol. Plant Pathol.* **12,** 580-94 (2011).

432. Salzberg, SL *et al.* Genome sequence and rapid evolution of the rice pathogen Xanthomonas oryzae pv. oryzae PXO99A. *BMC Genomics.* **9,** 204 (2008).

433. Simpson, AJ *et al.* The genome sequence of the plant pathogen Xylella fastidiosa. The Xylella fastidiosa Consortium of the Organization for Nucleotide Sequencing and Analysis. *Nature.* **406,** 151-9 (2000).

434. Chen, J *et al.* Draft Genome Sequence of Xylella fastidiosa subsp. fastidiosa Strain Stag's Leap. *Genome Announc.* **4,** (2016).

435. Chen, J. Huang, H. Chang, CJ. Stenger, DC. Draft Genome Sequence of Xylella fastidiosa subsp. multiplex Strain Griffin-1 from Quercus rubra in Georgia. *Genome Announc.* **1,** (2013).

436. Van Sluys, MA *et al.* Comparative analyses of the complete genome sequences of Pierce's disease and citrus variegated chlorosis strains of Xylella fastidiosa. *J. Bacteriol.* **185,** 1018-26 (2003).

437. Savin, C *et al.* Draft Genome Sequence of a Clinical Strain of Yersinia enterocolitica (IP10393) of Bioserotype 4/O:3 from France. *Genome Announc.* **1,** (2013).

438. Wang, X *et al.* Complete genome sequence of a Yersinia enterocolitica "Old World" (3/O:9) strain and comparison with the "New World" (1B/O:8) strain. *J. Clin. Microbiol.* **49,** 1251-9 (2011).

439. Batzilla, J. Höper, D. Antonenka, U. Heesemann, J. Rakin, A. Complete genome sequence of Yersinia enterocolitica subsp. palearctica serogroup O:3. *J. Bacteriol.* **193,** 2067 (2011).

440. Klinzing, DC *et al.* Shotgun genome sequence of a Yersinia enterocolitica isolate from the Philippines. *J. Bacteriol.* **194,** 542-3 (2012).

441. Parkhill, J *et al.* Genome sequence of Yersinia pestis, the causative agent of plague. *Nature.* **413,** 523-7 (2001).

442. Touchman, JW *et al.* A North American Yersinia pestis draft genome sequence: SNPs and phylogenetic analysis. *PLoS ONE.* **2,** e220 (2007).

443. Chance, T *et al.* A spontaneous mutation in kdsD, a biosynthesis gene for 3 Deoxy-D-manno-Octulosonic Acid, occurred in a ciprofloxacin resistant strain of Francisella tularensis and caused a high level of attenuation in murine models of tularemia. *PLoS ONE.* **12,** e0174106 (2017).

444. Cáceres, O *et al.* Whole-Genome Sequencing and Comparative Analysis of Yersinia pestis, the Causative Agent of a Plague Outbreak in Northern Peru. *Genome Announc.* **1,** (2013).

445. Reiss, RA. Guerra, P. Makhnin, O. Metagenome phylogenetic profiling of microbial community evolution in a tetrachloroethene-contaminated aquifer responding to enhanced reductive dechlorination protocols. *Stand Genomic Sci.* **11**, 88 (2016).

446. Accinas, SG *et al*. Metabolic Architecture of the Deep Ocean Microbiome. *bioRxiv*, doi: 10.1101/635680
