# Supplementary material for: Cryptic inoviruses revealed as pervasive in bacteria and archaea across Earth’s biomes
Source: Nat Microbiol. 2019 Jul 22;4(11):1895–906. doi: 10.1038/s41564-019-0510-x (PMC6813254; doi:10.1038/s41564-019-0510-x)
Supplement: Supplementary file 2 — Reporting Summary [file 41564_2019_510_MOESM2_ESM.pdf]

nature research

Corresponding author(s): Simon Roux, Emiley A. Eloe-Fadrosh

Last updated by author(s): 5/28/2019

# Reporting Summary

Nature Research wishes to improve the reproducibility of the work that we publish. This form provides structure for consistency and transparency in reporting. For further information on Nature Research policies, see Authors & Referees and the Editorial Policy Checklist.

## Statistics

For all statistical analyses, confirm that the following items are present in the figure legend, table legend, main text, or Methods section.

| n/a | Confirmed | |
|---|---|---|
| ☒ | ☐ | The exact sample size ($n$) for each experimental group/condition, given as a discrete number and unit of measurement |
| ☒ | ☐ | A statement on whether measurements were taken from distinct samples or whether the same sample was measured repeatedly |
| ☐ | ☒ | The statistical test(s) used AND whether they are one- or two-sided<br>*Only common tests should be described solely by name; describe more complex techniques in the Methods section.* |
| ☐ | ☒ | A description of all covariates tested |
| ☒ | ☐ | A description of any assumptions or corrections, such as tests of normality and adjustment for multiple comparisons |
| ☒ | ☐ | A full description of the statistical parameters including central tendency (e.g. means) or other basic estimates (e.g. regression coefficient) AND variation (e.g. standard deviation) or associated estimates of uncertainty (e.g. confidence intervals) |
| ☐ | ☒ | For null hypothesis testing, the test statistic (e.g. $F$, $t$, $r$) with confidence intervals, effect sizes, degrees of freedom and $P$ value noted<br>*Give P values as exact values whenever suitable.* |
| ☒ | ☐ | For Bayesian analysis, information on the choice of priors and Markov chain Monte Carlo settings |
| ☒ | ☐ | For hierarchical and complex designs, identification of the appropriate level for tests and full reporting of outcomes |
| ☐ | ☒ | Estimates of effect sizes (e.g. Cohen's $d$, Pearson's $r$), indicating how they were calculated |

*Our web collection on statistics for biologists contains articles on many of the points above.*

## Software and code

Policy information about availability of computer code

| | |
|---|---|
| Data collection | Data were collected using a custom set of scripts specifically designed to identify inovirus genomes. These are available at https://github.com/simroux/Inovirus/tree/master/Inovirus_detector |
| Data analysis | For specific reference inoviruses, genes were predicted de novo using Glimmer v3. Sequence similarity searches were conducted using blast+ v2.7.1, hmmer 3.1b2, hhpred (online at https://toolkit.tuebingen.mpg.de/#/tools/hhpred) and hhsearch v2.0.15. Sequences were clustered using InfoMap 0.18.25, mummer 3.0, SDT 1.0, and cd-hit 4.7. Viral sequences (non-inoviruses) were automatically detected using VirSorter v1.0.5. Signal peptide and transmembrane domains were predicted using SignalP 4.1 and TMHMM 2.0c. Trees were built using FastTree2 and IQ-Tree 1.5.5, based on alignments computed with muscle 3.8 or MAFFT v7.294b and automatically trimmed with trimAL v1.4 or BMGE v1.12. Alignments were manually inspected using Jalview v10.0.2. Statistical analyses, sanger sequenced reads interpretation, and automatic classifier design were conducted in R 3.4.1, using the following packages: randomForest, party, glmnet, sangerseqR, sangeranalyseR, and readR. Secondary structure of putative inovirus major capsid proteins were predicted using Phyre v2.0. Figures were generated with R 3.4.1 using the ggplot2 package, Cytoscape v3.6.1, iTOL v4.4.1, and python v3.6.2 using matplotlib v2.0.2. Constraints in sequences to be synthesized were automatically identified and adjusted using BOOST v1.3.3. |

For manuscripts utilizing custom algorithms or software that are central to the research but not yet described in published literature, software must be made available to editors/reviewers. We strongly encourage code deposition in a community repository (e.g. GitHub). See the Nature Research guidelines for submitting code & software for further information.

## Data

Policy information about availability of data

All manuscripts must include a data availability statement. This statement should provide the following information, where applicable:

- Accession codes, unique identifiers, or web links for publicly available datasets
- A list of figures that have associated raw data
- A description of any restrictions on data availability

The following data are available at https://genome.jgi.doe.gov/portal/Inovirus/Inovirus.home.html:

Gb_files_inoviruses.zip: GenBank files of all representative genomes for each inovirus species.
Ref_PCs_inoviruses.zip: Protein clusters from the references (raw fasta, alignment fasta, hmm profile).
iPFs_inoviruses.zip: Protein families from extended inovirus dataset (raw fasta, alignment fasta, hmm profile).
MobM_C_primer_amplicon.fasta: Multiple sequence alignment of the C primer products with Methanolobus MobM genome (NZ_FOUJ01000007) confirming that C primer products span the junction of the excised genome.

# Field-specific reporting

Please select the one below that is the best fit for your research. If you are not sure, read the appropriate sections before making your selection.

☒ Life sciences ☐ Behavioural & social sciences ☐ Ecological, evolutionary & environmental sciences

For a reference copy of the document with all sections, see nature.com/documents/nr-reporting-summary-flat.pdf

# Life sciences study design

All studies must disclose on these points even when the disclosure is negative.

| | |
|---|---|
| Sample size | No sample size calculation was performed, as the largest collection of publicly available data possible was mined. |
| Data exclusions | No data were excluded. |
| Replication | None of the findings was found to be impossible to replicate. This includes PCR amplification of the putative archaeal inovirus provirus, which was repeated either two of three times with similar results (see Supplementary Fig. 11), and the superinfection experiments which were conducted twice and produced similar results. |
| Randomization | None of the analyses involved allocation of samples to different groups. |
| Blinding | None of the analyses required blind investigation since the study does not involve a treatment vs control trial (with the exception of "obvious" negative controls such as "no template" PCR). |

# Reporting for specific materials, systems and methods

We require information from authors about some types of materials, experimental systems and methods used in many studies. Here, indicate whether each material, system or method listed is relevant to your study. If you are not sure if a list item applies to your research, read the appropriate section before selecting a response.

## Materials & experimental systems

| n/a | Involved in the study | |
|---|---|---|
| ☒ | ☐ | Antibodies |
| ☒ | ☐ | Eukaryotic cell lines |
| ☒ | ☐ | Palaeontology |
| ☒ | ☐ | Animals and other organisms |
| ☒ | ☐ | Human research participants |
| ☒ | ☐ | Clinical data |

## Methods

| n/a | Involved in the study | |
|---|---|---|
| ☒ | ☐ | ChIP-seq |
| ☒ | ☐ | Flow cytometry |
| ☒ | ☐ | MRI-based neuroimaging |
